# Cryo-EM structure, enzymatic activity and genome targeting of canonical PRC1

**DOI:** 10.1038/s41594-026-01885-6

**Published:** 2026-09-14

**Authors:** Maria Ciapponi, Martina Cafiso, Sven Schkölziger, Christian Benda, Jacques Bonnet, Jürg Müller

**Affiliations:** 1 Laboratory of Chromatin Biology, Max-Planck Institute of Biochemistry, Martinsried, Germany; 2 Department of Structural Cell Biology, Max-Planck Institute of Biochemistry, Martinsried, Germany

**Keywords:** Structural biology, Developmental biology, Histone post-translational modifications, Ubiquitylation

## Abstract

Canonical Polycomb repressive complex 1 (cPRC1) preserves cell fate decisions by repressing aberrant transcription of developmental regulator genes. We report the cryo-electron microscopy structure of the human cPRC1 holocomplex assembled from RING1B, BMI1, PHC2 and CBX7 bound to an H3K27me3-modified mononucleosome together with the ubiquitin-conjugating enzyme UBCH5C. cPRC1 adopts a compact, highly integrated architecture in which the subunits RING1B, BMI1 and PHC2 form an extended interface that positions UBCH5C on the nucleosome to enable efficient monoubiquitination of histone H2A at K119. This organization is conserved in *Drosophila*, where mutational analyses identify the PHC2 ortholog Polyhomeotic (Ph) as a central scaffold and targeting factor. The Ph HD domain is required for complex assembly, whereas the Ph SAM domain is dispensable for assembly but essential for cPRC1 recruitment to Polycomb target genes and productive H2A monoubiquitination at these loci.

## Abstract (web summary)

Ciapponi et al. determine the cryo-electron microscopy structure of the canonical Polycomb repressive complex 1 holocomplex and show how its architecture coordinates complex assembly, H2A monoubiquitination and genomic targeting.

## Main

Biochemical purification of Polyhomeotic (Ph) protein, a member of the Polycomb group (PcG) of genes in *Drosophila*, originally led to the first isolation of Polycomb repressive complex 1 (PRC1)^1^. In addition to Ph, this multiprotein assembly contained the products encoded by the PcG genes *Pc* (Polycomb), *Psc* (Posterior sex combs) and *Sce* (Sex combs extra), which were all originally identified through genetics because of their role in repressing inappropriate transcription of developmental regulator genes during embryogenesis^2^. In a different approach, a highly similar mammalian complex composed of PHC2, BMI1 (also known as PCGF4) and RING1B, the orthologs of Ph, Psc and Sce, respectively, was isolated through purification of the E3 ubiquitin ligase activity responsible for monoubiquitination of histone H2A at K119 in nucleosomes (H2Aub1)^3^. These studies further identified the RING finger domain of RING1B (or its *Drosophila* counterpart Sce) as essential for PRC1 catalytic activity^3^. In addition to this canonical form of PRC1 (cPRC1), mammals and flies both harbor biochemically and functionally distinct variant PRC1 complexes. In mammals and in flies, variant PRC1 assemblies share RING1B or its paralog RING1A (mammals) or Sce (flies) with cPRC1 but lack the other cPRC1 subunits and incorporate distinct accessory proteins that define the different variant PRC1 subtypes^4–6^. cPRC1 and variant PRC1 complexes contribute in both distinct and overlapping ways to shaping the H2Aub1 genomic landscape and gene repression^7–11^, with cPRC1 exerting essential repressive functions also through ubiquitination-independent chromatin compaction^1,12–20^.

Despite substantial progress in defining PRC1 composition and functional diversification, structural insight into PRC1 architecture has remained limited. Existing structural information is largely confined to individual domains or binary subunit interactions^21–26^. Subsequent structural studies revealed how SAM-domain-mediated interactions among the cPRC1 subunit Ph, its binding partner Scm and Sfmbt—a component of the PhoRC DNA-binding complex—can anchor PRC1 to *cis*-regulatory DNA elements at target genes^27,28^. Importantly, a crystal structure of nucleosome-bound RING1B–BMI1 RING finger heterodimer in which RING1B was fused to the E2 enzyme UBCH5C provided first mechanistic insight into cPRC1-mediated H2AK119 monoubiquitination in chromatin^29^. However, how cPRC1 subunits are arranged within an intact complex, how the holocomplex engages nucleosomes and how catalytic activity is coordinated in this context have remained unresolved, as a structure of a PRC1 holocomplex has so far been lacking.

Here, we used single-particle cryo-electron microscopy (cryo-EM) to determine the structure of human cPRC1, comprising RING1B, BMI1, PHC2 and CBX7, bound to a mononucleosome in complex with the ubiquitin-conjugating enzyme UBCH5C. For simplicity, we refer to the RING1B–BMI1–PHC2–CBX7 assembly, also known as PRC1.4, as PRC1. The structure, together with complementary biochemical analyses, reveals three key principles underlying PRC1 architecture and function. First, PRC1 adopts a compact, highly integrated architecture stabilized by an extensive network of intersubunit interactions. Second, PHC2 cooperates with RING1B and BMI1 to form an extended interface that stably positions UBCH5C on the nucleosome, thereby strongly enhancing H2AK119 monoubiquitination. Third, in *Drosophila*, PRC1 recruitment to Polycomb target genes and the resulting H2A monoubiquitination critically require the SAM domain of Ph. PHC2 in humans and Ph in *Drosophila*, thus, act as a central link of complex assembly, enzymatic activity and genomic targeting of PRC1.

## Results

### PRC1 is a highly active E3 ligase

**Fig. 1:**
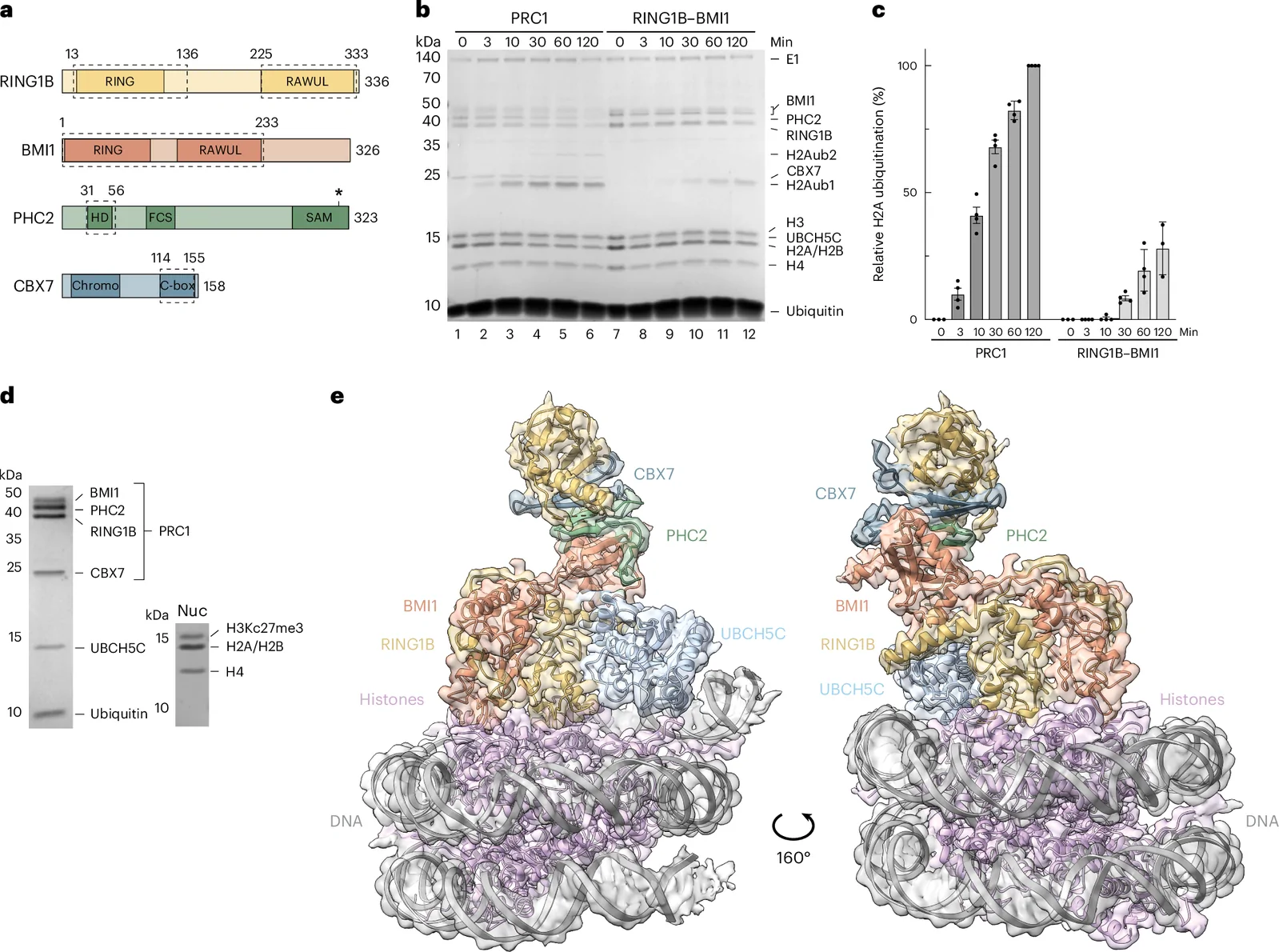
E3 ligase activity and cryo-EM structure of nucleosome-bound tetrameric PRC1 in complex with UBCH5C. **a**, Domain organization of subunits used for PRC1 reconstitution. In the PHC2 SAM domain, the asterisk indicates the L307E;L311E substitutions used to prevent SAM domain oligomerization. Dashed boxes indicate protein regions built into the cryo-EM density map. **b**, Time-course ubiquitination assay on mononucleosomes showing formation of H2Aub1 and H2Aub2 by PRC1 and RING1B–BMI1. Reactions were analyzed on a 16% polyacrylamide gel and proteins were visualized by Coomassie staining. Four independent assays were performed and quantification of the results is shown in **c**. **c**, Quantification of H2A ubiquitination reactions shown in **b** by densitometry from independent experiments (*n* = 4). For each experiment, the summed H2Aub1 and H2Aub2 signal in lane 6 was set to 100% and used for quantification of summed H2Aub1 and H2Aub2 signals in other lanes on the same gel. Dots represent individual data points; histogram bars represent mean values ± s.d. **d**, Coomassie-stained gels showing the sample with recombinant PRC1, UBCH5C and ubiquitin (left) that was mixed at a 10:1 ratio with H3Kc27me3 mononucleosomes (right) and applied to grids. **e**, Two views of the cryo-EM reconstruction at 2.7 Å and model of the nucleosome-bound PRC1–UBCH5C complex, showing RING1B, BMI1, PHC2_HD_, CBX7_C-box_ and UBCH5C bound to one face of the nucleosome. Source data

**Extended Data Fig. 1:**
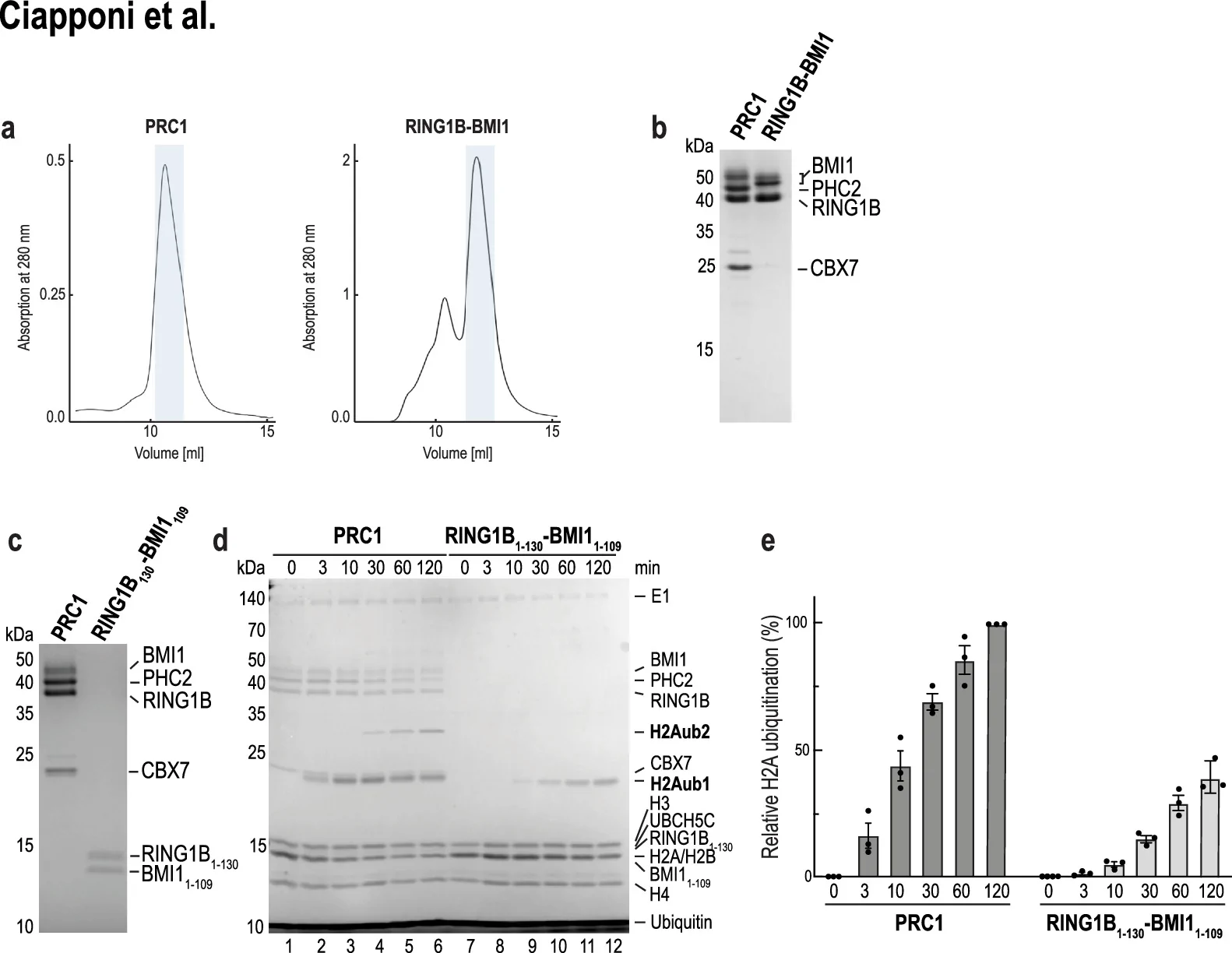
Preparation of the human PRC1 holocomplex, full-length RING1B-BMI1 heterodimer, and minimal RING1B-BMI1 RING finger heterodimer for H2A ubiquitination assays. **(a)** Gel filtration profiles of the reconstituted PRC1 holocomplex (left) and the full-length RING1B-BMI1 dimer used in Fig. 1b (right). The fractions used are highlighted in blue. **(b)** Coomassie^−^stained gel of reconstituted PRC1 and full length RING1B-BMI1 used in Fig. 1b. Comparable complex integrities was observed in multiple independent purifications (n ≥ 3 for either complex). **(c)** Coomassie^−^stained gel of reconstituted PRC1 and RING1B_1-130_-BMI1_1-109_ Ring finger heterodimer used as E3 ligases in (**d**). Comparable complex integrity was observed in multiple independent purifications (n ≥ 3 for either complex). **(d)** Time-course ubiquitination assay on mononucleosomes showing formation of H2Aub1 and H2Aub2 by PRC1 and RING1B_1-130_-BMI1_1-109_ Ring finger heterodimer. Reactions were analysed on a 16% polyacrylamide gel and proteins were visualized by Coomassie-staining. Three independent assays were performed and quantification of the results is shown in (**e**). **(e)** Quantification of H2A ubiquitination reactions shown in (**d**) by densitometry from independent experiments (n = 3). Quantification in summed H2Aub1 and H2Aub2 signal in lane 6 was set to 100% and used for quantification of summed H2Aub1 and H2Aub2 signals in the other lanes on the same gel. Dots represent individual data points; histogram bars represent mean values +/– SD. Source data

We purified recombinant human PRC1 reconstituted from the RING1B, BMI1, PHC2 and CBX7 isoforms depicted in Fig. 1 (Fig. 1a and Extended Data Fig. 1a,b). To assess the E3 ligase activity of this complex, we compared its ability to ubiquitinate reconstituted *Xenopus* mononucleosomes with that of a purified heterodimer consisting of full-length RING1B and BMI1 (Fig. 1b and Extended Data Fig. 1a,b). The RING finger heterodimer formed by RING1B and BMI1 has long been considered to constitute the catalytic core of PRC1 (refs. ^23,24,29–31^). In time-course assays, PRC1 catalyzed near-complete H2AK119 monoubiquitination within 10 min, followed by the appearance of diubiquitinated H2A at later time points (Fig. 1b,c, lanes 1–6). In contrast, the RING1B–BMI1 heterodimer produced detectable H2Aub1 only after 10 min and robust but still incomplete monoubiquitination was observed only after 60 min (Fig. 1b,c, lanes 7–12). No diubiquitinated H2A was detected at any time point (Fig. 1b, lanes 7–12). Quantification of total ubiquitin conjugation to H2A, based on combined H2Aub1 and H2Aub2 signals, is shown in Fig. 1c. For comparison, the corresponding time-course analysis of the minimal RING1B–BMI1 RING finger heterodimer is provided in Extended Data Fig. 1c–e, corroborating previous observations that this minimal heterodimer exhibits higher E3 ligase activity than the full-length RING1B–BMI1 heterodimer^23,24^. Taken together, these results show that tetrameric PRC1 is markedly more active as an E3 ligase than either the full-length RING1B–BMI1 heterodimer or the minimal RING finger heterodimer.

### Overview of the nucleosome-bound PRC1–UBCH5C complex

**Extended Data Fig. 2:**
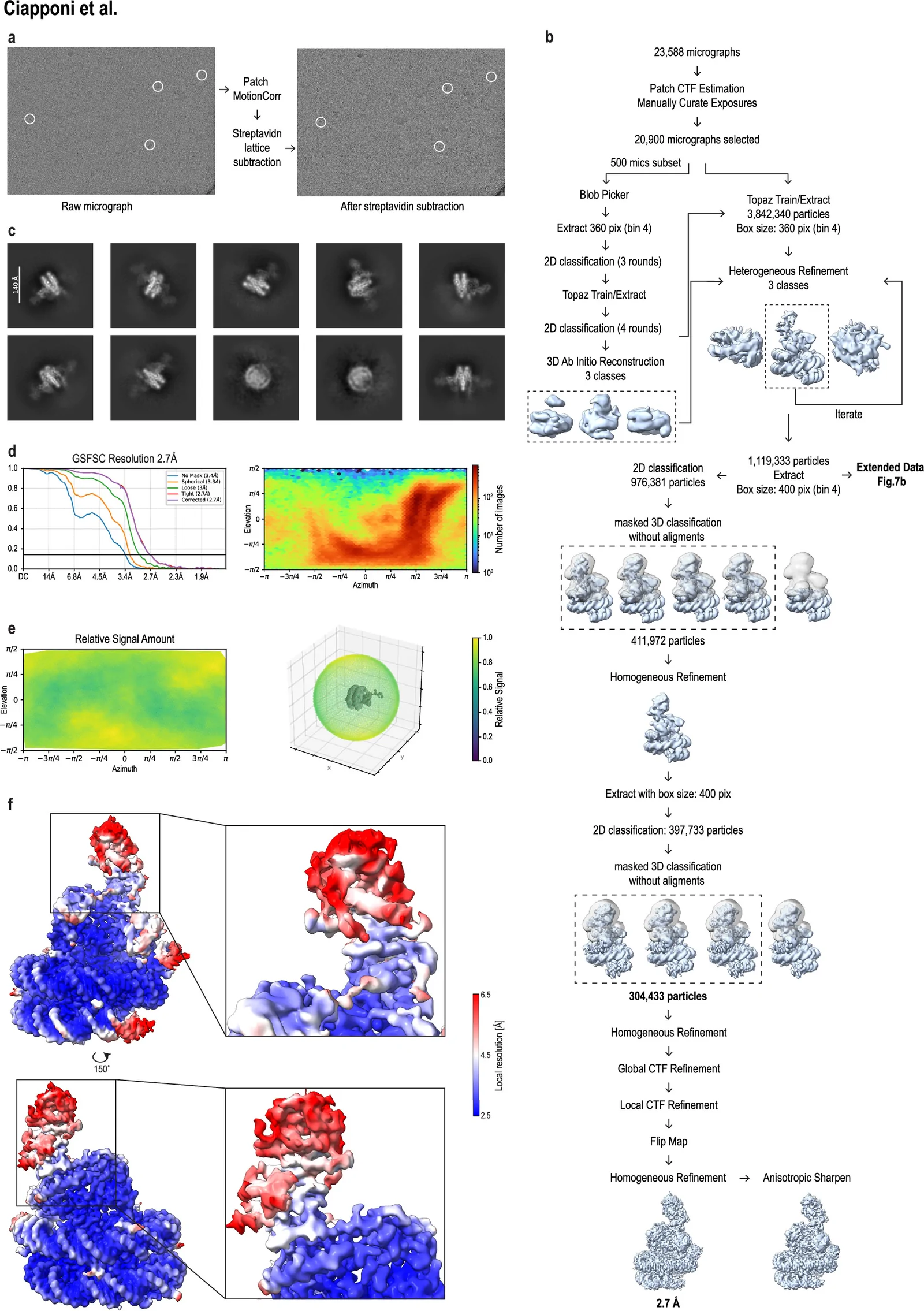
Cryo-EM analysis of nucleosome-bound PRC1 in complex with UBCH5C. **(a)** Representative raw micrograph before (left) and after (right) motion correction and streptavidin lattice subtraction. Representative particles are indicated with circles. **(b)** Cryo-EM data-processing workflow, as described in Methods. **(c)** Representative 2D class averages of nucleosomes displaying extra densities on one or both faces of the nucleosome disc. **(d)** Gold-standard Fourier shell correlation (GSFSC) curve (left) and particle angular distribution plot (right) from CryoSPARC. Colors indicate the number of particles per viewing direction, ranging from blue (low) to red (high). **(e)** Relative signal shown in a 2D angular distribution (azimuth–elevation) plot (left) and a 3D colored scatter plot (right), overlaid with a low-pass filtered volume. Darker regions correspond to lower relative signal, indicating under-sampled viewing directions. Plots were generated using CryoSPARC. **(f)** Local-resolution estimation of the final sharpened map shown in two orientations related by a 150° rotation around the vertical axis. Enlarged view of the β-sheet interface in the PRC1 assembly is shown on the right. Colors range from blue (high resolution) to red (low resolution).

For cryo‑EM analysis, the PRC1 complex was assembled with free human UBCH5C, ubiquitin and 5′‑biotinylated *Xenopus* mononucleosomes containing histone H3 bearing a trimethyl‑lysine analog at position 27 (H3Kc27me3) (Fig. 1d). The sample was applied to streptavidin‑coated grids (Extended Data Fig. 2a) and analyzed by single‑particle cryo‑EM. Two-dimensional (2D) class averages revealed well-defined densities on one face of the nucleosome disc or, symmetrically, on both faces (Extended Data Fig. 2b,c). Because of nucleosome symmetry, three‑dimensional (3D) reconstruction yielded a preferentially resolved density on a single nucleosomal surface. The final reconstruction reached a global resolution of 2.7 Å, enabling unambiguous fitting of the nucleosome core, previously determined structures of individual PRC1 domains and UBCH5C (Fig. 1e, Extended Data Fig. 2b–f and Table 1). Importantly, density was resolved for the conserved domains of all four core PRC1 subunits, with the exception of the PHC2 FCS and SAM domains and the CBX7 chromodomain (Fig. 1a). No density corresponding to ubiquitin was observed.

**Table 1:** Cryo-EM data collection, refinement and validation statistic

	PRC1–UBCH5C–Nuc (EMD-56669, PDB 28OE)	PRC1–Nuc (EMD-56717)
**Data collection and processing**		
Magnification	105,000	105,000
Voltage (kV)	300	300
Electron exposure	60.45 e^−^ per Å^2^	55.8 e^−^ per Å^2^
Defocus range (μm)	−0.6 to −2.2	−0.5 to −2
Pixel size (Å)	0.8512	0.8512
Symmetry imposed	*C*_1_	*C*_1_
Initial particle images (no.)	3,842,340	3,778,540
Final particle images (no.)	304,433	33,810
Map resolution (Å) at FSC = 0.143	2.7	3.7
FSC threshold	0.143	0.143
Map resolution range (Å)	2.5–6.5	3.5–9.0
**Refinement**		
Initial model used (PDB codes)	2NZD, 4R8P, 2NA1, 3GS2, AF3 model	
Model resolution (Å) at 0.143 FSC	2.6 (unmasked)	
FSC threshold	0.143	
Map sharpening *B* factor (Å^2^)*	−100.10	
Model composition		
Nonhydrogen atoms	17,633	
Residues	Protein: 1,428; nucleotide: 302	
Ligands	Zn: 4	
B factors (Å^2^)		
Protein	151.09	
Nucleotide	152.94	
Ligand	188.41	
Root-mean-square deviations		
Bond lengths (Å)	0.005	
Bond angles (°)	0.663	
**Validation**		
MolProbity score	1.56	
Clashscore	4.42	
Rotamer outliers (%)	0.00	
Ramachandran plot		
Favored (%)	95.19	
Allowed (%)	4.81	
Disallowed (%)	0.00	

Model refinement was conducted against the anisotropic sharpened map (PHENIX^48^).

*Effective average *B* factor (Å^2^) after anisotropic map sharpening performed in PHENIX.

**Extended Data Fig. 3:**
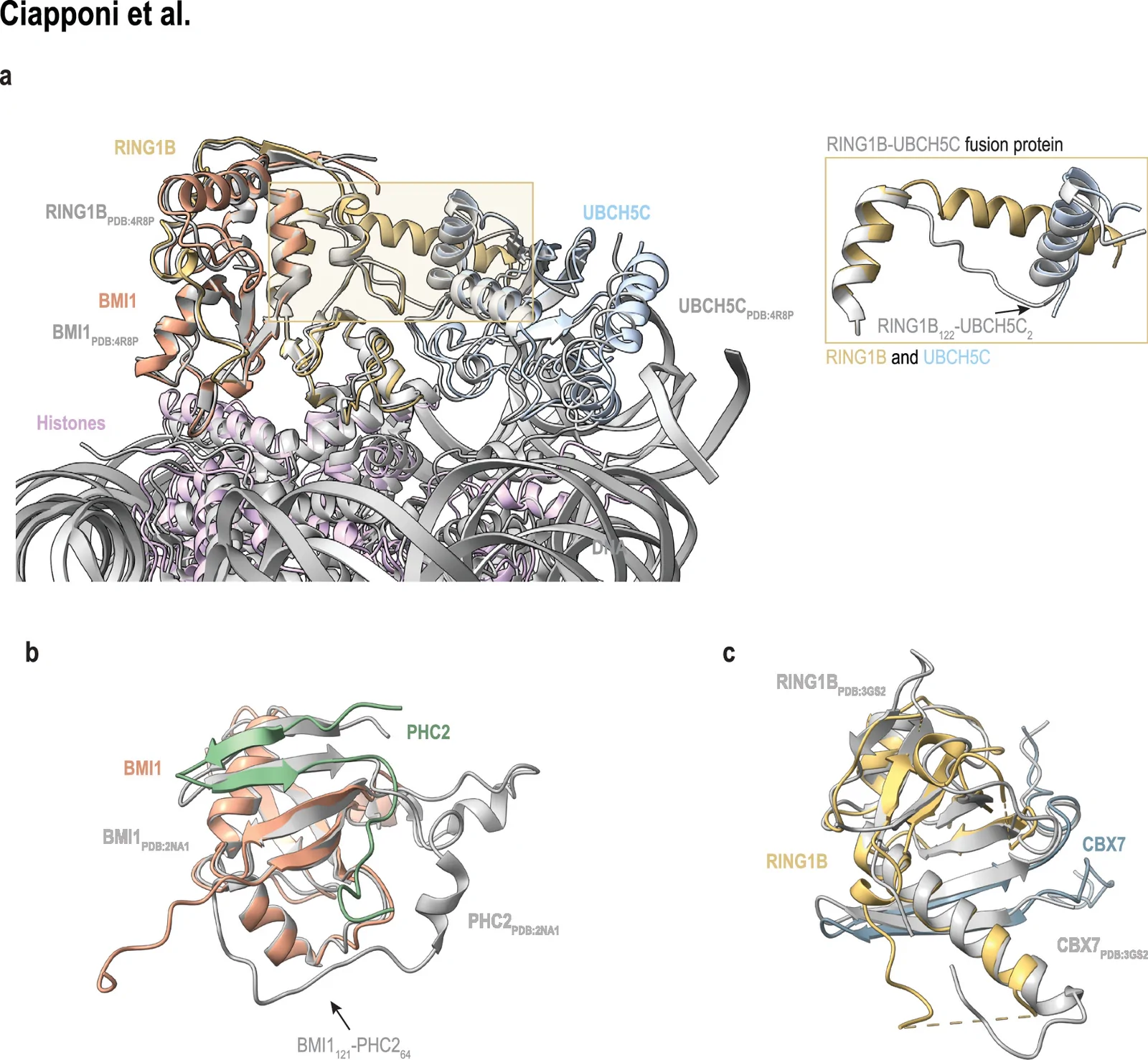
Comparison of domain organization and interaction interfaces in holo-PRC1 with previously reported structures. **(a)** Superimposition of the RING1B_2–136_-BMI1_2–109_-UBCH5C module within the nucleosome-bound PRC1 holocomplex determined here (colored as in Fig. 1e) with the crystal structure of the minimal nucleosome-bound RING1B_2–116_-BMI1_2–109_ heterodimer, in which UBCH5C was genetically fused to RING1B (gray; the fusion point is indicated, PDB: 4R8P ref. ^29^). In the structure reported by McGinty et al.^29^, one RING1B_2–116_–BMI1_2–109_–UBCH5C module binds to each face of the nucleosome. UBCH5C is positioned closer to the nucleosomal DNA on one face of the nucleosome (the proximal side) than on the opposite face (the distal side). Our structure aligns closely with the distal-side configuration. Structures were superimposed in UCSF Chimera (MatchMaker) by aligning the BMI1 chains, yielding an RMSD of 1.54 Å over 105 Cα atoms; residues not present in both structures were excluded. **(b)** Superimposition of the BMI1 RAWUL (RING1B_124-233_) and PHC2 HD (PHC2_28-69_) domains from the cryo-EM structure reported here (colored as in Fig. 1e) with the previously published NMR structure (in gray), in which the BMI1 RAWUL was genetically fused to PHC2 HD (the fusion point is indicated; PDB: 2NA1 ref. ^25^). The core regions of the two domains align closely, whereas deviations are apparent in the segment corresponding to the fusion junction in the NMR construct. In our structure, this region may be further stabilized by the proximity of UBCH5C (Fig. 3a). Structures were superimposed in UCSF Chimera (MatchMaker) by aligning the best-matching chain (BMI1), yielding an RMSD of 0.929 Å over 81 Cα atoms; residues not present in both structures were excluded. **(c)** Superimposition of the RING1B RAWUL (RING1B_225-333_) and CBX7 C-box (CBX7_111-156_) domains from the cryo-EM structure reported here (colored as in Fig. 1e) with the previously published crystal structure of these domains in isolation (shown gray, PDB: 3GS2 ref. ^26^). The two structures align almost completely. Structures were superimposed in UCSF Chimera (MatchMaker) by aligning the best-matching chain (BMI1), yielding an RMSD of 1.068 Å over 62 Cα atoms; residues not present in both structures were excluded.

On the nucleosome surface, we identified well-resolved density that accommodates the previously reported crystal structure of the minimal RING1B_2–116_–BMI1_2–109_ heterodimer fused to UBCH5C^29^ (Fig. 1e and Extended Data Fig. 3a). On top of the RING finger heterodimer, density corresponding to the BMI1 RAWUL domain was observed positioned near the backside of UBCH5C (Fig. 1e). The BMI1 RAWUL domain engages the PHC2 HD domain (PHC2_31–56_), validating the previously reported NMR structure of a BMI1_RAWUL_–PHC2_HD_ fusion protein^25^ (Fig. 1e and Extended Data Fig. 3b). Additional density was assigned to the RING1B RAWUL domain (RING1B_225–333_) in complex with the CBX7 C‑box domain (CBX7_114–155_), consistent with the reported crystal structure^26^ (Fig. 1e and Extended Data Fig. 3c). Notably, our structure uncovers an unexpected organization of these domains into a tightly integrated, compact architecture.

**Fig. 2:**
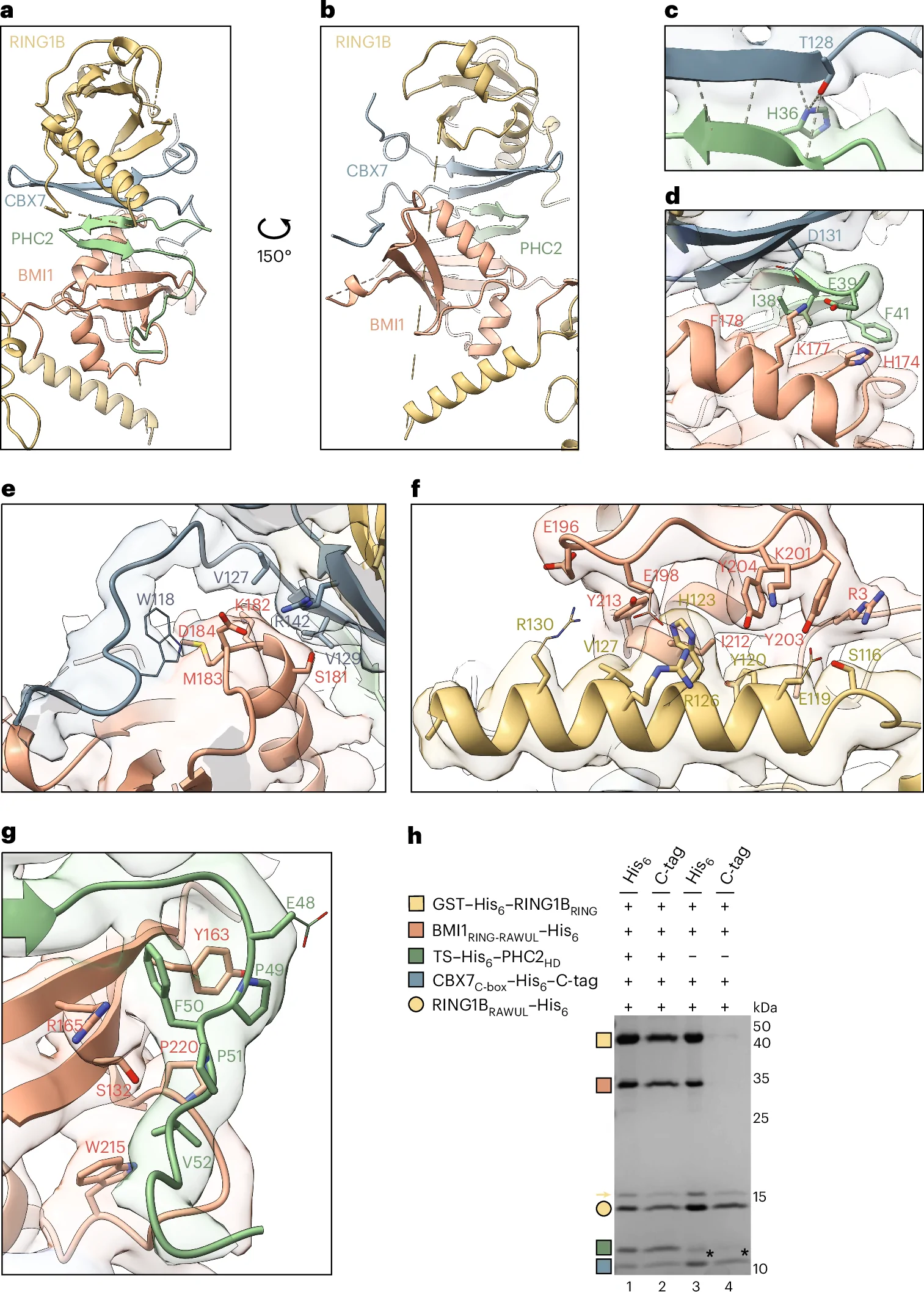
PRC1 subunits form a compact, highly integrated assembly. **a**,**b**, Two views of the PRC1 upper-lobe model, basal (**a**) and rotated by 150° (**b**), showing BMI1_RAWUL_ (BMI1_124–233_), the RING1B helical extension (RING1B_116–136_) and RING1B_RAWUL_ (RING1B_225–333_), CBX7_C-box_ (CBX7_114–155_) and PHC2_HD_ (PHC2_31–56_). These domains are highly conserved between human paralogs and in *Drosophila* orthologs (Extended Data Fig. 4). **c**, Close-up view from **a** showing the PHC2–CBX7 interaction through a parallel β-sheet arrangement likely stabilized by an interstrand hydrogen-bonding network and a hydrogen bond between PHC2 H36 and CBX7 T128. **d**, Zoomed-in view from **b** showing hydrophobic packing of PHC2 I38 and F41 against BMI1 F178 and H174 and an electrostatic network formed by PHC2 E39, BMI1 K177 and CBX7 D131. Side chains with weak or unresolved density (D131) are represented as lines. **e**, Zoomed-in view from **b** of the CBX7–BMI1 interface. Although local resolution in this region is limited, CBX7 W118 appears central for contacts with BMI1 M183 and D184, CBX7 V127 interacts with BMI1 K182 and V187 and CBX7 R142 engages BMI1 S181. Side chains with weak or unresolved density are represented as lines. **f**, Zoomed-in view from **b** showing the interface between the BMI1_RAWUL_ domain and the RING1B 116–136 helix. Key residues mediating contacts include RING1B Y120, H123, R126 and V127 with BMI1 I212, E198 and Y213, together with an interaction between RING1B S116 and BMI1 R3. Although the side chain of RING1B E119 is unresolved, the proximity of the backbone suggests potential interactions with the charged patch on BMI1 formed by K201, Y203 and Y204. Side chains with weak or unresolved density are represented as lines. **g**, Zoomed-in view from **a** showing contacts between the highly conserved (Extended Data Fig. 4) PHC2 E48-P49-F50-P51-V52 motif and the BMI1_RAWUL_ surface. **h**, PRC1 can be reconstituted with minimal subunit fragments. Split RING1B comprising the RING (RING1B_1__–165_) and RAWUL (RING1B_225__–333_) domains was coexpressed with BMI1_RING-RAWUL_ (BMI1_1–232_) and CBX7_C-box_ (CBX7_111__–156_), in the presence (lanes 1 and 2) or absence (lanes 3 and 4) of PHC2_HD_ (PHC2_28__–69_). Proteins were affinity-purified either through the His_6_ tag present in all polypeptides (lanes 1 and 3) or through the C-tag on CBX7 (lanes 2 and 4) and eluates were separated on a polyacrylamide gel and visualized by Coomassie staining. The yellow arrow marks phosphorylated RING1B_RAWUL_. Asterisks in lanes 3 and 4 indicate phosphorylated CBX7; in lanes 1 and 2, this band comigrates with TS–His_6_–PHC2_HD_. Comparable results were obtained in three repeats of the experiment. Source data

**Extended Data Fig. 4:**
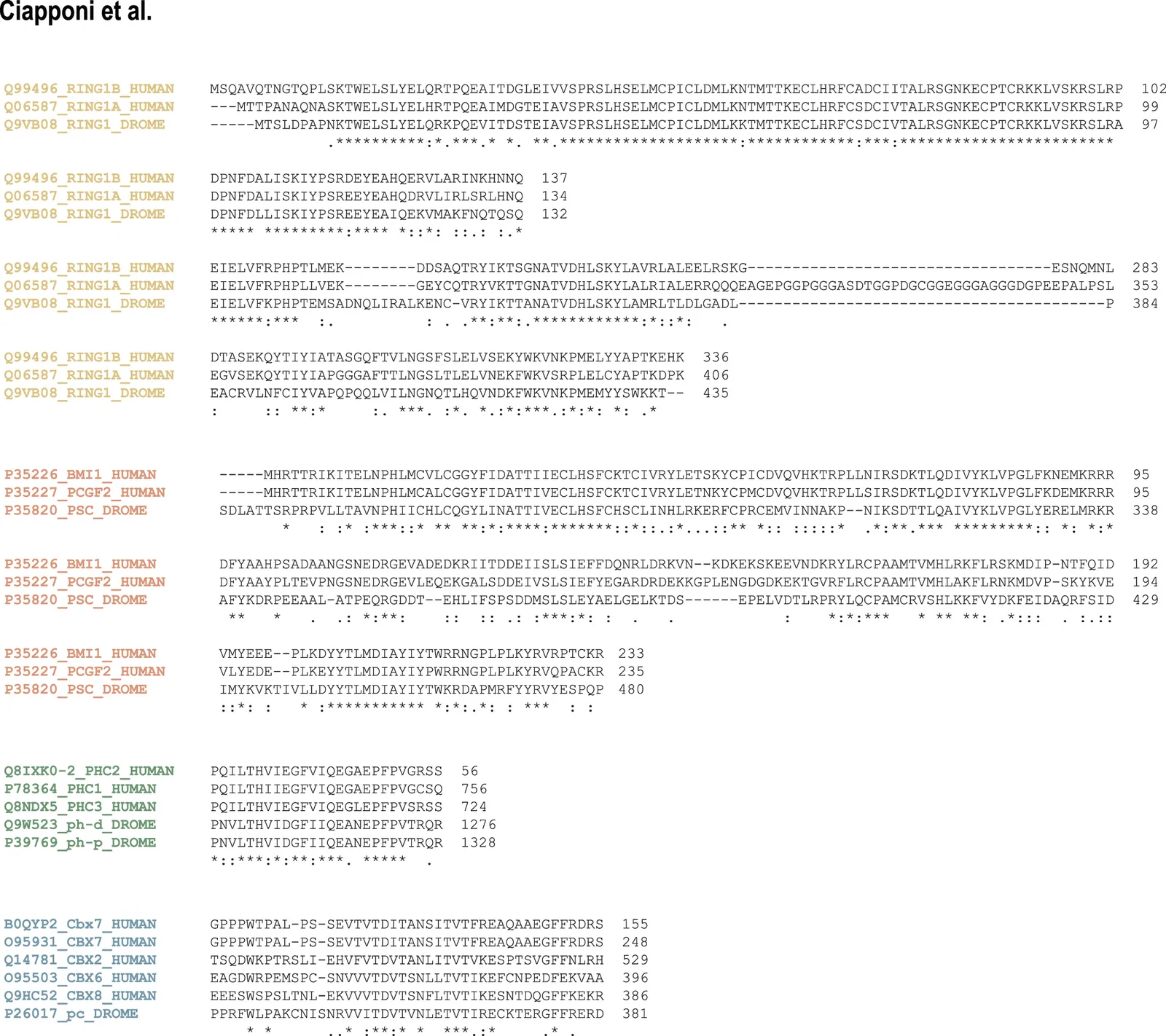
Alignment of human and *Drosophila* PRC1 subunit sequences corresponding to regions visualized in the human PRC1 holocomplex structure. Sequence alignments were generated using Clustal Omega^74^.

An extended intermolecular β‑sheet network stabilizes interactions between the four PRC1 subunits, forming the architectural core of the complex (Fig. 2a,b). In particular, the RING1B–CBX7 module directly engages the BMI1–PHC2 module through an interface formed by CBX7 and PHC2 (Fig. 2a,b). The model suggests that this extended β-sheet is not only formed through backbone interactions but also involves specific side‑chain contacts between conserved residues (Fig. 2c,d and Extended Data Fig. 4). In addition, the BMI1 RAWUL domain uses two distinct interfaces to engage with both the N-terminal loop of the CBX7 C-box domain (Fig. 2e) and RING1B residues 116–136 that form a helical extension of the RING finger domain (Fig. 2f). An additional interface is provided by PHC2 residues P49, F50, P51 and V52, which extend across a surface of the BMI1 RAWUL domain previously proposed to participate in binding to the HD domains of PHC paralogs^25,32^ (Fig. 2g).

Evidence that this interaction network is sufficient to drive PRC1 assembly comes from experiments in which we coexpressed BMI1_RING-RAWUL_, PHC2_HD_, CBX7_C-box_ and split RING1B protein (RING1B_RING_ and RING1B_RAWUL_) to make RING1B_RAWUL_ and CBX7_C-box_ incorporation fully dependent on formation of the β‑sheet assembly. Affinity purification of tagged CBX7_C-box_ efficiently recovered the intact complex (Fig. 2h, lane 2), whereas omission of PHC2_HD_ resulted in copurification of only RING1B_RAWUL_ with CBX7_C-box_ (Fig. 2h, lane 4).

**Extended Data Fig. 5:**
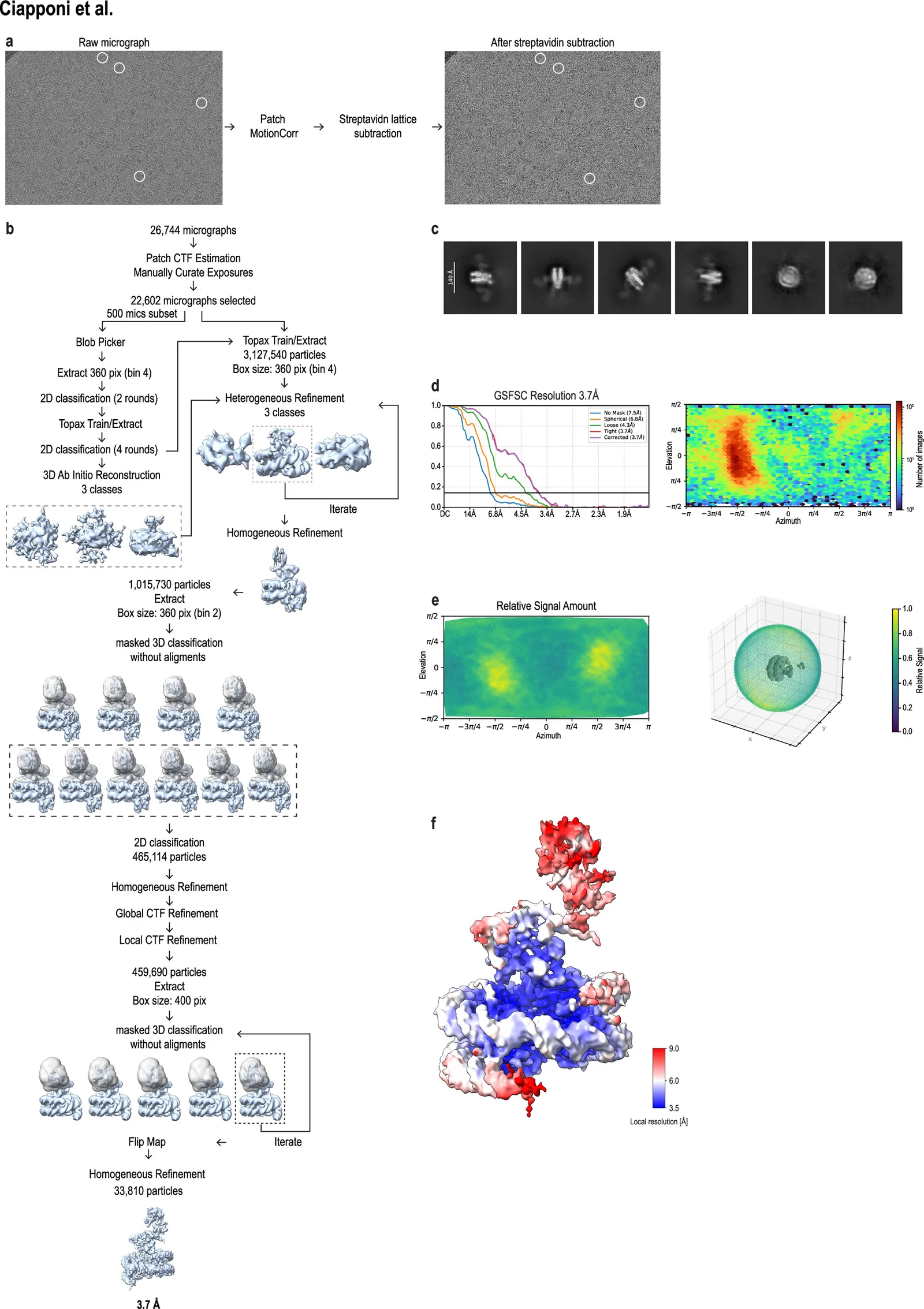
Cryo-EM analysis of PRC1 bound to a nucleosome in the absence of UBCH5C. **(a)** Representative raw micrograph before (left) and after (right) motion correction and streptavidin lattice subtraction. Representative particles are indicated with circles. **(b)** Cryo-EM data‑processing workflow, as described in Methods. **(c)** Representative 2D class averages of nucleosomes displaying one or two side‑bound densities. The upper lobe of PRC1 appears more flexible in this dataset than in the complex containing UBCH5C (Extended Data Fig. 2c). **(d)** Gold-standard Fourier shell correlation (GSFSC) curve (left) and particle angular distribution plot (right) from CryoSPARC. Colors indicate the number of particles per viewing direction, ranging from blue (low) to red (high). **(e)** Relative signal shown in a 2D angular distribution (azimuth–elevation) plot (left) and a 3D colored scatter plot (right), overlaid with a low-pass filtered volume. Darker regions correspond to lower relative signal, indicating under-sampled viewing directions. Plots were generated using CryoSPARC. **(f)** Local‑resolution estimation of the final map. Colors range from blue (high resolution) to red (low resolution). The resolution of the PRC1 upper lobe is estimated to be ~6–9 Å, lower than in the complex containing UBCH5C ( ~ 4–6 Å; Extended Data Fig. 2f).

Lastly, we note that the conformation of the upper lobe of PRC1 is stabilized in the presence of UBCH5C. Determination of the structure of PRC1 bound to a H3Kc27me3 mononucleosome in the absence of UBCH5C permitted a reconstruction in which PRC1 architecture was overall similar to the structure described above but the upper lobe of the complex displayed extensive conformational flexibility (Extended Data Fig. 5 and Table 1).

### An extended RING1B–BMI1–PHC2 interface positions UBCH5C

**Fig. 3:**
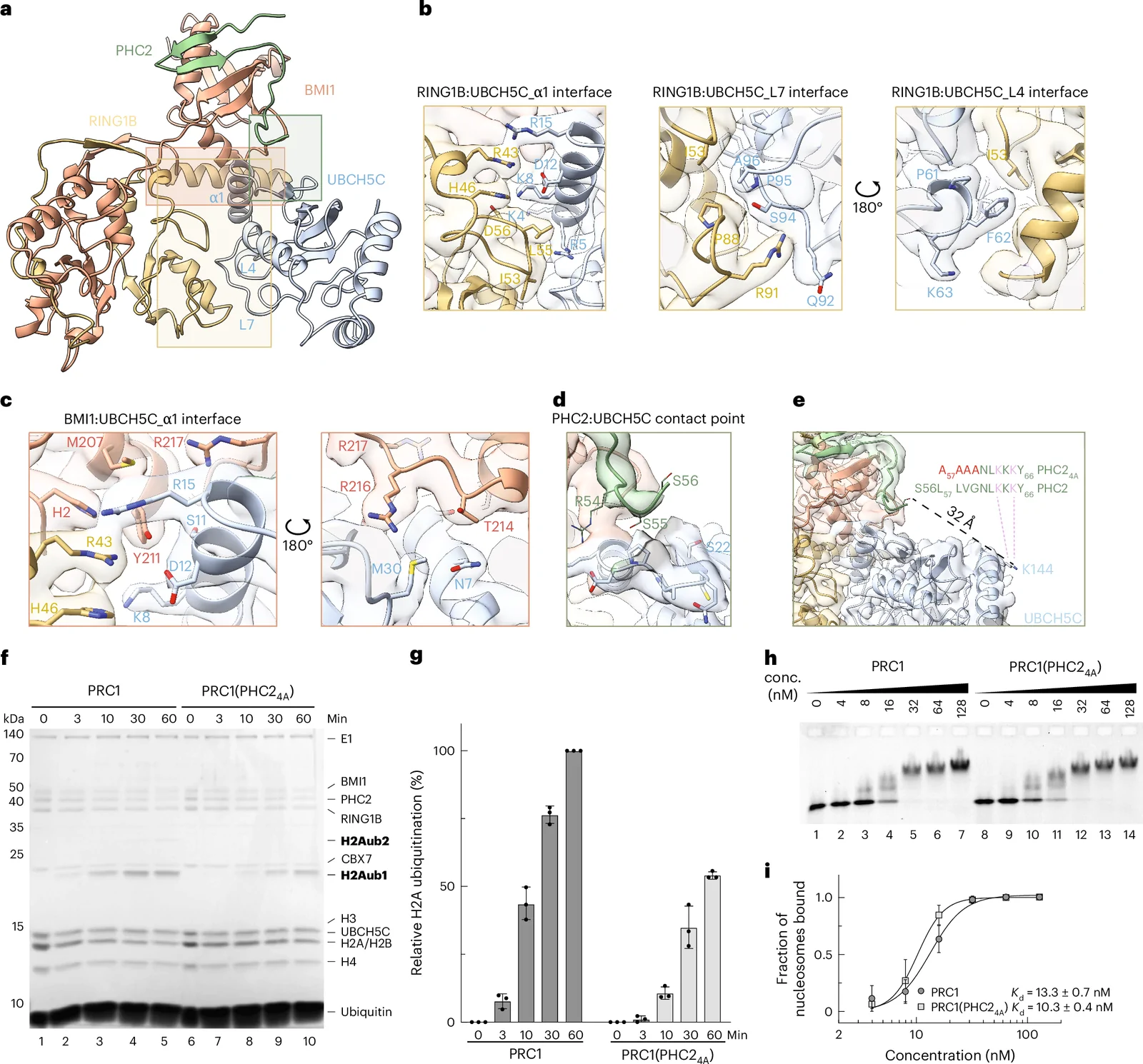
RING1B, BMI1 and PHC2 form a composite UBCH5C interface essential for effective PRC1 catalytic activity. **a**, Model of the PRC1 catalytic core with RING1B_13–136_, BMI1_1–233_ and PHC2_28__–56_ in complex with UBCH5C. Boxes indicate UBCH5C interfaces with RING1B (yellow), BMI1 (orange) and PHC2 (green), with zoomed-in versions shown in **b**–**d**, respectively. Previously reported UBCH5C regions (α1 helix, loop 4 and loop 7) that contact RING1B are indicated^30^. **b**, RING1B:UBCH5C; overlap with the previously reported structure^30^ is shown in Extended Data Fig. 6a. **c**, BMI1:UBCH5C interface. **d**, Potential PHC2:UBCH5C contact points. Regions of unresolved density are colored in gray; side chains with weak or unresolved density are shown as lines. **e**, Distance between PHC2 S56 and UBCH5C K144, which crosslinks to PHC2 K63 and K65. The sequence of PHC2 residues L57 to Y66 is shown, with crosslinked lysines highlighted in pink. Distances were measured in ChimeraX^49^. The corresponding sequence of the PHC2_4A_ mutant is shown, where the LLVG motif is substituted to AAAA (alanines are highlighted in red). **f**, Time‑course ubiquitination assays on mononucleosomes showing formation of H2Aub1 and H2Aub2 by PRC1 compared with PRC1(PHC2_4A_). H2Aub species are indicated in bold. Three independent assays were performed and quantification of the results is shown in **g**. **g**, Quantification of H2A ubiquitination reactions shown in **f** by densitometry from independent experiments (*n* = 3). Quantification of the summed H2Aub1 and H2Aub2 signal in lane 5 was set to 100%, with quantification was performed as in Fig. 1c. Dots represent individual data points; histogram bars represent the mean values ± s.d. **h**, Binding reactions with PRC1 or PRC1(PHC2_4A_) and 0.2 nM unmodified mononucleosomes assembled on a 3′-ATTON647-labeled 215-bp Widom 601 DNA fragment, analyzed by EMSA on a 1.0% agarose gel. Five independent assays were performed and are quantified in **i**. **i**, Quantification of EMSA data by densitometry of ATTO‑647N signal from independent experiments (*n* = 5). Data are presented as the mean values ± s.d.; apparent *K*_d_ values are shown. Source data

Previous structural studies indicated that UBCH5C engages exclusively with the RING domain of RING1B. In contrast, our structure reveals that BMI1 also directly contributes to UBCH5C engagement and further suggests a potential interface between PHC2 and UBCH5C (Fig. 3a).

**Extended Data Fig. 6:**
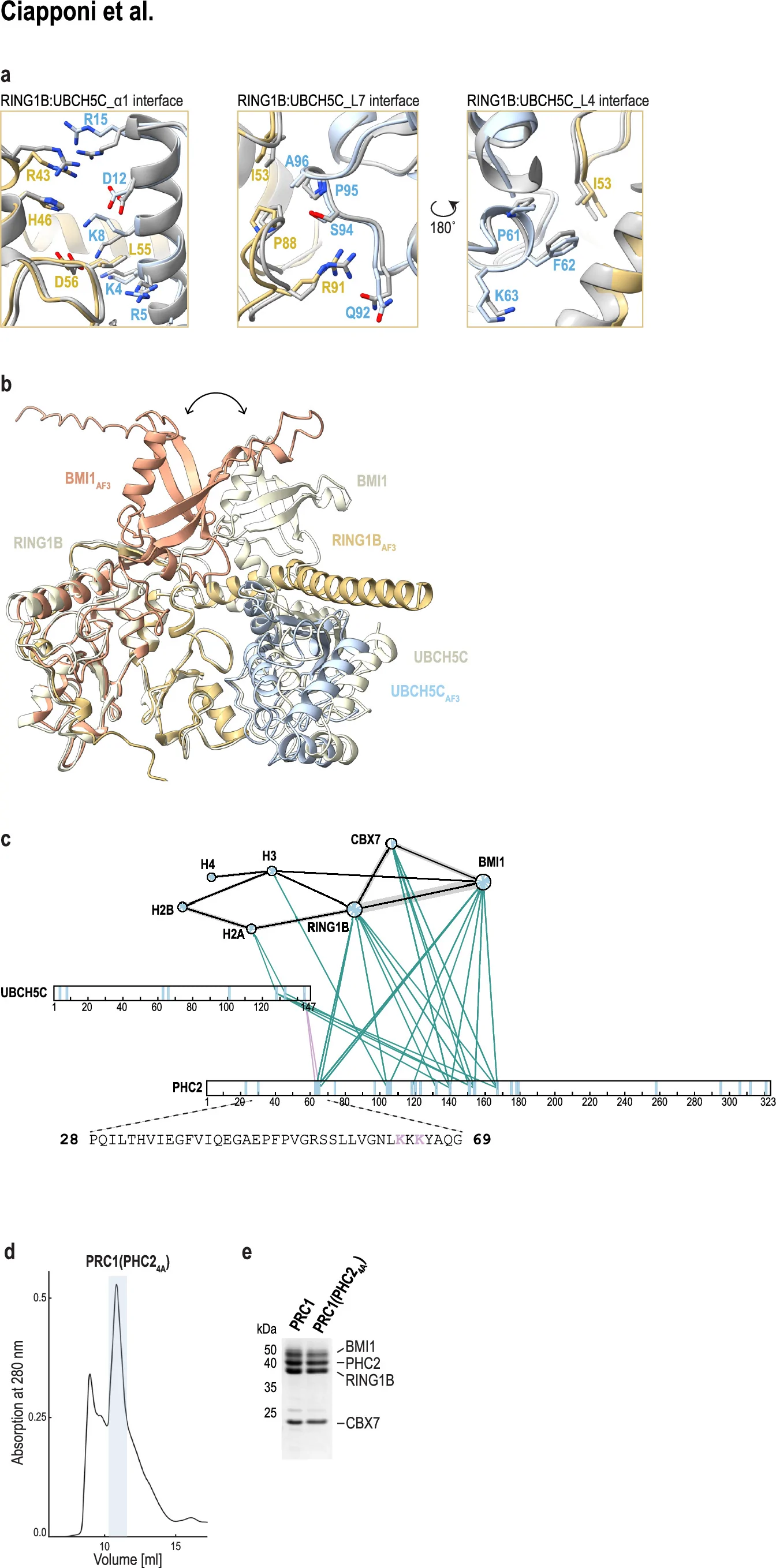
Analysis of PRC1–UBCH5C interactions. **(a)** Superimposition of the RING1B:UBCH5C interface from the cryo-EM structure reported here (colored as in Fig. 1e) with the crystal structure of the isolated RING1B-BMI1 Ring finger heterodimer in complex with UBCH5C (shown in gray, PDB: 3RPG ref. ^30^). The three previously defined UBCH5C contact regions – the α1 helix (left), loop 4 (middle), and loop 7 (right) – align almost perfectly at the RING1B interface. In our structure, however, the side chain of UBCH5C Lys8 adopts a distinct conformation and does not contact RING1B Asp56. This shift may be stabilized by the adjacent BMI1 RAWUL domain, which was absent from the earlier structure^30^. Structures were superimposed in UCSF Chimera (MatchMaker) by aligning the best-matching chain (UBCH5C), yielding an RMSD of 0.876 Å over 143 Cα atoms; residues not present in both structures were excluded. **(b)** Superimposition of the AF3 prediction (colored as in Fig. 1e) of BMI1 (BMI1_1–233_), the RING1B RING domain with its helical extension (RING1B_1–165_), and UBCH5C with the corresponding regions from the cryo-EM structure determined here (pale yellow). AF3 accurately predicts the folding of individual domains; however, the linker between the BMI1 RING and RAWUL domains is not modelled in an extended conformation, resulting in an altered orientation of the RAWUL domain. Although the RING1B helical extension is also predicted, it adopts a different position, contacting UBCH5C and therefore clashing with the BMI1 RAWUL domain observed in our structure. **(c)** Cross-linking mass-spectrometry analysis of PRC1 in complex with UBCH5C and mononucleosomes. The sample was cross-linked using BS3. Identified cross links of PHC2 and UBCH5C with other proteins are depicted as green lines, with cross-links between PHC2 residues Lys63 and Lys65 and UBCH5C Lys144 highlighted in purple. Black lines indicate the cumulative cross-links detected among the other proteins in the sample. Lysine residues in PHC2 and UBCH5C are indicated in blue. Only cross-links reproducibly detected in all technical replicates (n = 3) are shown. **(d)** Gel-filtration profiles of the reconstituted PRC1(PHC2_4A_) complex used for the biochemical assays in Fig. 3f-i. The fractions used are highlighted in blue. **(e)** Coomassie-stained gel of reconstituted PRC1 and PRC1(PHC2_4A_) used in Fig. 3f-i. The mutant complex was purified once. Source data

The RING domain of RING1B interacts with the α1 helix, loop 4 and loop 7 of UBCH5C in a manner consistent with the previously reported crystal structure of a RING1B–BMI1 RING finger heterodimer in complex with UBCH5C^30^ (Fig. 3b and Extended Data Fig. 6a). Furthermore, the RAWUL domain of BMI1 is positioned against the backside of UBCH5C, whereby the linker connecting the BMI1 RING and RAWUL domains is fully extended (Fig. 3a). This arrangement is not predicted by AlphaFold3 (AF3)^33^, which favors a compact linker and orients the RAWUL domain away from UBCH5C (Extended Data Fig. 6b). BMI1 engages the UBCH5C α1 helix through a defined interface formed together with RING1B. H2 and Y221 of BMI1 pack against RING1B to generate a composite surface that accommodates UBCH5C R15, which wedges in between RING1B R43 and BMI1 M207, forming an arginine stacking interaction (Fig. 3c). The interface is further stabilized by contacts between BMI1 Y221 and UBCH5C K8, S11 and D12. In addition, BMI1 R217 forms a hydrogen bond with UBCH5C R15, while BMI1 T214 contacts UBCH5C S11 and N7 and BMI1 R216 contacts UBCH5C M30. Together, these interactions likely account for the precise positioning of UBCH5C needed for ubiquitin transfer from UBCH5C to H2AK119.

PHC2 is also positioned near the backside of UBCH5C, suggesting a potential additional interface with the E2 enzyme. Density for PHC2 is visible up to S56; however, R54, S55 and S56 lack side-chain resolution, limiting direct assignment of specific contacts with UBCH5C (Fig. 3d). Two small unresolved densities are observed in this region, one adjacent to the UBCH5C α1 helix and another near UBCH5C S22 (Fig. 3d).

To further probe PHC2–UBCH5C proximity, we treated the nucleosome-bound PRC1–UBCH5C complex with bis(sulfosuccinimidyl) suberate (BS3) for crosslinking mass spectrometry (MS) analysis. This revealed that PHC2 K63 and K65 consistently crosslinked to UBCH5C Lys144 (Fig. 3e and Extended Data Fig. 6c). The distance between the last resolved PHC2 residue, S56, and UBCH5C K144 is compatible with these crosslinks when considering the length of the BS3 crosslinker, supporting the existence of possible PHC2–UBCH5C contacts (Fig. 3e).

### An unstructured region in PHC2 contacts UBCH5C and enhances PRC1 E3 ligase activity

To assess the functional contribution of PHC2 to PRC1 ubiquitination activity, we reconstituted a PRC1 complex in which PHC2 residues L57, L58, V59 and G60 were substituted with alanine (PRC1(PHC2_4A_)) to perturb a putative interaction of this sequence with the hydrophobic residues M38, V49, F51 and M147 of UBCH5C (Fig. 3f and Extended Data Fig. 6d,e). In time-course ubiquitination assays, the PRC1(PHC2_4A_) mutant complex exhibited more than a twofold reduction in E3 ligase activity toward H2A monoubiquitination compared to wild-type (WT) PRC1 (Fig. 3f,g). Importantly, PRC1(PHC2_4A_) bound mononucleosomes with an affinity comparable to that of the WT complex (Fig. 3h,i). These results indicate that the LLVG motif immediately C-terminal to the PHC2 HD domain promotes PRC1 E3 ligase activity, likely by stabilizing or positioning UBCH5C through direct PHC2–UBCH5C interactions, without detectably affecting PRC1 binding to the nucleosome.

Similar separation-of-function mutants in PHC2 will be needed to determine whether the proposed PHC2–UBCH5C interaction contributes to PRC1-mediated H2A monoubiquitination in vivo.

### CBX7 chromodomain interaction with H3Kc27me3

**Extended Data Fig. 7:**
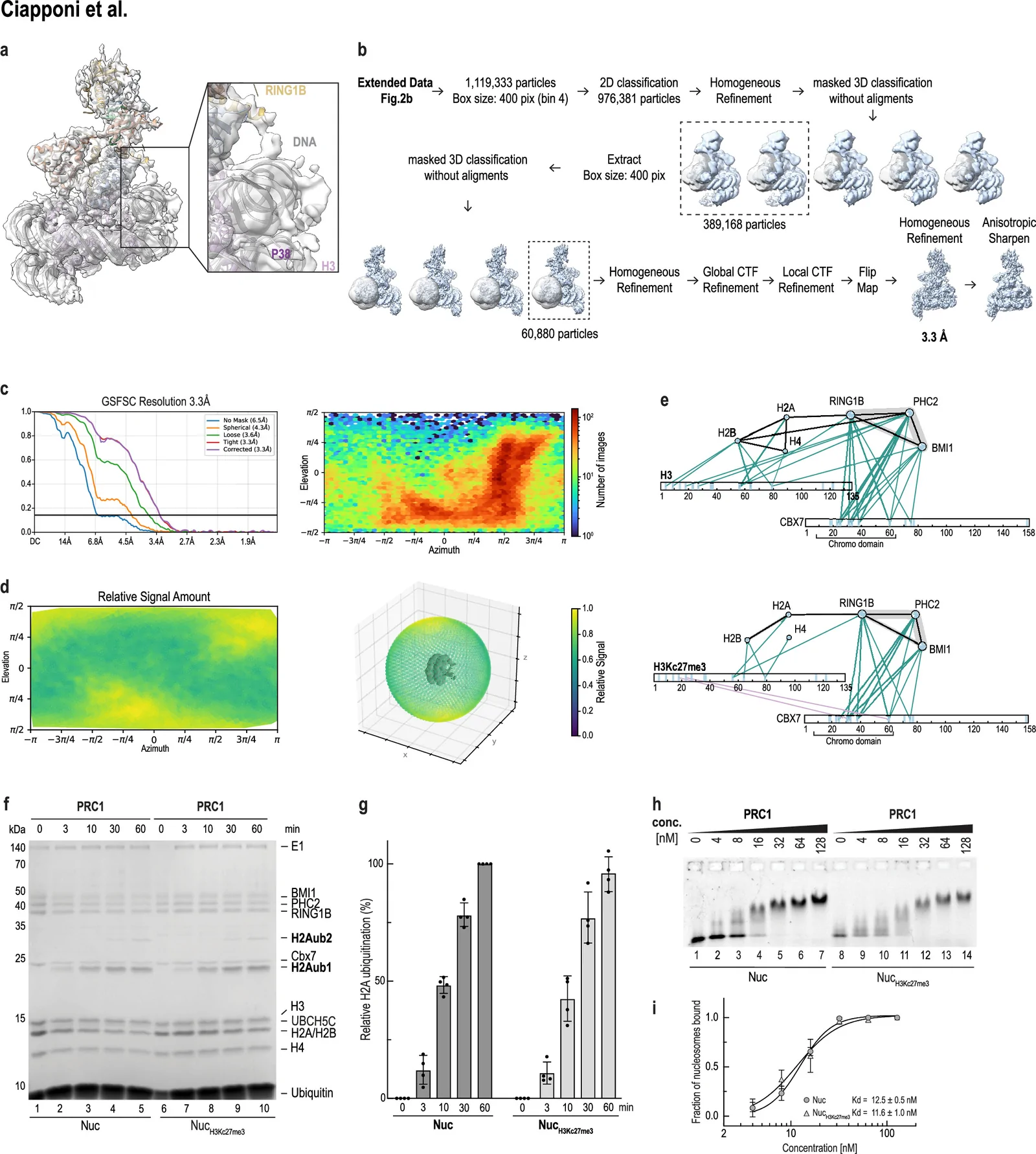
The CBX7 chromodomain engages the histone H3 N-terminus in an H3K27me3-dependent manner; however, this interaction does not measurably enhance PRC1 binding or catalytic activity. **(a)** Overview of the sharpened cryo-EM map obtained by focused refinement of a previously poorly resolved region of the map adjacent to the H3Kc27me3 tail and nucleosomal DNA. The atomic model built into the primary map is shown here for reference. A close-up highlights residual, unresolved density near the DNA; the last ordered residue of the H3 tail (Pro38) is indicated. **(b)** Cryo-EM data-processing workflow, as described in the Methods (see Extended Data Fig. 2b for initial processing steps). **(c)** Gold-standard Fourier shell correlation (GSFSC) curve (left) and particle angular distribution plot (right) from CryoSPARC. Colors indicate the number of particles per viewing direction, ranging from blue (low) to red (high). **(d)** Relative signal shown in a 2D angular distribution (azimuth–elevation) plot (left) and a 3D colored scatter plot (right), overlaid with a low-pass filtered volume. Darker regions correspond to lower relative signal, indicating under-sampled viewing directions. Plots were generated using CryoSPARC. **(e)** Cross-linking mass-spectrometry analysis of PRC1 bound to unmodified (top) or H3Kc27me3-modified (bottom) mononucleosomes. Samples were cross-linked using BS3. Identified cross-links between CBX7 and histone H3 are depicted as green lines, with cross-links between the CBX7 Chromodomain (CBX7_8-62_) and H3Kc27me3 highlighted in purple. Black lines indicate the cumulative cross-links detected among the other proteins in the sample. Lysine residues in histone H3 and CBX7 are indicated in blue. For the unmodified nucleosome sample, all cross-links detected in both technical replicates (n = 2) are shown; notably, no CBX7–H3 cross-links were observed. For the H3Kc27me3-modified nucleosome sample, only cross-links identified in at least three out of four technical replicates (n = 4) are displayed. **(f)** Time-course ubiquitination assays using the PRC1 complex on unmodified (Nuc) and H3Kc27me3-modified (Nuc_H3Kc27me3_) mononucleosomes, reveal no detectable difference in catalytic activity between the two substrates. Four independent assays were performed and quantification of the results is shown in (**g**). **(g)** Quantification of H2Aub1 signal shown in Extended Data Fig. 7f by densitometry analysis of independent experiments (n = 4). For each experiment, the H2Aub1 signal in lane 5 was normalized to 100%. Dots represent individual data points; histogram bars represent mean values +/– SD. **(h)** EMSA assessing binding of the PRC1 complex to unmodified (Nuc) and H3Kc27me3-modified (Nuc_H3Kc27me3_) mononucleosomes, revealing comparable binding to both substrates. Four independent assays were performed and are quantified in (**i**). **(i)** Quantification of the EMSA data shown in Extended Data Fig. 7h by densitometry analysis of ATTO-647 N signal from independent experiments (n = 4). Data are presented as mean values +/– SD; apparent Kd values are shown. Source data

We detected an additional, poorly resolved density near the exit of the histone H3 tail between the DNA gyres (Extended Data Fig. 7a). Focused refinement yielded a 3.3-Å map (Extended Data Fig. 7a–d) but the local resolution was insufficient for reliable atomic model building. Therefore, we considered whether this density might correspond either to an extension of the RING1B helix_116–137_ or to the CBX7 chromodomain engaging H3K27me3 (refs. ^21,22,34^). To evaluate the latter possibility, we performed crosslinking MS on PRC1 bound to unmodified (Nuc) or H3K27me3-modified (Nuc_H3Kc27me3_) mononucleosomes. Crosslinks between CBX7 and histone H3 were detected exclusively in the PRC1:Nuc_H3Kc27me3_ sample, with CBX7 K33 and K60 linked to H3 K18 and K23 (Extended Data Fig. 7e). However, when using the PRC1 holocomplex, we observed comparable H2A ubiquitination activity and nucleosome binding affinities on both unmodified and H3Kc27me3 nucleosomes (Extended Data Fig. 7f–i). Under these conditions, CBX7 chromodomain binding to H3Kc27me3, therefore, does not measurably enhance PRC1 activity.

### The enzymatic activity and compact architecture are conserved in *Drosophila* PRC1

**Fig. 4:**
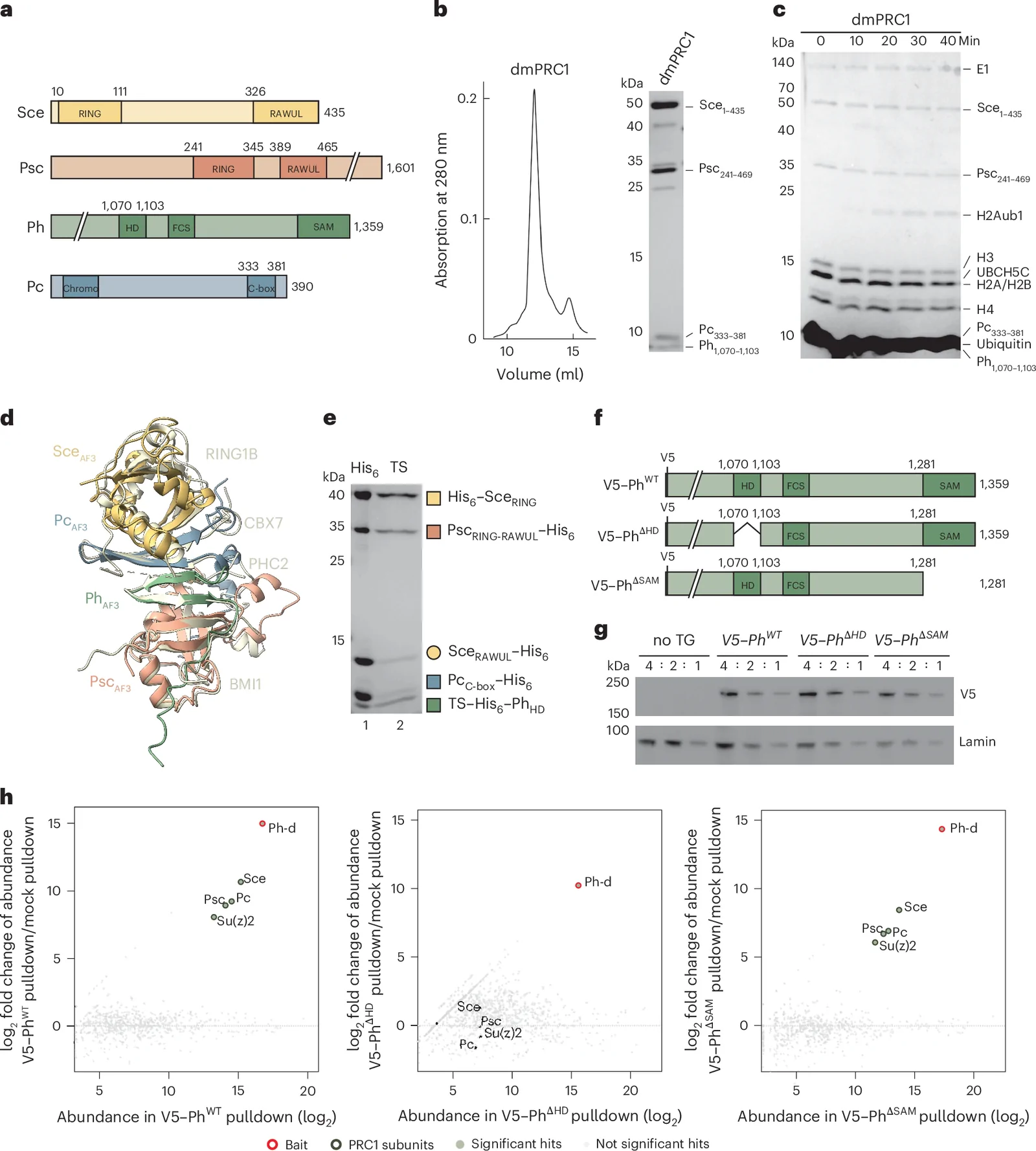
PRC1 architecture and catalytic activity is conserved in *Drosophila*. **a**, Domain organization of *Drosophila* PRC1 (dmPRC1) subunits. **b**, Gel-filtration profile and Coomassie-stained gel of dmPRC1 reconstituted with the indicated fragments of the four subunits. The complex was purified once. **c**, Time-course ubiquitination assay on mononucleosomes with the minimal dmPRC1 complex shown in **b**, monitoring H2Aub1 formation. Similar activity was observed in two independent experiments. **d**, AF3 prediction of the upper lobe of dmPRC1 superimposed with Sce_RAWUL_ (yellow), Psc_RAWUL_ (orange), Ph_HD_ (green) and Pc_C‑box_ (blue) on the upper lobe of the human PRC1 cryo‑EM structure (pale yellow). **e**, A stable dmPRC1 assembly can be reconstituted from minimal folded domains. Split Sce, comprising the RING (Sce_1__–150_) and RAWUL (Sce_326__–435_) fragments, together with Psc_RING–RAWUL_ (Psc_241__–469_), the Pc_C-box_ (Pc_333__–381_) and Ph_HD_ (Ph_1,070__–1,281_), were coexpressed in insect cells. Proteins were affinity-purified either through the His_6_ tag present on all polypeptides (lane 1) or through the TS tag on Ph_HD_ (lane 2). Eluates were resolved by PAGE and visualized by Coomassie staining. Similar results were obtained in four independent TS purifications. **f**, Schematic representation of transgene-encoded V5-tagged Ph proteins used for affinity purification (**h**) and genetic analysis (Extended Data Fig. 9 and Fig. 5). **g**, Western blot analysis on serial dilutions of total extracts from 2–16-h-old embryos expressing the indicated transgene-encoded V5-tagged Ph proteins in a WT genetic background. Membranes were probed with anti-V5 antibody and with anti-lamin as a loading control. Embryos lacking a transgene (no TG) served as negative control. Similar results were obtained in three independent (*n* = 3) western blot experiments. **h**, V5 affinity purifications from *Drosophila* transgenic embryos of the same genotypes as in **g**. Scatter plots show protein abundance versus enrichment, as determined by MS. Significant hits are displayed as large green dots, whereas nonsignificant proteins are shown as small gray dots. PRC1 subunits are highlighted with a dark outline and the V5–Ph bait proteins are indicated by a red circle. Additional details are provided in Methods. Source data

A combination of biochemical analyses and AF3 modeling indicated that *Drosophila* cPRC1, comprising Sce, Psc, Ph and Pc, adopts an architecture closely resembling that of human PRC1. First, a purified recombinant minimal *Drosophila* PRC1 complex reconstituted from full-length Sce, a Psc fragment containing the RING and RAWUL domains, the Ph HD domain and the Pc C-box (Fig. 4a,b) exhibited robust E3 ligase activity for H2AK119 monoubiquitination on reconstituted *Xenopus* mononucleosomes (Fig. 4c). Second, AF3 modeling of these subunits suggested a similar structure, as shown by the near-perfect superposition of the upper lobes of *Drosophila* and human PRC1 (Fig. 4d). Third, consistent with this model, coexpression of the Psc_RING–RAWUL_, Ph_HD_ and Pc_C-box_ fragments together with split Sce RING (Sce_1__–150_) and RAWUL (Sce_326__–435_) domains, followed by Strep affinity purification through Ph_HD_, recovered all five polypeptides (Fig. 4a,e). Together, these results demonstrate that PRC1 adopts a conserved compact architecture in *Drosophila*, with the Ph HD domain functioning as a central scaffolding element.

### The Ph HD domain is critical for Ph incorporation into PRC1 in vivo

We next examined the role of the Ph HD domain in PRC1 assembly in *Drosophila* embryos. Using an affinity purification approach, we compared the ability of transgene-encoded V5–Ph^WT^ and a mutant lacking the HD domain (V5–Ph^ΔHD^) to assemble into PRC1 complexes in vivo (Fig. 4f). As an additional control, we analyzed a Ph variant lacking the SAM domain (V5–Ph^ΔSAM^) (Fig. 4f). Western blot analysis of embryonic extracts confirmed that all three V5-tagged proteins were expressed at comparable levels (Fig. 4g). V5 affinity purification from embryos expressing V5–Ph^WT^ or V5–Ph^ΔSAM^ resulted in robust coenrichment of the PRC1 core subunits Sce, Pc and Psc or its paralog Su(z)2 (Fig. 4h). In contrast, V5 affinity purification from V5–Ph^ΔHD^ embryos recovered only the mutant Ph protein, with no detectable copurification of other PRC1 subunits (Fig. 4h). Together, these results demonstrate that the Ph HD domain is indispensable for Ph incorporation into PRC1 in vivo, whereas the SAM domain is dispensable for complex assembly.

### The Ph SAM domain mediates cPRC1 targeting and H2AK118 monoubiquitination at canonical Polycomb targets

In *Drosophila*, PRC1 binding to chromatin is highly localized at Polycomb response elements (PREs) in Polycomb target genes and H2AK118 monoubiquitination deposited by cPRC1 is similarly restricted to these loci^11^. PRC1 recruitment to PREs is thought to involve direct physical interactions with the DNA-binding PhoRC complex^28,35,36^. Structural insight into this process has, however, remained limited to SAM-domain-mediated interactions linking the PhoRC subunit Sfmbt to Scm and, in turn, to the SAM domain of Ph^27,28^. These findings led to a model in which the Ph SAM domain contributes to the recruitment of cPRC1 to PREs^28^, consistent with genetic evidence showing that a *Ph*^*ΔSAM*^ transgene fails to rescue the severe phenotype of *ph*^*KO*^ mutants^37^.

**Extended Data Fig. 8:**
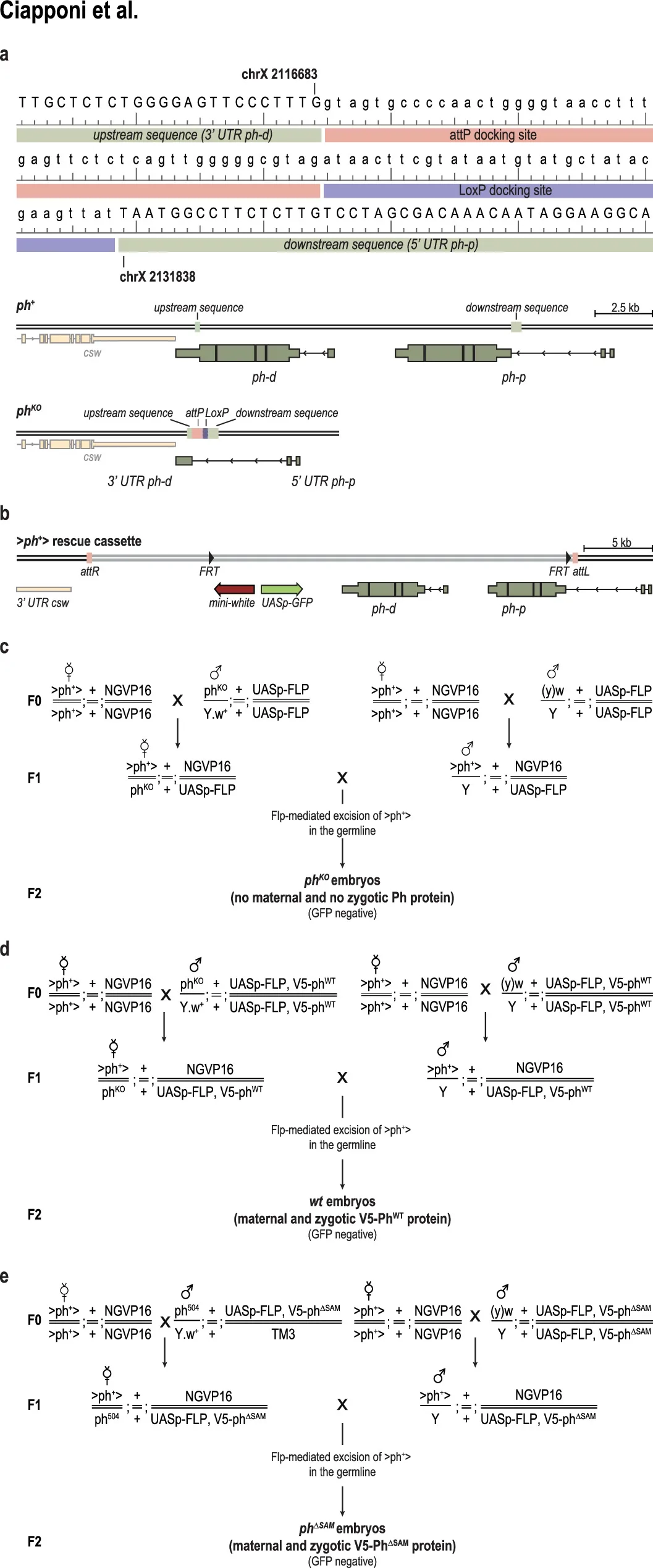
Strategy for generating *ph* mutant embryos. **(a)** Top: genomic sequence (chrX 2116660-2131879) surrounding the deleted region in the *ph*^*KO*^ allele. The genomic region from chrX 2116684 to 2131837 was replaced by an *attP* docking site and a *LoxP* site, as indicated. Bottom: schematic representation of the genomic region from chrX 2111803 to chrX 2138716 for the unmodified *ph* locus (*ph*^*+*^) or the *ph*^*KO*^ allele. The location of the sequences highlighted in the upper panel is indicated. **(b)** Schematic representation of the >*ph*^*+*^*>* rescue cassette (grey double bar, see Methods for details), integrated in the *attP* docking site of the *ph*^*KO*^ allele. The genomic integration of the rescue cassette via *attP*-*attB* recombination resulted in the formation of *attR* and *attL* sequences, as indicated. Upon integration, the transcriptional units of both *ph-d* and *ph-p* paralogs are restored, and animals hemizygous or homozygous for the engineered locus are fully viable and fertile. The black arrow heads represent FRT sites; FLP-mediated excision results in the deletion of *ph-d* and *ph-p* coding regions, and the *GFP* and *mini-white* marker genes. *UASp-GFP: 14xUASGal4 – P-element promoter – nuclear GFP marker – K10 3’UTR*. **(c-e)** Crosses performed to generate *ph*^*KO*^ embryos (**c**), *ph*^*KO*^ embryos carrying *V5-Ph*^*WT*^ (*wt* embryos) (**d**) and *ph*^*KO*^ embryos carrying *V5-Ph*^*ΔSAM*^ (*ph*^*∆SAM*^ embryos) (**e**) (see Fig. 4f, g and Methods for details about the *V5-Ph* transgenes). NGVP16: *nanos-Gal4-VP16*. UASp-FLP: FLP recombinase expressed under the control of *UASGal4 – P-element promoter*^37^. Y.w+ corresponds to an interchromosomal duplication of the cytological regions 2D2 to 3D3, including the *ph*^+^ and *white*^*+*^ gene loci, onto Y.

**Extended Data Fig. 9:**
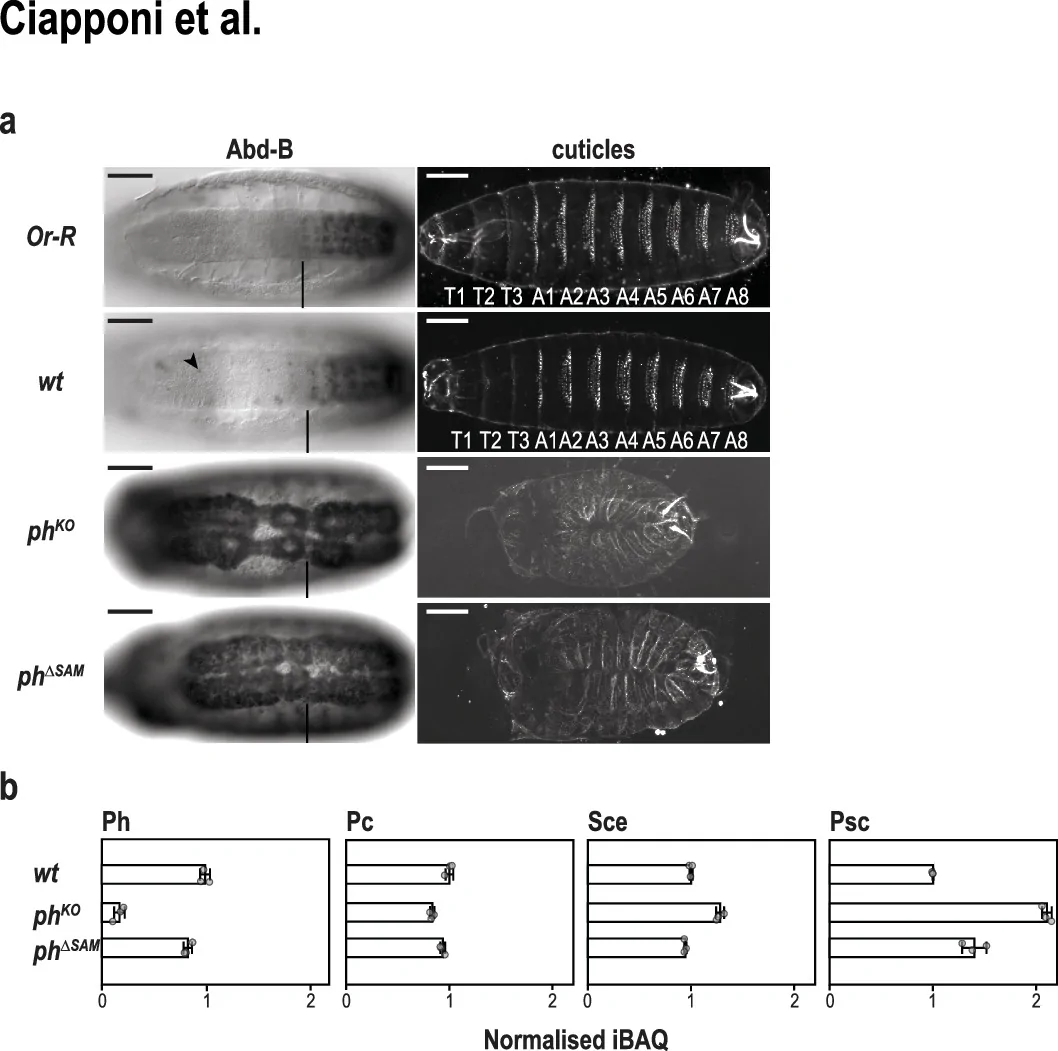
*ph*^*∆SAM*^ embryos exhibit severe morphological abnormalities and defects in Polycomb target gene repression that are indistinguishable from those observed in *ph*^*KO*^ embryos. **(a)** Stage 15 or 16 embryos stained with anti-Abd-B antibody (left) and ventral views of embryonic cuticles (right) of the indicated genotypes. Vertical bars mark the normal anterior boundary of Abd-B expression at the anterior limit of parasegment (ps) 10 in wild-type Oregon-R (*Or-R*) embryos. In *ph*^*KO*^ embryos, the product of the Polycomb target gene *Abd-B* is broadly misexpressed, accompanied by pronounced major morphological abnormalities that are also evident in the embryonic cuticle. In *ph*^*KO*^ embryos carrying one copy of the *V5-Ph*^*WT*^ transgene (referred to here and in the main text as *wt*), Abd-B repression and normal cuticle morphology are largely restored. A small number of Abd-B-positive cells anterior to ps 10 remain (black arrowhead), indicating incomplete rescue by one copy of the *V5-Ph*^*WT*^ transgene. *ph*^*∆SAM*^ embryos (that is *ph*^*KO*^ embryos carrying one copy of the *V5-Ph*^*∆SAM*^ transgene) are indistinguishable from *ph*^*KO*^ embryos: Abd-B is broadly misexpressed (black arrowheads) and the cuticles display similarly severe morphological defects. In all three mutant genotypes, these phenotypes were observed in 100% of the animals analyzed (N > 15 for all genotypes). Scale bars are shown (white = 100 µm; black = 50 µm). **(b)** Quantitative mass-spectrometry analysis of proteins in nuclear extracts from 16-20 hours embryos of the same genotypes as in (**a**). The abundance of the PRC1 subunits Pc, Sce, and Psc is comparable across all three genotypes. The levels of transgene-encoded V5-Ph^WT^ and V5-Ph^∆SAM^ are likewise similar and exceed those of the residual maternally-deposited endogenous Ph protein that persists in these embryos. For each of the four proteins, the mean iBAQ value in the wt embryo sample was set to 1, and the iBAQ values in each individual sample were normalized to this value. In each genotype, dots represent the normalized iBAQ values from three biological replicates, bars represent the mean ± SD.

To test this model at the molecular level, we analyzed PRC1 binding and H2Aub1 distribution in embryos expressing Ph^ΔSAM^ in place of WT Ph. We introduced *V5–Ph*^*ΔSAM*^ or, as a control, *V5–Ph*^*WT*^ transgenes into embryos homozygous or hemizygous for a newly generated *ph*^*KO*^ deletion allele that removes the coding regions of the closely linked paralogs Ph-p and Ph-d (Extended Data Fig. 8). *ph*^*KO*^ embryos carrying *V5–Ph*^*ΔSAM*^ (*ph*^*∆SAM*^ mutants) exhibited severe morphological defects and Polycomb target gene misregulation indistinguishable from those of *ph*^*KO*^ mutants, whereas these phenotypes were largely rescued by the *V5–Ph*^*WT*^ transgene, hereafter referred to as the *wt* control (Extended Data Fig. 9a). Quantitative MS further showed that levels of the remaining PRC1 core subunits were comparable across *wt*, *ph*^*KO*^ and *ph*^*∆SAM*^ embryos (Extended Data Fig. 9b), permitting direct comparison of PRC1 genomic localization and H2Aub1 profiles among the three genotypes.

**Fig. 5:**
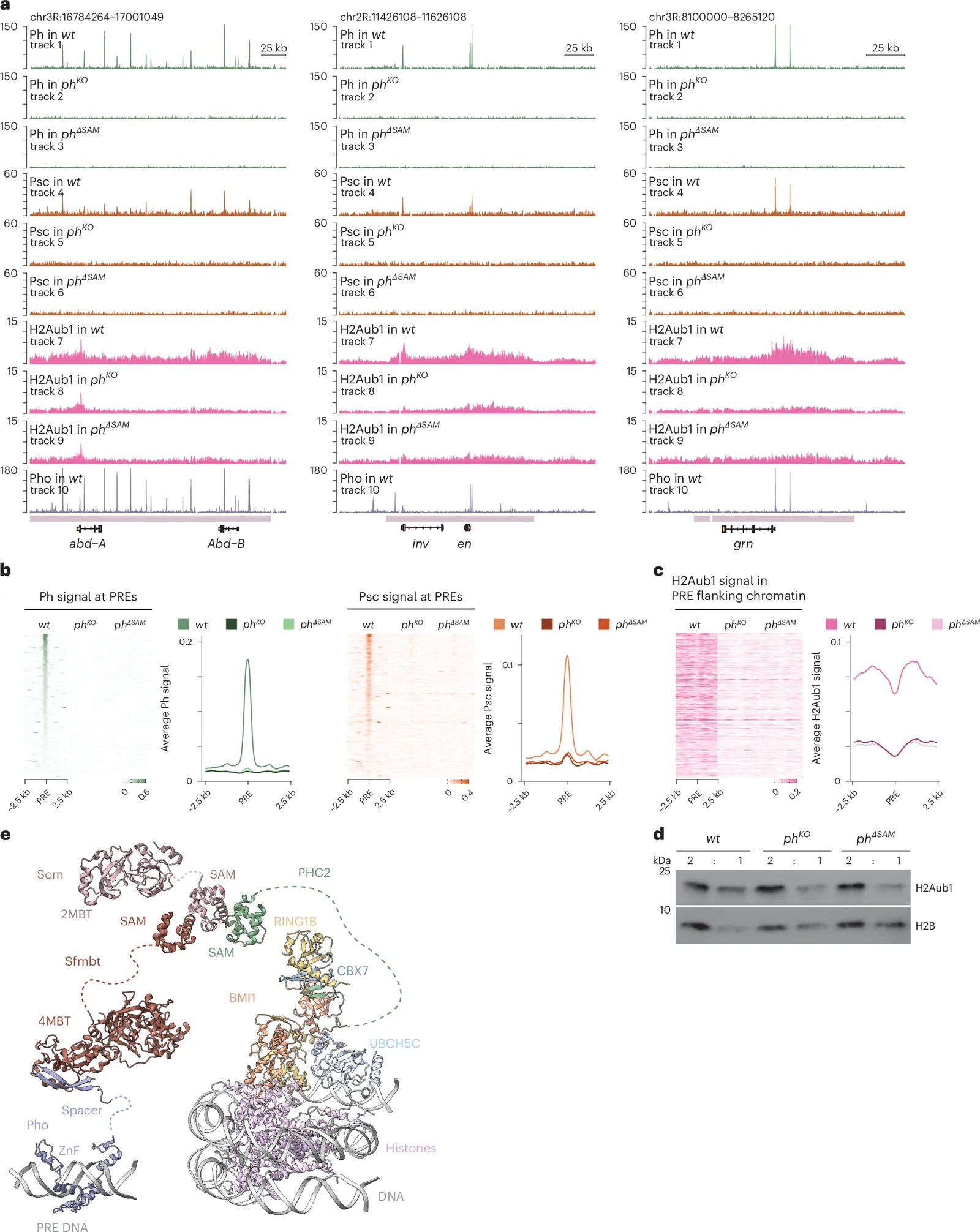
Ph^SAM^-mediated recruitment to PREs is critical for targeted H2A monoubiquitination by PRC1 in early embryos. **a**, V5–Ph (tracks 1–3), Psc (tracks 4–6), H2Aub1 (tracks 7–9) and Pho (track 10) ChIP-seq profiles in 1–3-h-old embryos of the indicated genotypes, generated as depicted in Extended Data Fig. 8. The purple boxes below track 10 denote chromatin domains enriched for H2Aub1 at this developmental stage. **b**, Heat map (left) and average profile (right) depicting V5–Ph and Psc signals in 5-kb windows centered on PREs in 1–3-h-old embryos of the three indicated genotypes. Ranking is based on the V5–Ph signal in *wt*. **c**, Same as **b**, but for H2Aub1. **d**, Western blot analysis of H2Aub1 bulk levels on twofold dilutions of nuclear extracts from 1–3-h-old *wt*, *ph*^*KO*^ and *ph*^*∆SAM*^ embryos. The same western blot membrane was probed with anti-H2Aub1 and, as a control, anti-H2B antibody. Similar results were obtained in three independent western blot experiments. **e**, Molecular models of interactions for PRE tethering of PRC1 by PhoRC. PhoRC, composed of Pho and Sfmbt, binds PRE DNA through the zinc finger (ZnF; purple) domain of Pho, which recognizes the GCCAT motif (gray). As a structural model for this interaction, we show the ZnF domain of the human Pho ortholog YY1—identical in all DNA-contacting residues—bound to GCCAT (PDB 1UBD)^50^. The spacer region of Pho associates with the 4MBT domain of Sfmbt (dark red; PDB 4C5E)^51^. The Sfmbt SAM domain engages the SAM domain of Scm (PDB 5J8Y)^28^, which in turn interacts with the SAM domain of Ph (PDB 1PK1)^27^. This ordered head-to-tail assembly of SAM domains establishes a structural bridge linking PRE-bound PhoRC to cPRC1, thereby positioning the complex for nucleosome engagement and UBCH5C recruitment in the surrounding chromatin. As a structural model, we depict the nucleosome-bound human PRC1–UBCH5C complex determined in this study (subunits colored as in Fig. 1). The 2MBT domain of Scm (PDB 2R57) and the 4MBT domain of Sfmbt recognize monomethylated and dimethylated lysines on histones H3 and H4 (refs. ^43,52^) and may provide additional contacts with nucleosomes within target gene chromatin. Source data

Chromatin immunoprecipitation (ChIP) assays in 1–3-h-old *wt* embryos detected defined localized binding of V5–Ph^WT^ and Psc at PREs of Polycomb target genes (Fig. 5a,b, tracks 1 and 4). In contrast, binding of both V5–Ph^∆SAM^ and Psc was undetectable at PREs in *ph*^*∆SAM*^ embryos (Fig. 5a,b, tracks 3 and 6). Thus, although V5–Ph^ΔSAM^ retains the capacity to assemble into PRC1 (Fig. 4h), it fails to recruit the complex to PREs.

**Extended Data Fig. 10:**
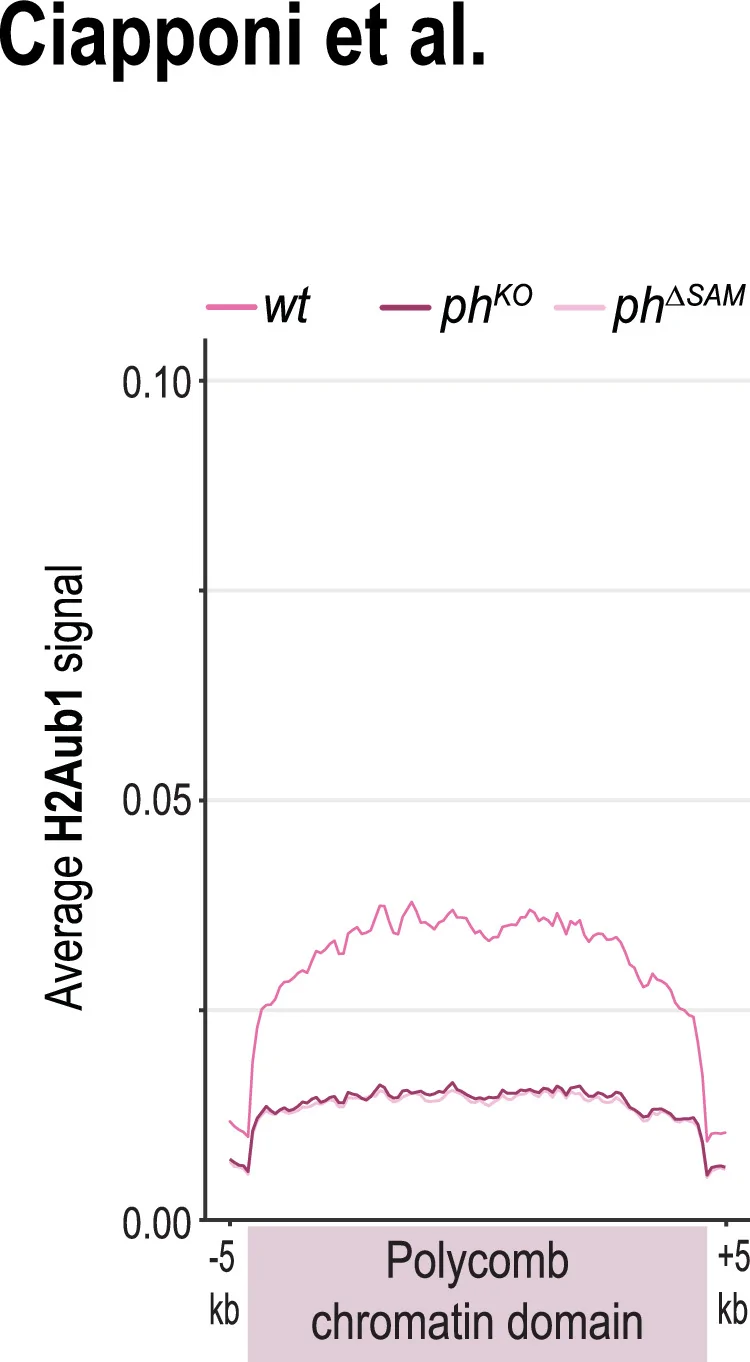
H2Aub1 levels across Polycomb chromatin domains are reduced in *ph*^*KO*^ and *ph*^*ΔSAM*^ embryos. Average H2Aub1 profiles across Polycomb chromatin domains in 1-3 hours old embryos of the three indicated genotypes. The profiles include the 5 kb genomic regions flanking each Polycomb chromatin domain.

This defect in tethering is mirrored by a loss of PRC1 catalytic activity at target loci. In 1–3-h-old *wt* embryos, H2Aub1 is enriched in broad chromatin domains spanning Polycomb target genes^38,39^ (Fig. 5a, track 7, and Extended Data Fig. 10). In both *ph*^*∆SAM*^ and *ph*^*KO*^ embryos, however, H2Aub1 levels are markedly reduced across these regions, especially in the vicinity of PREs (Fig. 5a,c, tracks 8 and 9, and Extended Data Fig. 10). Importantly, this reduction is restricted to Polycomb targets, whereas the bulk, genome-wide H2Aub1 pool—generated by variant PRC1 complexes^11^—remains largely unaffected (Fig. 5d).

Together, these data demonstrate that, in *Drosophila*, recruitment of cPRC1 to PREs through the SAM domain of Ph is essential for locus-specific H2AK118 monoubiquitination at Polycomb target genes.

## Discussion

This study investigated the structure, enzymatic activity and genomic targeting of PRC1. Below, we discuss the principal findings related to each of these fundamental aspects in turn.

### Principles of PRC1 assembly

The structural analysis of human cPRC1 reveals that its core subunits assemble into a compact, highly integrated complex. This architecture clarifies how PRC1 diversification gives rise to cPRC1.2 and cPRC1.4, as well as variant PRC1.1, PRC1.3, PRC1.5 and PRC1.6 assemblies.

In mammals, diversification begins with dimerization of the catalytic subunit RING1B or its paralog RING1A, with one of six PCGF paralogs (PCGF1, PCGF2, PCGF3, BMI1, PCGF5 and PCGF6) through their RING finger domains. The PCGF RAWUL domain then recruits complex subtype-specific subunits: BCOR in PRC1.1 (PCGF1), PHC1–PHC3 in PRC1.2 and PRC1.4 (PCGF2/BMI1) and AUTS2 in PRC1.3 and PRC1.5 (PCGF3/PCGF5)^4,5,25,32,40,41^. In parallel, the RAWUL domain of RING1B (or RING1A) engages in mutually exclusive interactions with either the C-box of CBX proteins, in the case of cPRC1.2 and cPRC1.4, or the C-terminal region of RYBP, in the case of variant PRC1 complexes. Our PRC1.4 structure reveals that the CBX7 C-box, while bound to the RING1B RAWUL domain, simultaneously contacts the HD domain of PHC2 through a distinct interface, thereby bridging RING1B and PHC2 (Fig. 2). This bipartite interaction likely stabilizes CBX7 within cPRC1 and provides a structural explanation for the preferential incorporation of CBX proteins over RYBP in canonical assemblies.

### Chromatin binding by cPRC1

Our structural analysis shows that the human PRC1 holocomplex engages the mononucleosome through a defined interaction in which the RING1B–BMI1 RING finger heterodimer binds the nucleosomal acidic patch (Fig. 1). This canonical binding mode is preserved in the absence of UBCH5C, with only the upper lobe exhibiting increased conformational heterogeneity (Extended Data Fig. 5). In addition, our analyses indicate that the CBX7 chromodomain can contact the H3 N-terminal tail, possibly through H3K27me3 engagement but this interaction did not measurably affect nucleosome binding affinity or enzymatic activity under our experimental conditions (Extended Data Fig. 7).

It is conceivable that incorporation of alternative CBX paralogs—such as CBX2, whose extended, positively charged intrinsically disordered region exhibits DNA-binding and chromatin-compacting activity in vitro^15,42^—as well as inclusion of the accessory factor Scm, which binds methylated histone lysines^27,28,43^, could promote additional chromatin interaction modes. Likewise, the FCS domain of the cPRC1 subunit PHC1 has been reported to bind both RNA and DNA^44^. More generally, engagement with chromatin fibers in vivo, where nucleosome topology and internucleosomal contacts shape chromatin architecture, may further expand PRC1 binding interfaces beyond the acidic patch interaction observed on mononucleosomes.

### Enzymatic activity of cPRC1

In late-stage *Drosophila* embryos and in embryonic stem cells, the majority of monoubiquitinated H2A—both genome-wide and at Polycomb target genes—is generated by variant PRC1 complexes, with cPRC1 contributing only minimally^9,11^. Notably, however, the H2Aub1 signal that persists upon genetic ablation of all variant PRC1 forms (that is, in *Drosophila l(3)73Ah*^*0*^ mutants or embryonic stem cells lacking PCGF1, PCGF3, PCGF5 and PCGF6) is produced by cPRC1 and is highly restricted to canonical Polycomb target genes^9,11^.

Here, we show that, during the initial establishment of H2Aub1 chromatin domains in *Drosophila* blastoderm embryos, cPRC1 has a markedly more prominent role, contributing substantially to H2AK118 monoubiquitination at Polycomb target genes (Fig. 5). Thus, the relative contributions of cPRC1 and variant PRC1 complexes to H2Aub1 domain formation are developmentally regulated and strongly context dependent.

### Ph as a central determinant of assembly, enzymatic activity and genomic targeting of cPRC1

A central conclusion of our structural and functional analyses is that the cPRC1-specific subunit PHC2/Ph coordinates complex assembly (Figs. 2 and 4), catalytic efficiency (Fig. 3) and genomic targeting of cPRC1 (Fig. 5). These distinct functions reside in separable regions of the protein. The HD domain is sufficient for stable incorporation into the core complex, while its C-terminal extension enhances E3 ligase activity. Whether the PHC2/Ph–UBCH5C interaction contributes to PRC1-mediated H2A monoubiquitination in vivo remains to be determined by analyzing separation-of-function mutants that selectively disrupt this interaction. By contrast, the SAM domain, which mediates genomic targeting, does not participate in core assembly but instead engages the SAM domain of Scm, thereby linking cPRC1 to PRE-bound PhoRC (Fig. 5e).

In *Drosophila*, PRC1 complexes lacking the Ph SAM domain fail to associate with PREs at Polycomb target genes and exhibit a pronounced reduction in H2AK118ub1 across these loci (Fig. 5). This supports a model in which tethering of cPRC1 to PREs through PhoRC is required to position the complex for productive nucleosome engagement through acidic patch binding and UBCH5C recruitment (Fig. 5e). We propose that this mechanism initially generates H2AK118ub1-modified nucleosomes in chromatin flanking PREs, which may serve as binding platforms for the RYBP subunit of variant PRC1 complexes^45–47^. In this scenario, cPRC1-generated H2Aub1-marked nucleosomes across Polycomb target genes could provide a molecular foundation for the establishment of more densely H2AK118ub1-modified domains through the RYBP-mediated read–write mechanism of variant PRC1 complexes, for which no dedicated targeting mechanisms have yet been identified in *Drosophila*.

## Methods

### Human PRC1 expression constructs

The full-length coding sequences of human RING1B (UniProt Q99496), BMI1 (UniProt P35226), CBX7 (UniProt B0QYP2) and PHC2 (UniProt Q8IXK0-2) were cloned into separate pFastBac vectors for expression in insect cells. RING1B was untagged, BMI1 was expressed with a C-terminal HRV3C-cleavable His_6_ tag, CBX7 with an N-terminal HRV3C-cleavable His_6_ tag and twin Strep (TS) tags and PHC2 with an N-terminal HRV3C-cleavable His_6_. The PHC2 SAM domain was mutated to contain L307E;L311E substitutions to disrupt SAM-domain-mediated oligomerization.

The PHC2_4A_ (L57A;L58A;V59A;N60A) mutant expression construct was prepared using standard site-directed mutagenesis.

For minimal PRC1 assemblies with RING1B_1__–165_ (N-terminal His_6_–GST), RING1B_225__–333_ (C-terminal His_6_), BMI1_1__–232_ (C-terminal His_6_), CBX7_111__–156_ (C-terminal His_6_–EPEA affinity tag (referred to as C-tag)) and PHC2_28__–69_ (N-terminal His_6_–TS) fragments were cloned into separate pLIB vectors by Gibson assembly.

Bacmids of all the PRC1 subunit constructs were obtained using the DH10EMBacY kit (Geneva Biotech).

### *Drosophila* PRC1 expression constructs

*Drosophila* PRC1 complexes were reconstituted from fragments of the core subunits Sce (UniProt Q9VB08), Psc (UniProt P35820), Ph (UniProt Q9NF31) and Pc (UniProt P26017) for expression in *Escherichia* *coli* strain BL21(DE3) pLysS. For ubiquitination assays, full-length Sce was cloned into the pEC-A *E*. *coli* expression vector with an N-terminal His_6_–GST tag followed by an HRV3C cleavage site. Psc_241–469_, Ph_1,070–1,103_ and Pc_333–381_ were cloned into a polycistronic pEC-K vector. In this construct, Psc carried an N-terminal SUMO tag and a C-terminal His_6_ tag, Ph an N-terminal TS tag and Pc a C-terminal His_6_ tag. All affinity tags were removable by HRV3C protease cleavage.

For reconstitution of minimal *Drosophila* PRC1, Sce_1–150_ (N-terminal His_6_–GST), Sce_326__–435_ (C-terminal His_6_), Psc_214–469_ (C-terminal His_6_), Pc_333–381_ (C-terminal His_6_–CTAG) and Ph_1,070–1,103_ (N-terminal His_6_–TS) were cloned individually into pLIB vectors for expression in insect cells. Bacmids were generated using the DH10EMBacY system (Geneva Biotech).

### Protein expression and purification

PRC1 subunits (RING1B, BMI1, PHC2 and CBX7) were coexpressed in *Trichoplusia* *ni* HighFive insect cells (Invitrogen, B85502, BTI-Tn-5B1-4, RRID:CVCL_C190) as described previously^53^. Cells were harvested and lysed by Dounce homogenization. PRC1 complexes were purified by Ni-NTA affinity chromatography, followed by Strep-Tactin affinity chromatography. Affinity tags were removed by HRV3C protease digestion (PreScission protease; MPI of Biochemistry Protein Core Facility), followed by overnight dialysis against 25 mM Tris-HCl pH 7.5, 250 mM NaCl and 10% glycerol. The sample was further purified by cation-exchange chromatography and size-exclusion chromatography in 25 mM Tris-HCl pH 7.0, 150 mM NaCl, 10% glycerol and 2 mM DTT.

The full-length RING1B–BMI1 complex was expressed and purified as described above, except that only His-tag affinity purification was used. The purity of all the protein complexes after gel filtrations was checked on polyacrylamide gels.

Recombinant human UBCH5C and the RING1B_1–159_–BMI1_1–109_ complex were expressed and purified as described^23^. In the case of the RING1B_1–159_–BMI1_1–109_ complex, we found that the RING1B protein contained a C-terminal truncation of 29 aa, such that the purified dimer comprised RING1B_1–130_ and BMI1_1–109_.

The *Drosophila* PRC1 complex used for ubiquitination assays was expressed in *E*. *coli* Rosetta (DE3) pLysS cells through coexpression of pEC-A_Sce_FL_ with pEC-K_Psc_241–469_, Ph_1,070–1,103_ and Pc_333–381_. Transformed cells were grown at 37 °C in 10× TB to approximately an optical density at 600 nm of 1.0 and expression was then induced with 0.5 mM IPTG overnight (18 °C). Collected cells were lysed by sonication and the *Drosophila* complex was purified as described for human PRC1.

### Affinity purification of minimal PRC1

Minimal human PRC1, composed of RING1B_1–165_, RING1B_225–333_, BMI1_1–232_, CBX7_111–156_ and PHC2_28–69_, was reconstituted by coexpression in insect cells as descibed above. Cell pellets from 50-ml cultures were lysed in 1.5 ml of 25 mM Tris-HCl pH 7.5, 150 ml NaCl, 1 mM MgCl_2_, 1 mM DTT by centrifugation (15,800*g*, 30 min, 4 °C). Next, 800 μl of clarified lysate was incubated with 20 μl of Ni-NTA agarose beads (Qiagen, 30210) for 30 min at 4 °C. After three washes, bound proteins were eluted in 50 μl of 25 mM Tris-HCl pH 7.5, 250 mM NaCl and 250 mM imidazole. In parallel, the other 800 μl of lysate was incubated with 20 μl of C-tag agarose beads (Thermo Scientific; CaptureSelec C-tagXL Affinity Matrix, 191307010) under the same conditions and eluted in 50 μl of 25 mM Tris-HCl pH 7.5, 250 mM NaCl and 2 M MgCl_2_. The same procedure was applied to minimal human PRC1 complexes in the case where only RING1B_1–165_, RING1B_225–333_, BMI1_1–232_ and CBX7_111–156_ were coexpressed. Eluates were analyzed on 16% SDS–PAGE gels followed by Coomassie staining.

Analogous affinity purifications were performed with the minimal *Drosophila* PRC1 complex (Sce_1–150_, Sce_326–435_, Psc_214–469_, Pc_333–381_ and Ph_1,070–1,103_). In this case, one half of the lysate was incubated with Ni-NTA beads and eluted as described above, while the other half was incubated with Strep-Tactin magnetic beads (IBA Lifesciences, 2-5090-002) and eluted in 50 μl of 25 mM Tris-HCl pH 7.5, 250 mM NaCl and 50 mM biotin.

### Reconstitution of mononucleosomes

DNA templates for mononucleosome assembly contained a single copy of the 147-bp Widom 601 nucleosome-positioning sequence^54^. The 215-bp DNA template used in this study was generated by PCR using the primers 5′-ATATCTCGGGCTTATGTGATGGACCCTATACGCGGCCGCC-3′ (forward) and 5′-ATATCCCGAGTCGCTGTTCAATACTAGC-3′ (reverse). For cryo-EM and electrophoretic mobility shift assay (EMSA) analysis, primers were modified with a 5′ biotin or 5′ ATTO647N label, respectively. PCR products were purified by MonoQ anion-exchange chromatography (GE Healthcare), ethanol-precipitated and resuspended in the high-salt buffer used for assembly with histone octamers.

*Xenopus* *laevis* histones were purchased from The Histone Source at Colorado State University. For histone octamers, equimolar amounts of histones H2A, H2B, H4 and H3 or H3K27me3_C110A_ (in the text referred to as H3Kc27me3) were mixed and assembled into octamers in high-salt buffer containing 10 mM Tris-HCl pH 7.5, 2 M NaCl, 1 mM EDTA and 5 mM β-mercaptoethanol. Subsequent SEC was performed to separate octamers from H3/H4 tetramers and H2A/H2B dimers^55^. Optimized ratios of octamer to DNA were mixed and nucleosomes were reconstituted by gradient and stepwise dialysis against low-salt buffers to a final buffer containing 10 mM Tris-HCl pH 7.5, 30 mM NaCl and 2 mM DTT. Reconstituted nucleosomes were analyzed on native agarose gels.

### In vitro H2AK119 ubiquitination assays

Ubiquitination assays were performed as previously described^56^. Briefly, 350 nM nucleosomes were incubated in reactions containing E3 ligase (168 nM), UBE1 (35 nM), UBCH5C (250 nM), ubiquitin (19 μM) and ATP (5 mM) in 50 mM Tris-HCl pH 7.5, 10 mM MgCl_2_, 1 mM ZnCl_2_ and 0.5 mM DTT. Reactions were incubated at 30 °C for the indicated times before quenching with SDS sample buffer. H2A ubiquitination was assessed by separation of reaction products on 16% SDS–PAGE gels followed by Coomassie staining. For quantification, ubiquitination reactions were performed at least in triplicate (*n* for each experiment is indicated in the figure legends) and analyzed by densitometry. For each lane, the signals of the H2Aub1 band and, when present, the H2Aub2 band were summed up, with the H2Aub2 band signal multiplied by factor two, reflecting that two ubiquitin molecules had been ligated to H2A. The resulting values were background-subtracted using Image Lab software (version 6.1) and normalized to the H4 band. For each ubiquitination reaction, the highest value was set to 100% and used as a reference; the remaining time points were normalized to this reference value. Graphs were generated using Prism 9. Human UBE1 and ubiquitin were purchased from Boston Biochem.

### Cryo-EM sample preparation and data collection

Nucleosome-bound PRC1 complex was prepared by incubating 150 nM 5′-biotinylated nucleosomes with 4.6 µM PRC1 for 1 h at 4 °C in buffer containing 25 mM Tris-HCl pH 7.5, 250 mM NaCl and 10% (v/v) glycerol. The nucleosome-bound PRC1–UBCH5C complex was prepared under the same conditions using equimolar concentrations of PRC1, UBCH5C and ubiquitin (1.6 µM each) with 150 nM nucleosomes. PRC1 was used in molar excess to maximize nucleosome occupancy; unbound proteins were removed during blotting and did not affect micrograph quality.

Quantifoil R2/2 gold grids coated with a streptavidin monolayer were prepared as previously described^57,58^. Grids were rehydrated with water for 10 min, incubated with 5 µl of sample for 5 min to allow binding of the biotinylated nucleosomes to the streptavidin monolayer and blotted to remove unbound material. Grids were then mounted in a Mark IV Vitrobot (FEI) set to 4 °C and 96% relative humidity. Next, 3 µl of streptavidin wash buffer (25 mM Tris-HCl pH 7.5, 40 mM KCl, 1 mM MgCl_2_, 1 mM TCEP, 4% trehalose, 0.04% β-OG and 0.01% NP-40) was applied directly to the grid before blotting from the streptavidin-coated side for 3 s (blot force 4), followed by vitrification in liquid ethane.

Data were acquired on a Titan Krios transmission EM instrument (FEI) operated at 300 kV and equipped with a Gatan K3 direct electron detector in counting mode. Automated collection was performed with SerialEM^59^. For the nucleosome-bound PRC1 dataset, 26,744 videos were recorded at a nominal magnification of ×105,000, corresponding to a calibrated pixel size of 0.8512 Å. Each exposure delivered a total dose of 55.8 e^−^ per Å^2^ over 30 frames, with target defocus values ranging from –0.5 to –2.0 µm. For the nucleosome-bound PRC1–UBCH5C complex dataset, 23,558 videos were acquired under the same microscope settings, with a total dose of 60.45 e^−^ per Å^2^ fractionated into 35 frames and a defocus range of –0.6 to –2.2 µm.

### Cryo-EM data processing

Datasets were processed with cryoSPARC (version 4.7.0)^60^. The video frames were aligned, dose-weighted and corrected for beam-induced motion using patch motion correction^60^. The 2D streptavidin lattice signal was removed from motion-corrected micrographs using digital Fourier filtering, as described previously^57^ (Extended Data Figs. 2a and 5a). Resulting micrographs were imported into cryoSPARC.

For the nucleosome-bound PRC1–UBCH5C complex dataset, contrast transfer function (CTF) parameters were estimated and, on the basis of CTF quality and estimated resolution, 20,900 micrographs were selected for further analysis (Extended Data Fig. 2b). Given that streptavidin grids have a comparatively high background signal, we applied a low threshold to pick as many particles as possible. An initial subset of micrographs was subjected to blob-based picking, yielding 371,303 particles that were extracted with a box size of 360 × 360 pixels binned by a factor of 4 and subjected to 2D classification. Classes showing nucleosomes with additional density on one or both faces of the nucleosome disc (Extended Data Fig. 2b,c) were used to train Topaz^61^. Following Topaz training and picking, particles were extracted using Topaz Extract (radius: 22 pixels) and four additional rounds of 2D classification were performed to obtain high-quality class averages. These classes were used to generate three ab initio models and also as templates for a second round of Topaz training now on all micrographs. Using this trained model, 3,842,340 particles were picked and extracted with a box size of 360 × 360 pixels binned by a factor of 4. These particles were subjected to an initial heterogeneous refinement using the three ab initio volumes as reference models. Iterative heterogeneous refinements were then performed using particles from the best-resolved class to remove false picks and empty nucleosomes (Extended Data Fig. 2b). The selected particles yielded an initial reconstruction with density preferentially resolved on only one nucleosome face. This is likely because of the flexibility of the PRC1 nucleosome complex, which favors alignment toward one dominant orientation and leads to reduced resolution of the opposing density. After convergence, 1,119,333 particles from the best class were reextracted with a larger box size (400 × 400 pixels, binned by a factor of 4) and subjected to one round of 2D classification. To remove the last residual empty nucleosomes, 3D classification without alignment was performed using a focused mask around the nucleosome-associated density and iterated until convergence (Extended Data Fig. 2b). This resulted in 411,972 particles, which were refined by homogeneous refinement. Particles were then unbinned and subjected to one round of 2D classification and masked 3D classification to remove particles with poorly resolved extranucleosomal density, yielding a final dataset of 304,433 particles. The final map was obtained by homogeneous refinement with global and local CTF refinement and reached a global resolution of 2.7 Å, as determined by the gold-standard Fourier shell correlation (FSC) criterion (0.143)^62^ (Extended Data Fig. 2b–d). This map was postprocessed using anisotropic *B*-factor sharpening in Phenix^48^ and used for model fitting, building and refinement processes and making figures.

To improve the resolution of a density blob close to the edge of the nucleosome disk (associated with the DNA) and not accounted for by the model, the 1,119,333 particles obtained from heterogeneous refinement were also processed using an alternative workflow (Extended Data Fig. 7b). After one round of 2D classification and an initial reconstruction, particles were subjected to 3D classification without alignment using a focused mask around the DNA. The two best classes showing DNA-associated density were selected and particles were reextracted with a box size of 400 × 400 pixels. A subsequent round of 3D classification was performed using a tighter focused mask. A total of 60,880 particles were selected and subjected to homogeneous refinement, followed by global and local CTF refinement to correct residual aberrations. The final reconstruction reached a global resolution of 3.3 Å, as determined by the gold-standard FSC criterion (0.143)^62^ (Extended Data Fig. 7a–d). The map was postprocessed using anisotropic sharpening in PHENIX^48^ and used for figure generation.

For the nucleosome-bound PRC1 complex dataset, CTF parameters were estimated and, on the basis of CTF quality and estimated resolution, 22,602 micrographs were selected for further analysis (Extended Data Fig. 5b). Initial processing followed the same workflow described for the previous dataset, yielding a clean set of 1,015,730 particles containing nucleosome-associated density after iterative homogeneous refinement. As observed for the previous dataset, the initial reconstruction showed density preferentially resolved on only one nucleosome face. Particles were reextracted with a box size of 360 × 360 pixels binned by a factor of 2 and subjected to 3D classification without alignment using a focused mask around the density bound to the nucleosome to remove particles with poor density. Selected particles were then subjected to one round of 2D classification, followed by global and local CTF refinement to correct residual aberrations. Particles were subsequently reextracted with a larger box size (400 × 400 pixels) and subjected to 3D classification using a focused mask covering only the upper lobe of the PRC1 density. Iterative classification rounds were performed to remove particles lacking this upper-lobe density. Because of the high conformational flexibility of this region, the final dataset was reduced to 33,810 particles displaying well-defined upper-lobe density. These particles were used for homogeneous refinement with global and local CTF refinement, yielding a final reconstruction at a global resolution of 3.7 Å, as determined by the gold-standard FSC criterion (0.143)^62^ (Extended Data Fig. 5b–f).

### Model building and map validation

Atomic model fitting was performed only for the nucleosome‑bound PRC1–UBCH5C dataset. Available crystal and NMR structures and AF3 (ref. ^33^) predictions were fitted into the final refined cryo‑EM maps using rigid‑body fitting in UCSF ChimeraX (version 1.10.1)^49^. Models were manually adjusted in Coot^63^ and refined in real space using PHENIX^64^. For initial model building, the crystal structure of the *Xenopus* nucleosome core particle (PDB 2NZD)^65^ was first fitted into the density using ChimeraX. The remaining density was accounted for by piecewise docking of fragments derived from previously determined structures, including the minimal RING1B_2–116_:BMI1_2–109_ heterodimer fused to UBCH5C (PDB 4R8P)^29^, the BMI1 RAWUL and PHC2 HD domains (PDB 2NA1)^25^ and the RING1B RAWUL domain in complex with the CBX7 C‑box (PDB 3GS2)^26^. AF3 predictions were used to guide the relative positioning of these domains and to assist modeling of regions not resolved in previous structures. RING1B residues 116–136, BMI1 residues 116–129 and PHC2 residues 51–56 were built de novo and fitted into the corresponding cryo‑EM density. The resulting composite model was subjected to iterative cycles of real‑space refinement in PHENIX, interspersed with manual inspection and correction in Coot. Final refinement was performed against the sharpened cryo‑EM map. Model quality and refinement progress were assessed using map‑to‑model correlation coefficients, geometric validation metrics and map‑versus‑model FSC curves (Table 1).

### EMSA

Binding of proteins to unmodified or H3Kc27me3 mononucleosomes was assessed using EMSAs as described previously^46^. Each experiment was performed in triplicate or more, as indicated in the figure legends. Fluorescence of ATTO647N-labeled nucleosomes was quantified using ImageJ (version 1.53). Background subtraction and calculation of the fraction of bound nucleosomes were performed in MATLAB as previously reported^46^.

### Chemical crosslinking and MS

To perform crosslinking MS, 2 μM PRC1 was incubated with or without an equal amount of UBCH5C and 200 nM of unmodified or H3Kc27me3 mononucleosomes. Then, 0.5 mM BS3 (Thermo Fisher Scientific) was added to the mixture in buffer containing 25 mM HEPES pH 7.5 and 50 mM NaCl. The crosslinking reaction was run for 30 min at room temperature and then quenched by adding 20 mM Tris-HCl pH 7.5. Crosslinked samples were lysed by the addition of an equal volume of 8 M urea in 50 mM Tris, followed by sonication using a Bioruptor Plus system (Diogenode) for ten cycles of 30 s at high intensity. Reduction and alkylation were performed by adding 10 mM TCEP and 40 mM CAA. Approximately 5 µg of peptide material was then loaded onto homemade solid-phase extraction capture (SPEC) tips containing strong cation-exchange material and processed according to the SPEC workflow described previously^66^. Peptides were eluted from the SPEC tips with 1% formic acid and approximately 400 ng of peptide material was subsequently loaded onto Evotips (Evosep).

Peptides were eluted from the Evotips onto an Aurora Elite C18 column (15 cm × 75 µm, particle size: 1.7 µm; IonOpticks) using the Evosep Eno high-performance liquid chromatography (HPLC) system with the Whisper Zoom 20 samples per day (SPD) method. MS analysis was performed on an Orbitrap Eclipse MS instrument (Thermo Fisher Scientific) equipped with a FAIMS Pro interface operated at standard resolution, using compensation voltage ranges of −50 to −60 V and −45 to −55 V, in data-dependent acquisition mode. Full MS scans were acquired over the *m*/*z* range of 350–1,450 at a resolution of 60,000 (at *m*/*z* 200). The 15 most intense precursor ions were selected for fragmentation by stepped higher-energy C-trap dissociation with normalized collision energies of 19, 27 and 35. MS2 spectra were acquired at a resolution of 30,000 (at *m*/*z* 200) across a dynamic *m*/*z* range. Automatic gain control targets were set to 300% for MS1 and 100% for MS2, with a maximum injection time of 25 ms for MS1 and set to ‘auto’ for MS2. Precursor ions with a charge state of +2 were excluded to enrich for crosslinked species.

Raw data were processed using Proteome Discoverer version 2.5.0.400. Trypsin/P was specified as the protease, allowing up to two missed cleavages. Database searches were performed against a FASTA file containing the sequences of the proteins of interest, with DSS/BS3 specified as the crosslinker. Carbamidomethylation of cysteine residues was set as a fixed modification, while methionine oxidation and protein N-terminal acetylation were included as variable modifications. Identifications were accepted only if they met a minimum score of 40 and a minimum Δ score of 4. Results were filtered to a 1% false discovery rate at both the crosslinked spectrum match and crosslink levels.

Crosslinking data were visualized using XiView software^67^. To analyze CBX7 interactions with unmodified or H3Kc27me3-modified mononucleosomes, we included samples prepared either in the presence or absence of UBCH5C, as UBCH5C did not influence the CBX7 chromodomain binding to H3Kc27me3. To investigate interactions between PHC2 and UBCH5C, we analyzed all samples containing UBCH5C, irrespective of the presence or absence of H3Kc27me3, as this histone modification did not affect PHC2 binding.

### *Drosophila* mutant alleles used in this study

The *ph*^*KO*^ was generated in this study as described below. The *ph*^504^ null allele was described previously^68^.

### Generation of the *ph*^*KO*^ deletion allele and of *Ph* transgenes

The *ph*^*KO*^ short chromosomal deletion allele was generated using standard CRISPR–Cas9 technology. Two genomic target sites, chrX:2116678–2116700 (reverse strand) and chrX:2131821–2131843 (forward strand), were selected for CRISPR–Cas9 cleavage. For each gRNA, the 20-nt target sequence, excluding the PAM sequence, was cloned into the pCFD3-dU6:3gRNA plasmid (Addgene, plasmid 49410). DNA fragments corresponding to the left and right homology arms flanking the intended deletion (chrX:2115920–2116683 and chrX:2131838–2132562) were cloned into the pDsRed-attP donor plasmid (Addgene, plasmid 51019). The genomic sequence of the resulting engineered allele is shown in Extended Data Fig. 8a. The *>ph*^*+*^*>* rescue cassette consisting of the genomic fragment spanning chrX:2112745–2132046 and the *white*^*+*^ and *UASp–GFP* markers, all flanked by FRT sites, were cloned into attB-P[acman]-ApR (RRID:DGRC_1245) (Extended Data Fig. 8b). The *V5–Ph*^*WT*^ transgene construct contained the complete *ph-d* genomic locus (chrX:2112745–2126116), with an in-frame V5 epitope tag inserted at the 5′ end of the coding regions of both annotated transcripts, *ph-d-RA* and *ph-d-RB*. *V5–Ph*^*∆HD*^ and *V5–Ph*^*∆SAM*^ were generated by mutagenesis of the *V5–Ph*^*WT*^ construct, deleting the *ph-d* genomic sequences corresponding to chrX:2117700–2117801 and chrX:2116869–2117105, respectively. *V5–Ph*^*WT*^, *V5–Ph*^*∆HD*^ and *V5–Ph*^*∆SAM*^ were integrated into the *Drosophila* genome using PhiC31-mediated site-specific recombination at the attP landing sites 86Fb (BDSC, 130437). All genomic coordinates refer to dm6 version of the *Drosophila* *melanogaster* genome.

### Genotypes of animals used in the different figures

In Fig. 4g,h, the genotypes were as follows: no TG, Oregon-R; *V5–Ph*^*WT*^: *w*; *V5–Ph*^*WT*^ *(86Fb)*; *V5–Ph*^*∆HD*^: *w*; *V5–Ph*^*∆HD*^ *(86Fb)*; *V5–Ph*^*∆SAM*^: *w*; *V5–Ph*^*∆SAM*^ *(86Fb)*.

In Fig. 5 and Extended Data Fig. 10, the genotypes *wt*, *ph*^*KO*^ and *ph*^*∆SAM*^ were generated as described in Extended Data Fig. 8c–e. Briefly, excision of the *>ph*^*+*^*>* cassette occurs in the germline of *>ph*^*+*^*>* heterozygous or hemizygous parents. The resulting GFP-negative embryos correspond to the desired progeny. Chromosomes carrying *V5–Ph*^*WT*^ and *V5–Ph*^*∆SAM*^ inserted at the 86Fb landing site were recombined with a chromosome bearing the UASp–FLP transgene at the VK33 landing site.

In Extended Data Fig. 9a, *Or-R* denotes Oregon-R. The *wt* was obtained as GFP-negative progeny from mothers of genotype *ph*^*KO*^/*FM7c twi-Gal4 UAS–GFP* and fathers of genotype *ph*^*KO*^/ Y.w^+^; *V5–Ph*^*WT*^ *(86Fb)*. The GFP-negative progeny segregated into two distinct classes: in class 1, the *Abd-B* expression pattern was indistinguishable from Oregon-R embryos. These were presumed to be of genotype: *ph*^*KO*^*/Y.w*^*+*^*; V5–Ph*^*WT*^ *(86Fb)/+*. Class 2 embryos displayed a small number of cells misexpressing *Abd-B* gene product (Extended Data Fig. 9a) and these were presumed to be of genotype *ph*^*KO*^*; V5–Ph*^*WT*^ *(86Fb)/+*. The cuticles of both class 1 and class 2 embryos appeared *wt*. *ph*^*KO*^ was obtained as GFP-negative progeny from mothers of genotype *ph*^*KO*^/*FM7c twi-Gal4 UAS–GFP* and fathers of genotype *ph*^*KO*^/Y.w^+^. The GFP-negative progeny segregated into two distinct classes. In class 1, the *Abd-B* expression pattern was indistinguishable from Oregon-R embryos. These were presumed to be of genotype *ph*^*KO*^*/Y.w*^*+*^. Class 2 embryos displayed a massive misexpression of *Abd-B* gene product and their cuticle showed severe morphological defects. These were presumed to be the *ph*^*KO*^ embryos. *ph*^*∆SAM*^ was obtained as GFP-negative progeny from mothers of genotype *ph*^*KO*^/*FM7c twi-Gal4 UAS–GFP* and fathers of genotype *ph*^*KO*^/Y.*w*^*+*^; *V5–Ph*^*∆SAM*^ *(86Fb)*. The GFP-negative progeny segregated into two distinct classes. In class 1, the *Abd-B* expression pattern was indistinguishable from Oregon-R embryos. These were presumed to be of genotype *ph*^*KO*^*/Y.w*^*+*^; *V5–Ph*^*∆SAM*^ *(86Fb)/+*. Class 2 embryos displayed extensive misexpression of *Abd-B* gene product and their cuticle showed severe morphological defects. These were presumed to be of genotype *ph*^*KO*^*; V5–Ph*^*∆SAM*^ *(86Fb)*. Y.*w*^*+*^ corresponds to an interchromosomal duplication of the cytological regions 2D2 to 3D3, including the *ph*^+^ and *white*^*+*^ gene loci, onto the Y chromosome.

In Extended Data Fig. 9b, the *wt* was obtained as GFP-negative progeny from mothers of genotype *ph*^*KO*^/*FM7c twi-Gal4 UAS*–*GFP*; *V5–Ph*^*WT*^ *(86Fb)* and fathers of genotype *>ph*^*+*^*>/Y; NGVP16/UASp–FLP*, in which the *>ph*^*+*^*>* rescue cassette is excised in their germ cells. The cross used to generate the fathers is described in Extended Data Fig. 8c. *ph*^*KO*^ was obtained as GFP-negative progeny from mothers of genotype *ph*^*KO*^/*FM7c twi-Gal4 UAS–GFP* and fathers of genotype *>Ph*^*+*^*>/Y; NGVP16/UASp–FLP*, in which the *>ph*^*+*^*>* rescue cassette is excised in their germ cells. The cross used to generate the fathers is described in Extended Data Fig. 8c. *ph*^*∆SAM*^ was obtained as GFP-negative progeny from mothers of genotype *ph*^*KO*^/*FM7c twi-Gal4 UAS–GFP; V5–Ph*^*∆SAM*^ *(86Fb)* and fathers of genotype *>ph*^*+*^*>/ Y; NGVP16/UASp–FLP*, in which the *>ph*^*+*^*>* rescue cassette is excised in their germ cells. The cross used to generate the fathers is described in Extended Data Fig. 8c. For NGVP16, the genotype was *nanos–Gal4–VP16*. For UASp*–*FLP, FLP recombinase was expressed under control of the *UASGal4–P-element promoter*.

### Antibodies used in this study

**Table:** 

Antibody	Reference	Assays
V5	Thermo Fisher (R960-25)	Affinity purification
V5	Cell Signaling (D3H8Q)	ChIP, western blot
H2AK119ub Psc	Cell Signaling (D27C4) DSHB (6E8)	ChIP, western blot ChIP
Pho	Papp and Müller^69^	ChIP
Lamin Dm0	DSHB (ADL67.10)	Western blot
H2B	Finogenova et al.^53^	Western blot
Abd-B	DSHB (1A2E9)	Immunohistochemistry

### Affinity purification from embryonic extracts

Embryos (2–16 h old) expressing V5-tagged *Ph*^*WT*^, Ph^ΔSAM^ or Ph^ΔHD^ and Oregon-R embryos as control were dechorionated, washed thoroughly, flash-frozen in liquid nitrogen and stored at −80 °C. Frozen embryos were transferred to a SPEX Freezer/Mill 6875 cryomill and lysed in liquid nitrogen. Samples were processed at 15 Hz (cycles per second) for six cycles of 2 min each, with 1-min cooling intervals between cycles. The resulting embryo powder was stored at −80 °C until use.

For affinity purification, three independent protein extracts were reconstituted by resuspending 0.8 g of embryo powder in 3 ml of lysis buffer P50 (50 mM potassium phosphate pH 8.0 and 0.1% NP-40), supplemented with 1× protease inhibitor cocktail (Roche, 04693132001). Lysates were incubated with 50 μl of Protein G Dynabeads (Invitrogen, 10004D) precoupled to anti-V5 monoclonal antibody for 30 min at 4 °C with gentle rotation. Beads were washed three times with P50 buffer.

### LC–MS/MS and differential protein abundance analyses

On-bead protein digestion was performed by incubating bead-bound proteins in 50 µl of SDC buffer (1% sodium deoxycholate, 40 mM 2-chloroacetamide, 10 mM Tris(2-carboxyethyl)phosphine and 100 mM Tris, pH 8.0) for 20 min at 37 °C. Proteins were digested overnight at 37 °C with 0.5 µg of trypsin (Promega). Peptides in the supernatant were separated from the beads using a magnetic rack. The peptide solution was acidified to a final concentration of 1% trifluoroacetic acid (Merck) and purified using SCX StageTips. Lastly, approximately 200 ng of peptides were loaded onto Evosep Pure tips (Evosep).

Peptides were eluted from Evotips onto a 15-cm PepSep C18 column (15 cm × 150 µm, 1.5 µm; Bruker Daltonics) using an Evosep One HPLC system. The column was maintained at 50 °C and peptides were separated using the 30 SPD method. Data were acquired on a timsTOF Pro MS instrument operated with timsControl software in data-independent acquisition (DIA) parallel accumulation–serial fragmentation (PASEF) mode. The MS scan range was 100–1,700 *m*/*z* with an ion mobility range of 1/*K*_0_ = 0.70–1.30 V s cm^−^^2^. Equal ion accumulation and ramp times of 100 ms were used in the dual TIMS analyzer, yielding a spectral rate of 9.52 Hz. DIA-PASEF scans were acquired in the range of 350.2–1,199.9 Da with 42 DIA-PASEF windows assigned to one TIMS scan each. Precursor isolation windows were alternated, resulting in an overall cycle time of 2.21 s. The collision energy was linearly ramped from 45 eV at 1/*K*_0_ = 1.30 V s cm⁻^2^ to 27 eV at 1/*K*_0_ = 0.85 V s cm⁻^2^.

Raw data were processed in Spectronaut 19 or Spectronaut 20 (Biognosys) using directDIA+ (library-free) mode. Spectra were searched against a predicted drosophila database from UniProt (SwissProt and TrEMBL). Cysteine carbamidomethylation was set as a fixed modification and methionine oxidation and protein N-terminal acetylation were set as variable modifications. Protein quantification across samples was performed using label-free quantification (MaxLFQ) at the MS^2^ level.

Differential protein abundance analysis was performed using proDA (version 1.20.0)^70^, with iBAQ (intensity-based absolute quantification) values as input. Pairwise comparisons were conducted between each V5-tagged Ph purification and a mock purification from Oregon-R embryonic extracts. Protein abundance in the V5–Ph pulldowns was calculated from the average abundance across samples (for the V5–Ph and the mock pulldown) and the fold change between conditions, as provided by proDA. Analyses were restricted to nuclear proteins on the basis of a predefined classification derived from UniProtKB taxonomy identifier 7227 (*D*. *melanogaster*) and Gene Ontology term GO:0005634 (nucleus). Significance is defined as follows: adjusted *P* < 0.15, protein abundance in the V5–Ph purification > 2^11^ and fold change between the V5–Ph and the mock purifications > 32.

### Immunohistochemistry and cuticle preparations of *Drosophila* embryos

Staining of embryos with Abd-B antibodies and cuticle preparation were performed following standard protocols.

### Western blotting from total embryo extracts

For each genotype, 50 embryos (2–16 h old) were dechorionated and collected in 50 µl of 1× NuPAGE LDS sample buffer (Life Technologies). Samples were briefly sonicated (three cycles, 30 s on, 30 s off, at maximum intensity using a Bioruptor (Diagenode)), centrifuged for 1 min at maximum speed and heated at 65 °C for 2 min. Western blot analyses were performed using standard procedures.

### Western blot analysis of H2Aub1 bulk levels and MS-based quantification of nuclear proteins from *Drosophila* embryos

Nuclei were purified from embryos and quantified as previously described^71^. Comparable numbers of nuclei from the different genotypes were analyzed either by western blotting probed with anti-H2Aub1 antibodies and H2B antibodies as a control, following standard procedures, or by quantitative MS. For MS analyses, nuclear pellets were resuspended in 50 µl of SDC buffer. Samples were heated at 95 °C for 5 min and subsequently sonicated using the Bioruptor Plus system (Diagenode) for ten cycles of 30 s at high intensity. The heating and sonication steps were repeated once more. Samples were then diluted 1:1 with MS-grade water (VWR). Protein digestion was initiated by adding 1 µg of Lys-C (Wako) and incubating for 2 h at 37 °C, followed by overnight digestion at 37 °C with 1 µg of trypsin (Promega). The resulting peptide solution was acidified to a final concentration of 1% trifluoroacetic acid (Merck) and approximately 300 ng of peptide material loaded onto Evosep Pure tips (Evosep). LC–MS/MS analysis and raw data processing were performed as described above.

### Embryo collection, chromatin preparation and ChIP

For each genotype, embryos were collected at 1–3 h after egg laying, dechorionated, flash-frozen in liquid nitrogen and stored at −80 °C. Chromatin was isolated from nuclei as previously described^53^. ChIP experiments were performed using 200 ng of *D*. *melanogaster* chromatin. For normalization, 100 ng of independently prepared *Drosophila* *pseudoobscura* chromatin was added to each sample before antibody incubation. ChIP was carried out following the protocol described in Bonnet et al.^71^.

### Library preparation and sequencing

ChIP-seq libraries were prepared according to the manufacturer’s instructions and subjected to paired-end sequencing. Sequencing reads were aligned using STAR^72^ to the *D*. *melanogaster* dm6 genome assembly^73^ and the *D*. *pseudoobscura* dp3 genome assembly (FlyBase Release 1.03). Only uniquely mapped reads allowing a maximum of two mismatches were retained for downstream analyses.

### Normalization of ChIP-seq datasets and calculation of ChIP-seq signal

H2Aub1 ChIP-seq datasets were normalized using the ratio of *D*. *melanogaster* to *D*. *pseudoobscura* reads in both input and ChIP samples, as previously described^53^. For ChIP-seq datasets generated using V5, Psc or Pho antibodies, normalization was performed on the basis of the total number of mapped reads per sample. ChIP-seq signal was defined as the normalized number of mapped reads per million from a ChIP-seq dataset.

### Identification of Polycomb chromatin domains and PREs

Chromosome intervals referred to in this paper as Polycomb chromatin domains correspond to canonical H3K27me3 domains, as previously described^11^. Peak calling for Pho and Scm ChIP-seq datasets (from 1–3-h-old *WT* embryos) was performed using MACS 3.0. A total of 212 peaks common to both Pho and Scm datasets that overlapped Polycomb chromatin domains were identified. These regions were considered PREs.

### Reporting summary

Further information on research design is available in the Nature Portfolio Reporting Summary linked to this article.

## Online content

Any methods, additional references, Nature Portfolio reporting summaries, source data, extended data, supplementary information, acknowledgements, peer review information; details of author contributions and competing interests; and statements of data and code availability are available at https://doi.org/10.1038/s41594-026-01885-6.

## Supplementary information

Reporting Summary

Peer Review File

## Source data

Source Data Fig. 1 Unprocessed gels.

Source Data Fig. 1 Statistical quantification of analyses.

Source Data Fig. 2 Unprocessed gels.

Source Data Fig. 3 Unprocessed gels.

Source Data Fig. 3 Statistical quantification of analyses.

Source Data Fig. 4 Unprocessed gels.

Source Data Fig. 5 Unprocessed gels.

Source Data Extended Data Fig. 1 Unprocessed gels.

Source Data Extended Data Fig. 1 Statistical quantification of analyses.

Source Data Extended Data Fig. 6 Unprocessed gel.

Source Data Extended Data Fig. 7 Unprocessed gels.

Source Data Extended Data Fig. 7 Statistical quantification of analyses.

## Data Availability

The protein structure data reported in this study were deposited to the Protein Data Bank under accession code PDB 28OE and the EM Data Bank under accession codes EMD-56669 and EMD-56717. The MS data from the crosslinking and pulldown experiments were deposited to PRIDE under accession code PXD074618. The genomic data were deposited to the Gene Expression Omnibus under accession number GSE320532. Plasmids, viruses and *Drosophila* strains generated in this study are available upon request. Source data are provided with this paper.
