# Supplementary material for: Cryo-EM structure, enzymatic activity and genome targeting of canonical PRC1
Source: Nat Struct Mol Biol. 2026 Sep 14;33(10):1516–26. doi: 10.1038/s41594-026-01885-6 (PMC13672072; doi:10.1038/s41594-026-01885-6)

Source Data Extended Fig.1

Panel b:

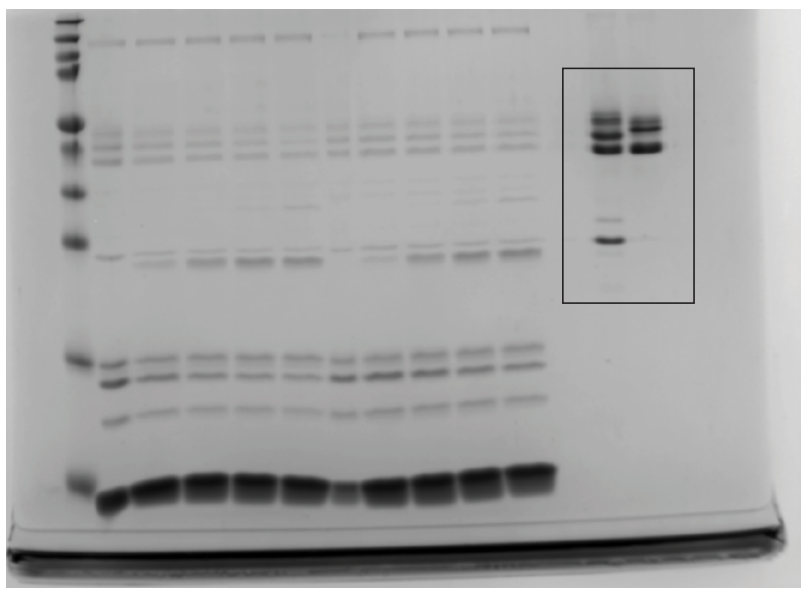

Panel c:

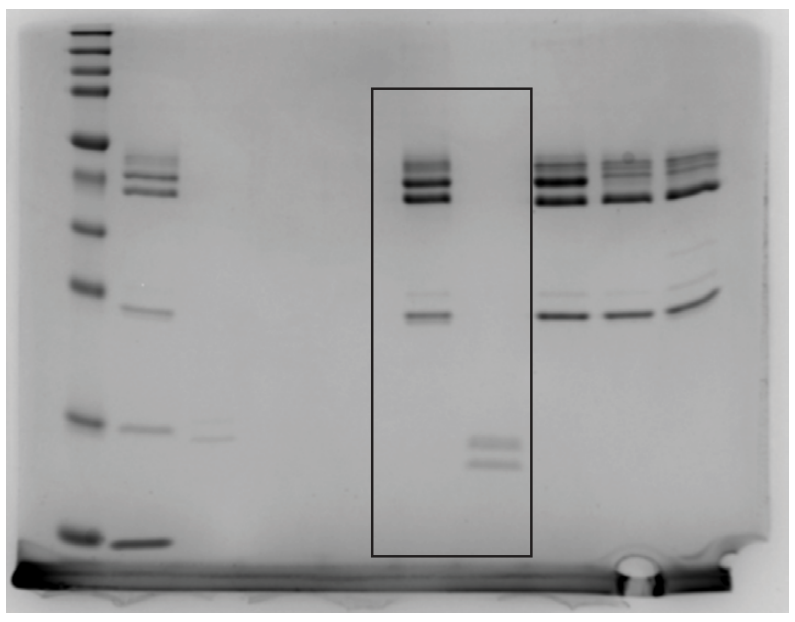

Panel d:

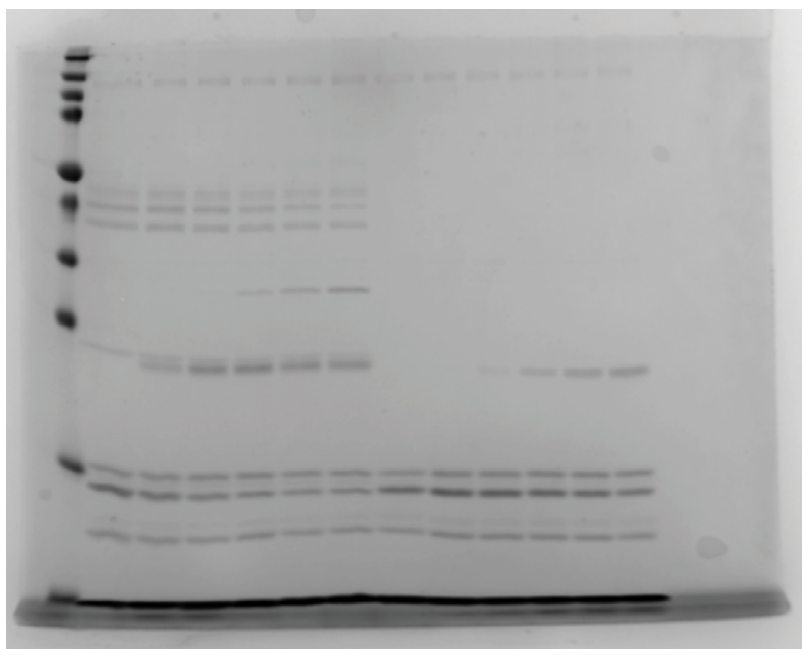

Supplement: Supplementary file 1 [file 41594_2026_1885_MOESM10_ESM.pdf]
