# Supplementary material for: Cryo-EM structure, enzymatic activity and genome targeting of canonical PRC1
Source: Nat Struct Mol Biol. 2026 Sep 14;33(10):1516–26. doi: 10.1038/s41594-026-01885-6 (PMC13672072; doi:10.1038/s41594-026-01885-6)
Supplement: Supplementary file 4 [file 41594_2026_1885_MOESM1_ESM.pdf]

nature portfolio

Corresponding author(s): Jürg Müller
Jacques Bonnet

Last updated by author(s): 2026/08/12

Reporting Summary

Nature Portfolio wishes to improve the reproducibility of the work that we publish. This form provides structure for consistency and transparency in reporting. For further information on Nature Portfolio policies, see our Editorial Policies and the Editorial Policy Checklist.

Statistics

For all statistical analyses, confirm that the following items are present in the figure legend, table legend, main text, or Methods section.

n/a	Confirmed	
☐	☒	The exact sample size (*n*) for each experimental group/condition, given as a discrete number and unit of measurement
☐	☒	A statement on whether measurements were taken from distinct samples or whether the same sample was measured repeatedly
☐	☒	The statistical test(s) used AND whether they are one- or two-sided<br>*Only common tests should be described solely by name; describe more complex techniques in the Methods section.*
☒	☐	A description of all covariates tested
☐	☒	A description of any assumptions or corrections, such as tests of normality and adjustment for multiple comparisons
☐	☒	A full description of the statistical parameters including central tendency (e.g. means) or other basic estimates (e.g. regression coefficient) AND variation (e.g. standard deviation) or associated estimates of uncertainty (e.g. confidence intervals)
☒	☐	For null hypothesis testing, the test statistic (e.g. *F*, *t*, *r*) with confidence intervals, effect sizes, degrees of freedom and *P* value noted<br>*Give P values as exact values whenever suitable.*
☒	☐	For Bayesian analysis, information on the choice of priors and Markov chain Monte Carlo settings
☒	☐	For hierarchical and complex designs, identification of the appropriate level for tests and full reporting of outcomes
☒	☐	Estimates of effect sizes (e.g. Cohen's *d*, Pearson's *r*), indicating how they were calculated

Our web collection on statistics for biologists contains articles on many of the points above.

Software and code

Policy information about availability of computer code

Data collection	SerialEM 4.0
Data analysis	CryoSparc v4.7.0<br>MotionCorr2 1.1<br>CTFFIND 4.1.13.<br>Coot 0.9.8.7<br>PHENIX 1.20<br>UCSF ChimeraX v1.10.1<br>ImageJ 1.53<br>Matlab R2022B<br>Prism 9<br>Image Lab v6.1<br>XiView<br>star 2.7.11b<br>samtools 1.23<br>deeptools 3.5.6<br>bioconductor R 4.3.1<br>MACS 3.0

For manuscripts utilizing custom algorithms or software that are central to the research but not yet described in published literature, software must be made available to editors and reviewers. We strongly encourage code deposition in a community repository (e.g. GitHub). See the Nature Portfolio guidelines for submitting code & software for further information.

Data

Policy information about availability of data

All manuscripts must include a data availability statement. This statement should provide the following information, where applicable:

- Accession codes, unique identifiers, or web links for publicly available datasets
- A description of any restrictions on data availability
- For clinical datasets or third party data, please ensure that the statement adheres to our policy

The protein structure data reported in this study have been deposited in PDB under the accession codes PDB ID 28OE and in the EMDB under accession codes EMD 56669 and EMD 56717.
Initial models used for CryoEM model building (PDB code): 2NZD, 4R8P, 2NAI, 3GS2.
The mass spectrometry data from the cross-linking and pull-down experiments have been deposited in PRIDE under the accession code PXD074618.
The genomic data have been deposited in GEO under accession number GSE320532.

Research involving human participants, their data, or biological material

Policy information about studies with human participants or human data. See also policy information about sex, gender (identity/presentation), and sexual orientation and race, ethnicity and racism.

Reporting on sex and gender	N/A
Reporting on race, ethnicity, or other socially relevant groupings	N/A
Population characteristics	N/A
Recruitment	N/A
Ethics oversight	N/A

Note that full information on the approval of the study protocol must also be provided in the manuscript.

Field-specific reporting

Please select the one below that is the best fit for your research. If you are not sure, read the appropriate sections before making your selection.

☒ Life sciences ☐ Behavioural & social sciences ☐ Ecological, evolutionary & environmental sciences

For a reference copy of the document with all sections, see nature.com/documents/nr-reporting-summary-flat.pdf

Life sciences study design

All studies must disclose on these points even when the disclosure is negative.

Sample size	The numbers of particles used for cryo-EM reconstructions are indicated in Table 1<br>For all EMSAs and enzyme activity assays, we conducted experiments with three or more replicates to ensure reproducibility.<br>The consistent results across all replicates suggests that the number of replicates was sufficient in all experiments.<br>No statistical methods were used to predetermine the sample size.
Data exclusions	No data were excluded for enzyme activity assays. Two data points were excluded from the EMSA assays due to a technical issue during gel loading, as indicated in the source data file.
Replication	For quantitative measurements, experiments were performed at least in triplicate. All of them were successful.
Randomization	not relevant, no grouped samples
Blinding	not relevant, no grouped samples

Reporting for specific materials, systems and methods

We require information from authors about some types of materials, experimental systems and methods used in many studies. Here, indicate whether each material, system or method listed is relevant to your study. If you are not sure if a list item applies to your research, read the appropriate section before selecting a response.

Materials & experimental systems

n/a	Involved in the study
☐	☒ Antibodies
☐	☒ Eukaryotic cell lines
☒	☐ Palaeontology and archaeology
☐	☒ Animals and other organisms
☒	☐ Clinical data
☒	☐ Dual use research of concern
☒	☐ Plants

Methods

n/a	Involved in the study
☐	☒ ChIP-seq
☒	☐ Flow cytometry
☒	☐ MRI-based neuroimaging

Antibodies

Antibodies used	V5, Thermo Fisher (R960-25), Affinity-purification<br>V5, Cell Signaling (D3H8Q), ChIP, Western Blot<br>H2AK119ub, Cell Signaling (D27C4), ChIP, Western Blot<br>Pho Papp and Müller 2006, ChIP<br>Psc, DSHB (6E8), ChIP<br>Scm, Gambetta et al 2009, ChIP<br>Lamin Dm0, DSHB (ADL67.10) Western Blot<br>H2B, Finogenova et al 2020, Western Blot<br>Abd-B, DSHB (1A2E9), Immunohistochemistry
Validation	Wherever possible, antibodies were previously validated in mutant animals/conditions.

Eukaryotic cell lines

Policy information about cell lines and Sex and Gender in Research

Cell line source(s)	Cell line (Trichoplusia ni)<br>HighFive cell line for expression Invitrogen Product nr.:<br>B85502<br>BTI-Tn-5B1-4<br>(RRID:CVCL_C190)<br>Protein expression<br>Cell line (Spodoptera frugiperda)<br>Sf21 cell line for<br>Baculovirus production<br>Invitrogen Product nr.:<br>1149701<br>(RRID:CVCL_0518)
Authentication	Commercial cell lines. Authentication is performed by the manufacturer
Mycoplasma contamination	The cell line were not tested for mycoplasma contamination
Commonly misidentified lines<br>(See ICLAC register)	No commonly misidentified lines were used

Animals and other research organisms

Policy information about studies involving animals; ARRIVE guidelines recommended for reporting animal research, and Sex and Gender in Research

Laboratory animals	Drosophila melanogaster (wild-type and transgenic animals; all genotypes are described in the Method section),<br>Drosophila pseudoobscura (wild-type animals). No ethical approval was required
Wild animals	N/A
Reporting on sex	N/A
Field-collected samples	N/A
Ethics oversight	N/A

Note that full information on the approval of the study protocol must also be provided in the manuscript.

Plants

Seed stocks	N/A
Novel plant genotypes	N/A
Authentication	N/A

ChIP-seq

Data deposition

☒ Confirm that both raw and final processed data have been deposited in a public database such as GEO.

☒ Confirm that you have deposited or provided access to graph files (e.g. BED files) for the called peaks.

Data access links<br>*May remain private before publication.*	All ChIP-seq datasets used for the genomic analyses are listed in the GEO entry: GSE320532.
Files in database submission	See detailed list in GEO entry: GSE320532.
Genome browser session<br>(e.g. UCSC)	N/A

Methodology

Replicates	At least two biological replicates were used for all experiments, except for Psc ChIP in ph[KO] embryos where a single dataset was generated.
Sequencing depth	Paired-end sequencing with ≥1M reads mapped to the Drosophila melanogaster dm6 genome.
Antibodies	V5, Cell Signaling (D3H8Q)<br>Psc, DSHB (6E8)<br>H2AK119ub, Cell Signaling (D27C4)<br>Pho, Papp and Müller 2006<br>Scm, Gambetta et al 2009
Peak calling parameters	MACS 3.0 was used to identify peaks, with standard parameters
Data quality	N/A
Software	star 2.7.11b<br>samtools 1.23<br>deeptools 3.5.6<br>bioconductor R 4.3.1<br>MACS 3.0
