# Supplementary material for: Cryo-EM structure, enzymatic activity and genome targeting of canonical PRC1
Source: Nat Struct Mol Biol. 2026 Sep 14;33(10):1516–26. doi: 10.1038/s41594-026-01885-6 (PMC13672072; doi:10.1038/s41594-026-01885-6)
Supplement: Supplementary file 5 [file 41594_2026_1885_MOESM2_ESM.pdf]

nature portfolio

Peer Review File

Cryo-EM structure, enzymatic activity and genome targeting of canonical PRC1

Corresponding Author: Dr Juerg Mueller

Version 0:

Decision Letter:

19th Mar 2026

Dear Dr. Mueller,

Thank you again for submitting your manuscript "Structure of a human PRC1 holocomplex reveals basis of enzymatic activity and genomic targeting". I apologise for the delay in sharing our decision. We have decided to send your manuscript for peer review, but several points require your attention before we can proceed with peer review.

I am re-opening the manuscript submission system for you to resubmit your manuscript with all associated files needed for peer review directly (maps & models, reporting summary, other relevant info), within 2-3 business days if possible. Please follow the link at the bottom of this email to upload the documents listed below. If you have any issues, please reach out to us before completing the submission.

1. STRUCTURAL DATA:

We require official wwPDB validation reports for newly described atomic structures per journal policy. We also request that authors provide cryo-EM maps, half-maps and models, as well as maps and models obtained from subtomogram averaging if it applies to the work, to help the reviewers in assessing the work. We recommend the use of figshare in our system, which allows for provision of anonymous access links for the referees (https://www.springernature.com/gp/authors/research-data/figshare-integration). Alternatively, please upload .zip folders directly with the submission.
Manuscripts reporting new structures should contain a table summarizing structural and refinement statistics. Please include these tables for new cryo-EM or -ET (https://www.nature.com/documents/nr-tables-cryo-em.doc and modifying this file for ET), NMR (https://www.nature.com/documents/nr-tables-nmr.doc) and X-ray crystallography (https://www.nature.com/documents/nr-tables-xray.doc). To facilitate assessment of the quality of the structural data, a stereo image of a portion of the electron density map (for crystallography papers) or of the superimposed lowest energy structures (>10; for NMR papers) should be provided with the submitted manuscript. If the reported structure represents a novel overall fold, a stereo image of the entire structure (as a backbone trace) should be provided. For cryo-EM structures, a representative micrograph showing individual particles should be provided in the figures alongside a processing workflow (please see our Editorial for more information: https://www.nature.com/articles/s41594-025-01567-9 and here for an example: https://www.nature.com/articles/s41594-025-01586-6/figures/7).

2. REPORTING SUMMARY:

We want to ensure that the methods and statistics reporting in our papers are of the highest quality. To that end, we ask authors to fill out a Reporting Summary that collects information on experimental design and reagents. If your paper includes ChIP-seq or flow cytometry data, we ask that you take special care to complete those sections of the Reporting Summary as this data will aid greatly in the review of your manuscript.

Reporting Summary:
https://www.nature.com/documents/nr-reporting-summary.pdf

3- PROTEOMICS AND SEQUENCING:

Please provide accession numbers and reviewer tokens to access sequencing or proteomics datasets since unpublished datasets are part of your study. Please add this information to your manuscript file in the Data Availability Section, as the reviewers will be asked to check these source proteomics and sequencing data. Proteomics data should be deposited to

any ProteomeXchange member repository (http://www.proteomexchange.org/) and DNA sequencing, RNA sequencing, genome assembly data should be deposited to any INSDC member repository (https://www.insdc.org/) or Genome Sequence Archive.

4- CODE, MD SIMULATIONS, AND MACHINE LEARNING REPORTING:

Please provide the code used to analyse the data if newly developed (unpublished) code was used in the work. For the reviewers to evaluate the work adequately, they must be able to test the software/review the code themselves. If you have not yet provided the software, we therefore request that you provide a single compressed zip file containing the software with a readme.txt file or other user manual containing complete instructions for installing and running the software. If appropriate, please provide example data and expected output. Sufficient material should be provided for referees to directly test the performance of the software/algorithm. Alternatively, you can upload the code to GitHub and provide the link directly in the manuscript.

Please fill out and upload the code and software submission checklist that will be made available to editors and reviewers. This form is a dynamic 'smart pdf' and must therefore be downloaded and completed in Adobe Reader, instead of opening it in a web browser.
https://www.nature.com/documents/nr-software-policy.pdf

If molecular dynamics (MD) simulations were performed, please refer to this Editorial and provide a completed MD simulations checklist with your manuscript: https://www.nature.com/articles/s42003-023-04653-0

Lastly, if it applies, please complete the completed machine learning checklist with your submission and upload it for review: https://www.nature.com/documents/machine-learning-checklist.pdf

Please use the link below to submit the files. **Please also remember to move forward all other files associated with this version of the paper.**

Link Redacted

Note: This URL links to your confidential home page and associated information about manuscripts you may have submitted, or that you are reviewing for us. If you wish to forward this email to co-authors, please delete the link to your homepage.

We look forward to seeing the revised manuscript and thank you for the opportunity to review your work.

Sincerely,

Dimitris Typas
Senior Editor
Nature Structural & Molecular Biology
ORCID: 0000-0002-8737-1319

Version 1:

Decision Letter:

22nd Apr 2026

Dear Dr. Mueller,

Thank you again for submitting your manuscript "Structure of a human PRC1 holocomplex reveals basis of enzymatic activity and genomic targeting" to the journal. We now have comments (below) from the 3 reviewers who evaluated your paper. In light of these reports, we remain interested in your study and would like to see your response to the comments of the referees in the form of a revised manuscript.

You will see that all three referees appreciate the potential conceptual novelty imparted by the study, yet they raise notable concerns that need to be addressed in a revised manuscript. In particular, Reviewers #1 and #2 request more suitable comparisons of the ubiquitin ligase activities of the holocomplex with the full-length RING1B/BMI1 dimer. In line with these concerns, we agree with the Referees' request to tone down of overstatements, including the title of the work. Furthermore, Reviewer #2 requests that additional, more subtle HD domain mutants be tested to assess the functional contributions of the interaction of PHC2 HD domain with UBCH5C, also in the fly system. While we would not expect that you present a series of mutations to address these points, we think that testing at least 1-2 representative mutants that fit the rationale of the Reviewers with respect to more nuanced effects and showing their functional significance would be needed. Further to these points, Reviewer #1 requests that preferred orientation issues in the analyses of the structural data are addressed. Finally, all Reviewers request clarifications, further comparisons, and textual changes to improve clarity and tone down some statements, .

Please be sure to address/respond to all concerns of the referees in full in a point-by-point response and highlight all changes in the revised manuscript text file. We expect to see your revised manuscript within the next 2-3 months. We are committed to providing a fair and constructive peer-review process; please do not hesitate to contact us if you would like to discuss the reviews or anticipate any delay with the resubmission. If you cannot send it within this time, please contact us to discuss an extension; we will still consider your revision provided that no similar work has been accepted for publication at NSMB or published elsewhere.

REPORTING AND DATA AVAILABILITY: Please provide updated files as follows with your revision. As you already know, we put great emphasis on ensuring that the methods and statistics reported in our papers are correct and accurate.

1. REPORTING SUMMARY: Please provide an updated file:

https://www.nature.com/documents/nr-reporting-summary.pdf

This form is a dynamic ‘smart pdf’ and must be downloaded and completed in Adobe Reader.

2. REPORTING OF STRUCTURAL DATA: If there are additional or modified structures in the revision, please submit the corresponding PDB validation reports with your other files, and use the figshare integration system to provide access to maps, half-maps, and models.

Manuscripts reporting new structures should contain a table summarizing structural and refinement statistics. Please include these tables for new cryo-EM or -ET (https://www.nature.com/documents/nr-tables-cryo-em.doc and modifying this file for ET), NMR (https://www.nature.com/documents/nr-tables-nmr.doc) and X-ray crystallography (https://www.nature.com/documents/nr-tables-xray.doc). To facilitate assessment of the quality of the structural data, a stereo image of a portion of the electron density map (for crystallography papers) or of the superimposed lowest energy structures (>10; for NMR papers) should be provided with the submitted manuscript. If the reported structure represents a novel overall fold, a stereo image of the entire structure (as a backbone trace) should also be provided. For cryo-EM structures, a representative micrograph showing individual particles should be provided in the figures alongside a processing workflow (please see our Editorial for more information: https://www.nature.com/articles/s41594-025-01567-9).

DATA DEPOSITION: We require deposition of coordinates (and, in the case of crystal structures, structure factors) into the Protein Data Bank with the designation of immediate release upon publication (HPUB). Electron microscopy-derived density maps and coordinate data must be deposited in EMDB and released upon publication. Deposition and immediate release of NMR chemical shift assignments are requested. To avoid delays in publication, dataset accession numbers must be supplied with the revised manuscript and appropriate release dates must be indicated at the galley proof stage.

3. REPORTING OF LIGHT MICROSCOPY DATA: For any revision that includes light microscopy data, we ask our authors to please include a completed light microscopy reporting table [https://www.nature.com/documents/Light_microscopy_reporting_table.xlsx] to ensure the methods are described thoroughly. The table will be available to reviewers and

ultimately published, should the manuscript be accepted at the journal. When submitting the revised version of your manuscript, please pay close attention to our href="https://www.nature.com/nature-portfolio/editorial-policies/image-integrity">Digital Image Integrity Guidelines.

4. CODE AND COMPUTATIONAL WORK: If newly developed, unpublished code was used in this work, it must be provided with the revision and access to it must be disclosed in the Code Availability Statement in the manuscript. Please also provide the completed software submission checklist. This form is a dynamic ‘smart pdf’ and must be downloaded and completed in Adobe Reader, instead of opening it in a web browser: https://www.nature.com/documents/nr-software-policy.pdf

If molecular dynamics (MD) simulations were performed, please refer to this Editorial and provide a completed MD simulations checklist with your revision: https://www.nature.com/articles/s42003-023-04653-0

Lastly, if it applies, please complete and upload the completed machine learning checklist with your submission for re-review: https://www.nature.com/documents/machine-learning-checklist.pdf

5. SOURCE GEL IMAGES: Unprocessed scans should be provided for all gels and western blots presented in figures and should be clearly labelled with the figure number in the file title. The source gel images should be uncropped, unmodified images, with molecular weight markers. Uncropped, unmodified gel images from existing experimental repeats, in addition to the data shown in the figures, can also be presented in source data. Please provide these files as PDF with your revision.

Please ensure that all control panels for gels and western blots are appropriately described as loading or IP controls. Lastly, please ensure that all images in the paper are checked for duplication of panels and for splicing of gel lanes.

6. SOURCE NUMERICAL DATA: we urge authors to provide, in tabular form in excel files, the data underlying the graphical representations used in figures. This is to further increase transparency in data reporting, as detailed in this editorial (http://www.nature.com/nsmb/journal/v22/n10/full/nsmb.3110.html). Please provide one excel file per figure max, with one panel per tab. When submitting files, the title field should indicate which figure the source data pertains to.

7. DATA AVAILABILITY: this journal strongly supports public availability of data. All data used in accepted papers should be available via a public data repository, or alternatively, as Supplementary Information. If data can only be shared on request, please explain why in your Data Availability Statement and in the correspondence with your editor.

For some data types, deposition in a public repository is mandatory as detailed below:

https://www.nature.com/nature-research/editorial-policies/reporting-standards#availability-of-data

Finally, please ensure that you retain unprocessed data and metadata files after publication, ideally archiving data in perpetuity, as these may be requested during the peer review and production process or after publication if any issues arise.

EXTENDED DATA FIGURES

When re-submitting your manuscript, please ensure that any supplementary figures and tables that are crucial to the manuscript's conclusions are converted into Extended Data figures and tables to increase visibility of these data. Extended Data figures and tables are online-only (present in the online PDF and full-text HTML versions of the paper), peer-reviewed display items that provide essential background to the article but are not included in the main article due to space constraints. A maximum of ten Extended Data display items (figures and tables) is permitted.

While we encourage the use of color in preparing figures, please note that this will incur a charge to partially defray the cost of printing. Information about color charges can be found at http://www.nature.com/nsmb/authors/submit/index.html#costs

Nature Structural & Molecular Biology is committed to improving transparency in authorship. As part of our efforts in this direction, we are now requesting that all authors identified as 'corresponding author' on published papers create and link their Open Researcher and Contributor Identifier (ORCID) with their account on the Manuscript Tracking System (MTS), prior to acceptance. This applies to primary research papers only. ORCID helps the scientific community achieve unambiguous attribution of all scholarly contributions. You can create and link your ORCID from the home page of the MTS by clicking on 'Modify my Springer Nature account'. For more information please visit please visit www.springernature.com/orcid.

Please use the link below to submit your revised manuscript and related files:

Link Redacted

Note: This URL links to your confidential home page and associated information about manuscripts you may have submitted, or that you are reviewing for us. If you wish to forward this email to co-authors, please delete the link to your homepage.

We look forward to seeing the revised manuscript and thank you for the opportunity to review your work.

Sincerely,

Dimitris Typas
Senior Editor
Nature Structural & Molecular Biology
ORCID: 0000-0002-8737-1319

Reviewers' Comments:

Reviewer #1 (Remarks to the Author):

The manuscript reports structural characterizations of the cPRC1 complex, and the results have furthered understandings about the mechanisms of complex assembly, enzymatic activity and genomic targeting of this biologically important protein complex. Overall, the topic is important and of broad interest, the experiments were well designed and executed, the data

were reasonably interpreted and the findings are insightful. The rigorousness of the manuscript may be improved by addressing the following suggestions:

1) Page5, the 1st section of Results: It's not rigorous to compare the enzymatic activity of the 4-subunit PRC1 complex with that of the truncated RING1B-BMI1 core heterodimer (Fig. 1c, 1d). The E3 ligase activity of the RING1B-BMI1 core complex is different from the full-length heterodimer. The core complex appeared to be more active than the full-length or longer heterodimers (Ref. 23 and PMID 16714294, the latter should also be cited). The earlier observations support the author's point of view, but there is a logic gap to conclude that the PRC1 complex is more active than the RING1B-BMI1 complex. It is advisable to include a direct comparison of enzymatic activities with the full-length RING1B-BMI1 heterodimer.
2) Last paragraph of page 6: The " … is reinforced by specific side-chain interactions (Fig. 2c, d)" sentence and later ones referring to protein residues involve density regions determined at low resolutions (Fig. 1f & Extended Data Fig. 1f), clear warnings about possible ambiguity in interpretation should be given.
3) Judging from Extended Data Fig. 1 & 4, the sample particles appear to have obvious preferred orientations, overcoming this problem may improve the quality of the structure.
4) Extended Data Fig. 6b: The flow chart missed the indication of a "Flip Map" step, as in Extended Data Fig. 1c & 4b.

Reviewer #2 (Remarks to the Author):

In this study, Ciapponi et al use single-particle cryo-electron microscopy to determine the partial structure of human canonical PRC1 holocomplex (RING1B, BMI1, PHC2, CBX7) bound to a mono-nucleosome at 2.7 Å. This demonstrates that cPRC1 adopts a compact structure, stabilised by extensive interactions between the different cPRC1 subunits. Importantly, it also suggests previously undocumented interactions between the PHC2 HD domain and UBCH5C, with PHC2 appearing to cooperate with RING1B and BMI1 in stably positioning UBCH5C on the nucleosome. This raised the possibility that PHC2 may contribute to cPRC1 E3 ligase activity. This was tested enzymatically using reconstituted cPRC1 in which PHC2 was replaced with HD domain fragments, including the putative UBCH5C-interacting region. This demonstrated, at least in vitro, that the UBCH5C-interacting region of PHC2 may enhance E3 ligase activity through a mechanism that appears to be independent of nucleosome binding (likely by stabilising UBCH5C positioning/engagement).

In the final two figures, the authors switch their attention to the Drosophila cPRC1 complex, comprising Sce (RING1B), Psc (BMI1), Ph (PHC), and Pc (CBX). Using vitro reconstitutions and affinity purifications, coupled with AF3 modelling, evidence is provided that Drosophila cPRC1 likely forms a similar structure to the human complex. Transgene experiments in Drosophila embryos show that the HD domain of Ph is important for Ph incorporation into cPRC1. In parallel, the contribution of the SAM domain of Ph to cPRC1 targeting to polycomb responsive elements (PREs) is tested. This demonstrates that the SAM domain of Ph is dispensable for cPRC1 complex assembly, but required for targeting of the complex to PREs and thus appropriate in H2A mono-ubiquitylation.

Overall, the structural work presented here is very nicely executed and interesting, potentially providing important new mechanistic insight into how the cPRC1 homocomplex forms (in both human and Drosophila) and its engagement with a mono-nucleosome substrates to drive H2A mono-ubiquitylation. In particular, the contribution of PHC2 to positioning/stabilisation of the UBCH5C ubiquitin-conjugating enzyme will be of interest to Polycomb/chromatin biology field. However, it remains unclear what the relevance of this newly proposed mechanism for PHC2-dependent modulation of enzymatic activity is for H2A mono-ubiquitylation in cells or embryos. Furthermore, the final figure which focusses on the SAM domain of PHC2 (which is missing from the Cryo-EM reconstruction) and how it targets cPRC1 to PRE's seems out of context as it does not directly test mechanistic predictions that emerge from the newly acquired structure. Our main comments are discussed below:

1) From the Cryo-EM reconstruction of cPRC1 engaged with a mono-nucleosome, a number of novel interactions between the PHC2 HD domain and the UBCH5C ubiquitin-conjugating enzyme are apparent, raising the possibility that PHC2 may contribute to PRC1 E3 ligase activity. The authors provide evidence that this is indeed the case, by reconstituting cPRC1 with two different regions of the HD domain and performing in vitro ubiquitylation assays. However, neither of these completely rescues E3 ligase activity of the cPRC1, while nucleosome binding is also greatly affected when the complex only contains small sections of the HD domain. This makes it difficult to understand the precise contribution of the PHC2 HD domain to E3 ligase activity versus nucleosome binding. What would happen in these in vitro assays if the authors used full-length PHC2 with more subtle mutations to the HD domain (based on the structure) that just perturbed the interaction with UBCH5C (see also below)?

2) In the author's Cryo-EM reconstruction, density is only resolved for a relatively small proportion of PHC2, namely the HD domain and immediate flanking regions. The remainder of PHC2, including the FCS and SAM domains, are missing from this reconstruction. In the Drosophila aspect of this study, the authors show that Ph (the Drosophila homologue of PHC2) with its SAM domain deleted is able to form an intact cPRC1 complex (Figure 4), but that this fails to bind at its normal target sites in vivo (PREs) and that there is a reduction of H2A mono-ubiquitylation from these regions. This is consistent with previous proposals that the Ph SAM domain acts as a key tether for cPRC1 at PREs (Isono et al 2013, Frey et al 2016). As such, the effects that the authors show in Figure 5 (failure of cPRC1 to bind PREs and a reduction in H2Aub1 at PREs) and in Extended Figure 8 (embryo defects) are almost certainly due to a failure to appropriately target cPRC1 to PREs. As discussed above, the Cryo-EM structure of the cPRC1 holo-complex bound to a mono-nucleosome provides intriguing new insight into how PHC2/Ph might contribute to cPRC1 E3 ligase activity, but the Drosophila embryo studies performed do not test these new ideas. Could the authors not use information from their Cryo-EM reconstructions coupled with more in-depth

in vitro characterisation (see above), to make more subtle mutations to the Ph HD domain, such that incorporation into cPRC1 is still possible, yet interaction with the ubiquitin-conjugating enzyme (and possible stimulation of H2A mono-ubiquitylation) is specifically perturbed?

3) A related point, we thought the title of this manuscript, ‘Structure of a human PRC1 holocomplex reveals basis of enzymatic activity and genomic targeting’ was somewhat misleading. While we agree that the authors reconstruction potentially provides important insight into the mechanism of cPRC1 E3 ligase activity, it does not reveal anything about the basis of genomic targeting.

Minor points
1) In Figure 1 B-D why is the E3 ligase activity of the cPRC1 complex containing PHC2 and CBX7 compared to a minimal truncated RING1B/BMI1 RING/RING dimer as opposed to the full length RING1B/BMI1 dimer? This is the appropriate comparison as removing the C-termini of RING1B and BMI1 could in theory explain the differences in catalysis observed. As such, it is currently unclear the true magnitude of stimulation that formation of the cPRC1 holo-complex achieves compared the enzymatic core of the full length RING1B/BMI1 dimer.

2) It was unclear from the text/figures how similar/different the observed engagement of cPRC1 and UBCH5C is with the nucleosome compared to the structures of McGinty RK et al 2014. Does the current structure provide more information about nucleosome binding beyond the initial structure?

3) a typo throughout – ‘Extended Data’ is misspelled (Exteneded Data).

Neil Blackledge and Rob Klose

Reviewer #3 (Remarks to the Author):

Ciapponi et al. report the cryo-EM structure of the 4-subunit human cPRC1 in complex with the nucleosome and with and without the UBC5HC E2 ubiquitin conjugating enzyme. cPRC1 plays a central role in silencing of Polycomb target genes but previous structural information on the complex was limited to the RING1B-BMI1 (PCGF4) ubiquination module. The authors show that cPRC1 forms a compact architecture in which the RING1B, BMI1, and PCH2 subunits interact with and position UBCH5C on the nucleosome to ubiquitinate H2AK119. They further use structural modeling to show that his organization is conserved in the Drosophila cPRC1, the full cPRC1 complex is much more active in H2AK119 ubiquitination relative to the core ubiquitination module, and the Drosophila PHC2 homolog Polyhomeotic (Ph) acts as a scaffold for complex assembly via interactions involving its PHD domain, whereas the Ph SAM domain is dispensable for complex assembly but is required for interaction with PhoRC and targeting of cPRC1 to Polycomb target genes. These results and the authors main conclusions are highly valuable and provide a molecular and structural basis for cPRC1-mediated H2AK119 ubiquitination and its recruitment to Polycomb target genes. The results should be of wide interest and are suitable for publication in NSMB.

The structural and other data in the manuscript are of very high technical quality. I only have general comments regarding data interpretation and the relationship between the cPRC1 targeting mechanisms in Drosophila and human.

The authors identify roles for the PHD and SAM domains of PHC2 (Ph) in assembly of the cPRC1 complex and its recruitment to target genes, respectively. However, the SAM domain interactions involve the Drosophila PhoRC, which binds to PREs. Neither PREs nor PhoRC appear to play roles in Polycomb silencing in human cells. So, I am not sure about the title stating, “Structure of a human PRC1 holocomplex reveals basis of enzymatic activity and genomic targeting”. The first part of the title applies to both the human and Drosophila systems and is supported by the data. The second part referring to genomic targeting may apply only to the Drosophila system. Is there an equivalent role for the SAM domain of human PHC proteins in interactions with Polycomb accessory proteins such as KDM2B that target PRC1 to CpG islands?

It is surprising that PRC1 has similar affinity for unmodified and H3K27me3 nucleosomes (ED Figure 6g, h). In the Discussion, the authors suggest that other CBX proteins and/or accessory factor might provide additional binding modes. Is it possible that CBX7 specificity will only be observed in the context of binding to oligonucleosomes with cPRC1 that can oligomerize?

Version 2:

Decision Letter:

Our ref: NSMB-A52664B

26th Jul 2026

Dear Dr. Mueller,

Thank you for submitting your revised manuscript "Cryo-EM structure, enzymatic activity and genome targeting of canonical

PRC1" (NSMB-A52664B). It has now been seen by the original referees and their comments are below. The reviewers find that the paper has improved in revision, and therefore we can now accept it in principle for publication in Nature Structural & Molecular Biology, pending minor revisions to respond to the referees' final requests, particularly the points raised by Reviewer #2, and to comply with our editorial and formatting guidelines.

We are now performing detailed checks on your paper and will send you a checklist detailing our editorial and formatting requirements in a couple of weeks. Please do not upload the final materials and make any revisions until you receive this additional information from us.

Thank you again for your interest in Nature Structural & Molecular Biology Please do not hesitate to contact me if you have any questions.

Sincerely,

Dimitris Typas
Senior Editor
Nature Structural & Molecular Biology
ORCID: 0000-0002-8737-1319

Reviewer #1 (Remarks to the Author):

The revision has addressed my concerns in full. I now recommend acceptance of the manuscript for publication.

Reviewer #2 (Remarks to the Author):

We are for the most part satisfied that the authors have adequately addressed the points we raised in their revised manuscript. In particular, testing the contribution of the PHC2 HD domain to PRC1 catalytic activity using more subtle point mutations (Figure 3F and G), is far more informative than the large truncations to PHC2 used in the original manuscript. These new data conclusively show that, at least in vitro, this region of the PHC2 HD domain significantly contributes to catalysis. However, the one point that the authors did not address was testing
the relevance of the PHC2 HD domain to H2A monoubiquitylation in vivo. We appreciate that making and properly characterising new Drosophila lines to definitively test this might take 12-18 months and be ‘beyond the scope’ of this study. However, with that in mind, we feel the authors should mention this limitation to the current study and state that future work will be required to test the relevance of the Ph/PHC2 HD domain to catalytic activity in vivo.

Rob Klose and Neil Blackledge

Reviewer #3 (Remarks to the Author):

The authors have responded to my comments satisfactorily and I am very happy to support publication in NSMB.

INTERNAL USE ONLY AIP modules required: ["Life Science"]

Version 3:

Decision Letter:

14th Aug 2026

Dear Dr. Mueller,

We are now happy to accept your revised paper "Cryo-EM structure, enzymatic activity and genome targeting of canonical PRC1" for publication as an Article in Nature Structural & Molecular Biology.

Acceptance is conditional on the manuscript's not being published elsewhere and on there being no announcement of this work to the newspapers, magazines, radio or television until the publication date in Nature Structural & Molecular Biology.

Over the next few weeks, your paper will be copyedited to ensure that it conforms to Nature Structural & Molecular Biology style. Once your paper is typeset, you will receive an email with a link to choose the appropriate publishing options for your paper and our Author Services team will be in touch regarding any additional information that may be required.

After the grant of rights is completed, you will receive a link to your electronic proof via email with a request to make any corrections within 48 hours. If, when you receive your proof, you cannot meet this deadline, please inform us at rjsproduction@springernature.com immediately.

You will not receive your proofs until the publishing agreement has been received through our system.

Due to the importance of these deadlines, we ask that you please let us know now whether you will be difficult to contact over the next month. If this is the case, we ask you provide us with the contact information (email, phone and fax) of someone who will be able to check the proofs on your behalf, and who will be available to address any last-minute problems.

To assist our authors in disseminating their research to the broader community, our SharedIt initiative provides all co-authors with the ability to generate a unique shareable link that will allow anyone (with or without a subscription) to read the published article. Recipients of the link with a subscription will also be able to download and print the PDF.

As soon as your article is published, you can generate your shareable link by entering the DOI of your article here: http://authors.springernature.com/share. Corresponding authors will also receive an automated email with the shareable link

Note the policy of the journal on data deposition: http://www.nature.com/authors/policies/availability.html.

Your paper will be published online soon after we receive proof corrections and will appear in print in the next available issue. You can find out your date of online publication by contacting the production team shortly after sending your proof corrections.

You may wish to make your media relations office aware of your accepted publication, in case they consider it appropriate to organize some internal or external publicity. Once your paper has been scheduled you will receive an email confirming the publication details. This is normally 3-4 working days in advance of publication. If you need additional notice of the date and time of publication, please let the production team know when you receive the proof of your article to ensure there is sufficient time to coordinate. Further information on our embargo policies can be found here: https://www.nature.com/authors/policies/embargo.html

You can now use a single sign-on for all your accounts, view the status of all your manuscript submissions and reviews, access usage statistics for your published articles and download a record of your refereeing activity for the Nature journals.

If you have not already done so, we strongly recommend that you upload the step-by-step protocols used in this manuscript to the Protocol Exchange. Protocol Exchange is an open online resource that allows researchers to share their detailed experimental know-how. All uploaded protocols are made freely available, assigned DOIs for ease of citation and fully searchable through nature.com. Protocols can be linked to any publications in which they are used and will be linked to from your article. You can also establish a dedicated page to collect all your lab Protocols. By uploading your Protocols to Protocol Exchange, you are enabling researchers to more readily reproduce or adapt the methodology you use, as well as increasing the visibility of your protocols and papers. Upload your Protocols at www.nature.com/protocolexchange/. Further information can be found at www.nature.com/protocolexchange/about.

An online order form for reprints of your paper is available at https://www.nature.com/reprints/author-reprints.html. Please let your coauthors and your institutions' public affairs office know that they are also welcome to order reprints by this method.

Authors may need to take specific actions to achieve compliance with funder and institutional open access mandates. If your research is supported by a funder that requires immediate open access (e.g. according to Plan S principles or the NIH public access policy) then you should select the gold OA route, and we will direct you to the compliant route where possible. Because authors warrant under our subscription licensing terms that they haven't committed to licensing any version of their article under a licence inconsistent with the terms of our agreement – including the applicable embargo period – publication under the subscription model isn't suitable for authors whose funders require no embargo.

In approximately 10 business days you will receive an email with a link to choose the appropriate publishing options for your paper and our Author Services team will be in touch regarding any additional information that may be required.

You will not receive your proofs until the publishing agreement has been received through our system.

If you have any questions about our publishing options, costs, Open Access requirements, or our legal forms, please contact ASJournals@springernature.com

Sincerely,

Dimitris Typas

Senior Editor
Nature Structural & Molecular Biology
ORCID: 0000-0002-8737-1319

Click here if you would like to recommend Nature Structural & Molecular Biology to your librarian:
http://www.nature.com/subscriptions/recommend.html#forms

Open Access This Peer Review File is licensed under a Creative Commons Attribution 4.0 International License, which permits use, sharing, adaptation, distribution and reproduction in any medium or format, as long as you give appropriate credit to the original author(s) and the source, provide a link to the Creative Commons license, and indicate if changes were made.
In cases where reviewers are anonymous, credit should be given to 'Anonymous Referee' and the source.
The images or other third party material in this Peer Review File are included in the article’s Creative Commons license, unless indicated otherwise in a credit line to the material. If material is not included in the article’s Creative Commons license and your intended use is not permitted by statutory regulation or exceeds the permitted use, you will need to obtain permission directly from the copyright holder.
To view a copy of this license, visit https://creativecommons.org/licenses/by/4.0/

Point-by-point response to reviewer comments

REVIEWER #1

The manuscript reports structural characterizations of the cPRC1 complex, and the results have furthered understandings about the mechanisms of complex assembly, enzymatic activity and genomic targeting of this biologically important protein complex. Overall, the topic is important and of broad interest, the experiments were well designed and executed, the data were reasonably interpreted and the findings are insightful. The rigorousness of the manuscript may be improved by addressing the following suggestions.

Thank you for your kind words and support.

Point 1, the reviewer wrote:
Page5, the 1st section of Results: It's not rigorous to compare the enzymatic activity of the 4-subunit PRC1 complex with that of the truncated RING1B-BMI1 core heterodimer (Fig. 1c, 1d). The E3 ligase activity of the RING1B-BMI1 core complex is different from the full-length heterodimer. The core complex appeared to be more active than the full-length or longer heterodimers (Ref. 23 and PMID 16714294, the latter should also be cited). The earlier observations support the author's point of view, but there is a logic gap to conclude that the PRC1 complex is more active than the RING1B-BMI1 complex. It is advisable to include a direct comparison of enzymatic activities with the full-length RING1B-BMI1 heterodimer.

<u>Our response</u>:
We thank the reviewer for this helpful suggestion. This issue was also raised by reviewer #2. Although our original comparison with the minimal RING1B–BMI1 Ring finger heterodimer was motivated by the previously reported higher catalytic activity of this construct (Buchwald et al., 2006; Li et al., 2006), we agree that the full-length RING1B–BMI1 heterodimer provides the more appropriate benchmark for evaluating the activity of the PRC1 holocomplex. We have therefore performed the requested experiment and now present a direct comparison of the enzymatic activities of the PRC1 holocomplex and the full-length RING1B–BMI1 heterodimer in the revised **Fig. 1b, c**. The original comparison with the minimal RING1B–BMI1 Ring finger heterodimer has been moved to **Extended Data Fig. 1c–e**.

The new data show that the PRC1 holocomplex is substantially more active than the full-length RING1B–BMI1 heterodimer. They also corroborate the previous observations of Buchwald et al. (2006) and Li et al. (2006) that the minimal RING1B–BMI1 Ring finger heterodimer exhibits higher E3 ligase activity than the full-length RING1B–BMI1 heterodimer.

We have also added the citation to Li et al. (2006). We thank the reviewer for pointing this out.

Overall, the revised experiments show that the PRC1 holocomplex is more active than both the full-length RING1B–BMI1 heterodimer and the minimal Ring finger heterodimer, supporting our conclusion that PRC1 subunits beyond the catalytic RING dimer enhance ubiquitin ligase activity.

Point 2, the reviewer wrote:
Last paragraph of page 6: The " … is reinforced by specific side-chain interactions (Fig. 2c, d)" sentence and later ones referring to protein residues involve density regions

determined at low resolutions (Fig. 1f & Extended Data Fig. 1f), clear warnings about possible ambiguity in interpretation should be given.

Our response:
We thank the reviewer for raising concerns about the interpretation of structural features in regions of lower local resolution. Although the overall reconstruction contains regions of heterogeneous local resolution, the β-sheet interface between the PRC1 subunits discussed in **Fig. 2c, d** is supported by comparatively strong density. To facilitate evaluation of this region, we have added a close-up view of the β-sheet interface in **Extended Data Fig. 2f**, which also illustrates the local variation in map quality.

Within this interface, only the side chain of CBX7 Asp131 is supported by weaker density. Accordingly, this residue is shown as a line representation in **Fig. 2d**, and this is now explicitly stated in the figure legend.

We agree that other regions of the structure, particularly the RING1B RAWUL domain and the N-terminal region of CBX7, are resolved at lower local resolution (**Extended Data Fig. 2f**). To avoid overinterpretation, we have revised the text throughout the manuscript, including the legends to **Fig. 2e,f**, to use more cautious language when describing side-chain interactions. In addition, residues supported by weaker density are consistently shown as line representations in all structural figures.

Point 3, the reviewer wrote:
Judging from Extended Data Fig. 1 & 4, the sample particles appear to have obvious preferred orientations, overcoming this problem may improve the quality of the structure.

Our response:
To assess the potential impact of preferred particle orientations, we performed the Orientation Diagnostics analysis implemented in CryoSPARC v4.4, which provides a quantitative assessment of orientation bias based on 3DFSC (Tan et al., 2017) and Fourier sampling analysis (Baldwin & Lyumkis, 2020). We have added the corresponding relative signal amount (Fourier-space coverage) plots for each reconstruction presented in this study (**Extended Data Fig. 2e, Extended Data Fig. 5e and Extended Data Fig. 7d**).

As noted by the reviewer, the particle orientation distributions show a preference for certain viewing directions. This is most likely attributable to interactions of the particles with the grid surface and/or the air–water interface during sample preparation, leading to a non-uniform distribution of particle orientations. However, despite this bias, the relative signal amount remains broadly distributed in Fourier space, with no severely under-sampled regions that would compromise the quality of the reconstructions (**Extended Data Fig. 2e, Extended Data Fig. 5e and Extended Data Fig. 7d**). The Fourier-space coverage is therefore sufficient to support reliable 3D reconstructions.

Consistent with this analysis, the nucleosome core region is well resolved and shows no evidence of directional anisotropy. Regions with lower local resolution are largely confined to flexible elements, including the DNA ends and the upper lobe of PRC1, consistent with conformational heterogeneity rather than insufficient angular sampling.
Taken together, these analyses indicate that although the particles exhibit preferred orientations, this does not adversely affect the quality or interpretation of the final reconstructions.

Point 4, the reviewer wrote:
Extended Data Fig. 6b: The flow chart missed the indication of a “Flip Map” step, as in Extended Data Fig. 1c & 4b.

<u>Our response</u>:
We have added the missing "Flip Map" step to the flow chart in the revised **Extended Data Fig. 7b.**

****

REVIEWER #2

In this study, Ciapponi et al use single-particle cryo-electron microscopy to determine the partial structure of human canonical PRC1 holocomplex (RING1B, BMI1, PHC2, CBX7) bound to a mono-nucleosome at 2.7 Å. This demonstrates that cPRC1 adopts a compact structure, stabilised by extensive interactions between the different cPRC1 subunits. Importantly, it also suggests previously undocumented interactions between the PHC2 HD domain and UBCH5C, with PHC2 appearing to cooperate with RING1B and BMI1 in stably positioning UBCH5C on the nucleosome. This raised the possibility that PHC2 may contribute to cPRC1 E3 ligase activity. This was tested enzymatically using reconstituted cPRC1 in which PHC2 was replaced with HD domain fragments, including the putative UBCH5C-interacting region. This demonstrated, at least in vitro, that the UBCH5C-interacting region of PHC2 may enhance E3 ligase activity through a mechanism that appears to be independent of nucleosome binding (likely by stabilising UBCH5C positioning/engagement).

In the final two figures, the authors switch their attention to the Drosophila cPRC1 complex, comprising Sce (RING1B), Psc (BMI1), Ph (PHC), and Pc (CBX). Using vitro reconstitutions and affinity purifications, coupled with AF3 modelling, evidence is provided that Drosophila cPRC1 likely forms a similar structure to the human complex. Transgene experiments in Drosophila embryos show that the HD domain of Ph is important for Ph incorporation into cPRC1. In parallel, the contribution of the SAM domain of Ph to cPRC1 targeting to polycomb responsive elements (PREs) is tested. This demonstrates that the SAM domain of Ph is dispensable for cPRC1 complex assembly, but required for targeting of the complex to PREs and thus appropriate in H2A mono-ubiquitylation.

Overall, the structural work presented here is very nicely executed and interesting, potentially providing important new mechanistic insight into how the cPRC1 homocomplex forms (in both human and Drosophila) and its engagement with a mono-nucleosome substrates to drive H2A mono-ubiquitylation. In particular, the contribution of PHC2 to positioning/stabilisation of the UBCH5C ubiquitin-conjugating enzyme will be of interest to Polycomb/chromatin biology field. However, it remains unclear what the relevance of this newly proposed mechanism for PHC2-dependent modulation of enzymatic activity is for H2A mono-ubiquitylation in cells or embryos. Furthermore, the final figure which focusses on the SAM domain of PHC2 (which is missing from the Cryo-EM reconstruction) and how it targets cPRC1 to PRE’s seems out of context as it does not directly test mechanistic predictions that emerge from the newly acquired structure.

Thank you for your kind words and support.

Point 1, the reviewer wrote:
From the Cryo-EM reconstruction of cPRC1 engaged with a mono-nucleosome, a number of novel interactions between the PHC2 HD domain and the UBCH5C ubiquitin-conjugating enzyme are apparent, raising the possibility that PHC2 may contribute to PRC1 E3 ligase activity. The authors provide evidence that this is indeed the case, by reconstituting cPRC1 with two different regions of the HD domain and performing in vitro ubiquitylation assays. However, neither of these completely rescues E3 ligase activity of the cPRC1, while nucleosome binding is also greatly affected when the complex only contains small sections of the HD domain. This makes it difficult to understand the precise contribution of the PHC2 HD domain to E3 ligase activity versus nucleosome binding. What would happen in these in vitro assays if the authors used full-length PHC2 with more subtle mutations to the HD domain (based on the structure) that just perturbed the interaction with UBCH5C (see also below)?

Our response:
To address this point, we reconstituted human cPRC1 holocomplexes containing full-length PHC2 with point mutations designed to perturb the putative interaction between the PHC2 HD domain and UBCH5C, guided by our structural model and cross-linking data. Specifically, we mutated PHC2 residues 57–60 (LLVG) to alanine ($PHC2_{4A}$). A cPRC1($PHC2_{4A}$) holocomplex containing stoichiometric amounts of all four subunits was purified to homogeneity (**Extended Data Fig. 6d,e**), and its activity was compared with that of the wild-type cPRC1 holocomplex. We also designed several additional PHC2 point mutants, but these impaired PHC2 expression and therefore did not permit purification of stoichiometric PRC1 holocomplexes.
The PRC1($PHC2_{4A}$) mutant complex exhibited more than a twofold reduction in E3 ligase activity compared with wild-type PRC1 (**Fig. 3f,g**). Importantly, cPRC1($PHC2_{4A}$) bound mononucleosomes with an affinity comparable to that of the wild-type complex (**Fig. 3h,i**). These results demonstrate that the PHC2 HD domain directly contributes to cPRC1 E3 ligase activity through its interaction with UBCH5C, independently of an effect on nucleosome binding.

The $PHC2_{4A}$ point mutant provides a more specific and mechanistically informative means of assessing the contribution of the PHC2 HD domain than the previously analyzed PHC2 truncations ($PHC2_{28–53}$ and $PHC2_{28–69}$). We therefore replaced the truncation mutant experiments in the revised manuscript with the new point-mutant analyses.

Point 2, the reviewer wrote:
In the author's Cryo-EM reconstruction, density is only resolved for a relatively small proportion of PHC2, namely the HD domain and immediate flanking regions. The remainder of PHC2, including the FCS and SAM domains, are missing from this reconstruction. In the Drosophila aspect of this study, the authors show that Ph (the Drosophila homologue of PHC2) with its SAM domain deleted is able to form an intact cPRC1 complex (Figure 4), but that this fails to bind at its normal target sites in vivo (PREs) and that there is a reduction of H2A mono-ubiquitylation from these regions. This is consistent with previous proposals that the Ph SAM domain acts as a key tether for cPRC1 at PREs (Isono et al 2013, Frey et al 2016). As such, the effects that the authors show in Figure 5 (failure of cPRC1 to bind PREs and a reduction in H2Aub1 at PREs) and in Extended Figure 8 (embryo defects) are almost certainly due to a failure

to appropriately target cPRC1 to PREs. As discussed above, the Cryo-EM structure of the cPRC1 holo-complex bound to a mono-nucleosome provides intriguing new insight into how PHC2/Ph might contribute to cPRC1 E3 ligase activity, but the Drosophila embryo studies performed do not test these new ideas. Could the authors not use information from their Cryo-EM reconstructions coupled with more in-depth in vitro characterisation (see above), to make more subtle mutations to the Ph HD domain, such that incorporation into cPRC1 is still possible, yet interaction with the ubiquitin-conjugating enzyme (and possible stimulation of H2A mono-ubiquitylation) is specifically perturbed?

Our response:
We have strengthened the analysis of cPRC1 recruitment in *Drosophila* by profiling the PRC1 core subunit Psc. Like V5-$Ph_{\Delta SAM}$, Psc binding is no longer detected at PREs in $Ph^{\Delta SAM}$ mutant embryos, providing independent support for the conclusion that deletion of the Ph SAM domain abolishes cPRC1 recruitment to PREs (**Fig. 5a,b**).

We agree with the reviewer that generating *Drosophila* lines carrying subtle point mutations in the Ph HD domain designed to selectively disrupt UbcH5c interaction while preserving cPRC1 complex integrity would provide an informative *in vivo* test of the structural model. However, generating the corresponding genome-edited fly lines and comprehensively characterizing their effects on cPRC1 function, H2Aub1 deposition, chromatin binding, and developmental phenotypes would require approximately 12–18 months. We therefore consider these experiments to be beyond the scope of the revision.

Point 3, the reviewer wrote:
A related point, we thought the title of this manuscript, 'Structure of a human PRC1 holocomplex reveals basis of enzymatic activity and genomic targeting' was somewhat misleading. While we agree that the authors reconstruction potentially provides important insight into the mechanism of cPRC1 E3 ligase activity, it does not reveal anything about the basis of genomic targeting.

Our response:
We agree with the reviewer that the original title was misleading because the cryo-EM structure presented in this study does not reveal the mechanism of genomic targeting in mammalian cells. Rather, it defines the previously unknown architecture of the human cPRC1 holocomplex and provides mechanistic insight into its E3 ligase activity. The second part of the study uses *Drosophila* genetics to demonstrate that the Ph SAM domain, which is absent from the cryo-EM reconstruction of the human cPRC1 holocomplex, is dispensable for cPRC1 assembly but essential for genomic targeting to Polycomb Response Elements. To more accurately reflect the overall scope of the work, we have revised the title to **"Cryo-EM structure, enzymatic activity and genome targeting of canonical PRC1."**

Minor point 1, the reviewer wrote:
In Figure 1 B-D why is the E3 ligase activity of the cPRC1 complex containing PHC2 and CBX7 compared to a minimal truncated RING1B/BMI1 RING/RING dimer as opposed to the full length RING1B/BMI1 dimer? This is the appropriate comparison as removing the C-termini of RING1B and BMI1 could in theory explain the differences in catalysis observed. As such, it is currently unclear the true magnitude of stimulation that formation of the cPRC1 holo-complex achieves compared the enzymatic core of the full length RING1B/BMI1 dimer.

Our response:
We thank the reviewer for this helpful suggestion. As also discussed in response to point 1 of reviewer #1, our original comparison with the minimal RING1B–BMI1 Ring finger heterodimer was motivated by the previously reported higher catalytic activity of this minimal heterodimer compared to that of the full-length RING1B–BMI1 heterodimer (Buchwald et al., 2006; Li et al., 2006). We agree that the full-length RING1B–BMI1 heterodimer provides the more appropriate benchmark for evaluating the activity of the cPRC1 holocomplex. We have therefore performed the requested experiment and now present a direct comparison of the enzymatic activities of the cPRC1 holocomplex and the full-length RING1B–BMI1 heterodimer in the revised **Fig. 1b,c**. The original comparison with the minimal RING1B–BMI1 Ring finger heterodimer has been moved to **Extended Data Fig. 1c–e**.

The new data show that the cPRC1 holocomplex is substantially more active than the full-length RING1B–BMI1 heterodimer. They also corroborate the previous observations of Buchwald et al. (2006) and Li et al. (2006) that the minimal RING1B–BMI1 Ring finger heterodimer exhibits higher E3 ligase activity than the full-length RING1B–BMI1 heterodimer.

Overall, the revised experiments show that the cPRC1 holocomplex is more active than both the full-length RING1B–BMI1 heterodimer and the minimal Ring finger heterodimer, supporting our conclusion that cPRC1 subunits beyond the catalytic RING dimer enhance ubiquitin ligase activity.

Minor point 2, the reviewer wrote:
It was unclear from the text/figures how similar/different the observed engagement of cPRC1 and UBCH5C is with the nucleosome compared to the structures of McGinty RK et al 2014. Does the current structure provide more information about nucleosome binding beyond the initial structure?

Our response:
The crystal structure reported by McGinty et al. (2014) shows two copies of the minimal RING1B–BMI1 Ring finger heterodimer bound to opposite faces of the nucleosome in a broadly similar manner. In this structure, UBCH5C was covalently fused to the C terminus of the RING1B RING domain. However, the position of UBCH5C differs between the two nucleosome-bound complexes, most likely owing to differences in crystal packing. Thus, the observed position of UBCH5C was likely influenced by its covalent linkage to the nucleosome-bound RING1B–BMI1 heterodimer, rather than being determined solely by its interactions with the nucleosome.
In the original manuscript, we aligned our structure with the proximal nucleosome-bound RING1B–BMI1 Ring finger heterodimer from the McGinty et al. structure and observed a difference in the position of UBCH5C. In response to the reviewer's comment, we have now also aligned our structure with the distal RING1B–BMI1 complex. In this comparison, the position of UBCH5C in our structure closely overlaps with that observed in the distal complex reported by McGinty et al. (**Extended Data Fig. 3a**). Thus, our structure is fully consistent with the distal RING1B–BMI1 Ring finger heterodimer–nucleosome complex, in which UBCH5C is positioned farther from both the histones and the nucleosomal DNA. Overall, our structure does not reveal a substantially different mode of nucleosome engagement.

Minor point 3, the reviewer wrote:
a typo throughout – 'Extended Data' is misspelled (Exteneded Data).

Our response:
We thank the reviewer for pointing out this typographical error. The spelling of "Extended Data" has been corrected throughout the manuscript.

****

REVIEWER #3

Ciapponi et al. report the cryo-EM structure of the 4-subunit human cPRC1 in complex with the nucleosome and with and without the UBC5HC E2 ubiquitin conjugating enzyme. cPRC1 plays a central role in silencing of Polycomb target genes but previous structural information on the complex was limited to the RING1B-BMI1 (PCGF4) ubiquination module. The authors show that cPRC1 forms a compact architecture in which the RING1B, BMI1, and PCH2 subunits interact with and position UBCH5C on the nucleosome to ubiquitinate H2AK119. They further use structural modeling to show that his organization is conserved in the Drosophila cPRC1, the full cPRC1 complex is much more active in H2AK119 ubiquitination relative to the core ubiquitination module, and the Drosophila PHC2 homolog Polyhomeotic (Ph) acts as a scaffold for complex assembly via interactions involving its PHD domain, whereas the Ph SAM domain is dispensable for complex assembly but is required for interaction with PhoRC and targeting of cPRC1 to Polycomb target genes. These results and the authors main conclusions are highly valuable and provide a molecular and structural basis for cPRC1-mediated H2AK119 ubiquitination and its recruitment to Polycomb target genes. The results should be of wide interest and are suitable for publication in NSMB.

The structural and other data in the manuscript are of very high technical quality. I only have general comments regarding data interpretation and the relationship between the cPRC1 targeting mechanisms in Drosophila and human.

Thank you for your kind words and support.

Point 1, the reviewer wrote:
The authors identify roles for the PHD and SAM domains of PHC2 (Ph) in assembly of the cPRC1 complex and its recruitment to target genes, respectively. However, the SAM domain interactions involve the Drosophila PhoRC, which binds to PREs. Neither PREs nor PhoRC appear to play roles in Polycomb silencing in human cells. So, I am not sure about the title stating, "Structure of a human PRC1 holocomplex reveals basis of enzymatic activity and genomic targeting". The first part of the title applies to both the human and Drosophila systems and is supported by the data. The second part referring to genomic targeting may apply only to the Drosophila system. Is there an equivalent role for the SAM domain of human PHC proteins in interactions with Polycomb accessory proteins such as KDM2B that target PRC1 to CpG islands?

Our response:
This point was also raised by reviewer #2 and we agree with the reviewers that the original title was misleading because the cryo-EM structure presented in this study does not reveal the mechanism of genomic targeting in mammalian cells. Rather, it defines the previously unknown architecture of the human cPRC1 holocomplex and provides mechanistic insight into

its E3 ligase activity. The second part of the study uses *Drosophila* genetics to demonstrate that the Ph SAM domain, which is absent from the cryo-EM reconstruction of the human cPRC1 holocomplex, is dispensable for cPRC1 assembly but essential for genomic targeting to Polycomb Response Elements. To more accurately reflect the overall scope of the work, we have revised the title to **"Cryo-EM structure, enzymatic activity and genome targeting of canonical PRC1."**

We further agree that the genomic targeting mechanism addressed in this study is closely related to the *Drosophila* system. In contrast, the mechanisms governing genomic targeting of human canonical PRC1 (cPRC1) remain incompletely understood. As noted by the reviewer, additional recruitment mechanisms have been described for variant PRC1 complexes, including KDM2B-mediated recruitment of PRC1.1 to non-methylated CpG islands. However, KDM2B is specific to variant PRC1.1 and is not a component of canonical PRC1. Thus, whether mammalian PHC proteins contribute to cPRC1 targeting through interactions analogous to those described for *Drosophila* Ph remains an open question.

Point 2, the reviewer wrote:
It is surprising that PRC1 has similar affinity for unmodified and H3K27me3 nucleosomes (ED Figure 6g, h). In the Discussion, the authors suggest that other CBX proteins and/or accessory factor might provide additional binding modes. Is it possible that CBX7 specificity will only be observed in the context of binding to oligonucleosomes with cPRC1 that can oligomerize?

<u>Our response:</u>
We have now replaced the activity and nucleosome-binding assays using PRC1 containing truncated PHC2 (residues 1–69) with experiments using the full-length PRC1 holocomplex (**Extended Data Fig. 7f–i**). Under these conditions, we again did not detect a measurable difference in binding affinity between H3K27me3-modified and unmodified nucleosomes.

We also attempted to reconstitute PRC1 holocomplexes containing CBX2. However, CBX2 underwent partial proteolytic degradation under our experimental conditions, resulting in preparations that did not contain stoichiometric amounts of full-length CBX2. We therefore did not consider these assemblies suitable for a systematic quantitative analysis.

Whether preferential recognition of H3K27me3 becomes more apparent in the context of higher-order chromatin or oligomerization-competent cPRC1 remains an interesting question. However, such experiments would introduce additional variables arising from multivalent PRC1–chromatin interactions and cPRC1 oligomerization, making quantitative interpretation substantially more complex than the mononucleosome-binding assays used here.

Point-by-point response to reviewer comments

REVIEWER #2

The reviewer wrote:
"We are for the most part satisfied that the authors have adequately addressed the points we raised in their revised manuscript. In particular, testing the contribution of the PHC2 HD domain to PRC1 catalytic activity using more subtle point mutations (Figure 3F and G), is far more informative than the large truncations to PHC2 used in the original manuscript. These new data conclusively show that, at least in vitro, this region of the PHC2 HD domain significantly contributes to catalysis. However, the one point that the authors did not address was testing the relevance of the PHC2 HD domain to H2A monoubiquitylation in vivo. We appreciate that making and properly characterising new Drosophila lines to definitively test this might take 12-18 months and be 'beyond the scope' of this study. However, with that in mind, we feel the authors should mention this limitation to the current study and state that future work will be required to test the relevance of the Ph/PHC2 HD domain to catalytic activity in vivo."

<u>Our response:</u>
We thank the reviewer for this suggestion. As requested, we have now explicitly addressed this limitation in both the **Results** and **Discussion** sections by stating that future studies using separation-of-function mutants will be required to determine the relevance of the proposed PHC2/Ph–UBCH5C interaction to PRC1-mediated H2A monoubiquitination in vivo.

In the **Results** section, we added the following sentence:
"Similar separation-of-function mutants in PHC2 will be needed to determine whether the proposed PHC2–UBCH5C interaction contributes to PRC1-mediated H2A monoubiquitination in vivo." (lines 199–201)

In the **Discussion** section, we added the following sentence:
"Whether the PHC2/Ph–UBCH5C interaction contributes to PRC1-mediated H2A monoubiquitination in vivo remains to be determined by analysing separation-of-function mutants that selectively disrupt this interaction." (lines 351–354)
